# Comparative analysis of mitochondrion-related organelles in anaerobic amoebozoans

**DOI:** 10.1099/mgen.0.001143

**Published:** 2023-11-23

**Authors:** Kristína Záhonová, Zoltán Füssy, Courtney W. Stairs, Michelle M. Leger, Jan Tachezy, Ivan Čepička, Andrew J. Roger, Vladimír Hampl

**Affiliations:** ^1^​ Department of Parasitology, Faculty of Science, Charles University, BIOCEV, Vestec, Czechia; ^2^​ Institute of Parasitology, Biology Centre, Czech Academy of Sciences, České Budějovice (Budweis), Czechia; ^3^​ Life Science Research Centre, Department of Biology and Ecology, Faculty of Science, University of Ostrava, Ostrava, Czechia; ^4^​ Division of Infectious Diseases, Department of Medicine, Faculty of Medicine and Dentistry, University of Alberta, Edmonton, Canada; ^5^​ Centre for Comparative Genomics and Evolutionary Bioinformatics, and Department of Biochemistry and Molecular Biology, Dalhousie University, Halifax, Canada; ^6^​ Department of Zoology, Faculty of Science, Charles University, Prague, Czechia; ^‡^​ Present address: Microbiology Research Group, Department of Biology, Lund University, Lund, Sweden; ^§^​ Present address: Institute of Evolutionary Biology (CSIC-Universitat Pompeu Fabra), Barcelona, Spain

**Keywords:** reductive evolution, mitochondrion-related organelles, anaerobiosis, comparative genomics

## Abstract

Archamoebae comprises free-living or endobiotic amoebiform protists that inhabit anaerobic or microaerophilic environments and possess mitochondrion-related organelles (MROs) adapted to function anaerobically. We compared *in silico* reconstructed MRO proteomes of eight species (six genera) and found that the common ancestor of Archamoebae possessed very few typical components of the protein translocation machinery, electron transport chain and tricarboxylic acid cycle. On the other hand, it contained a sulphate activation pathway and bacterial iron–sulphur (Fe-S) assembly system of MIS-type. The metabolic capacity of the MROs, however, varies markedly within this clade. The glycine cleavage system is widely conserved among Archamoebae, except in *Entamoeba*, probably owing to its role in catabolic function or one-carbon metabolism. MRO-based pyruvate metabolism was dispensed within subgroups Entamoebidae and Rhizomastixidae, whereas sulphate activation could have been lost in isolated cases of *Rhizomastix libera*, *Mastigamoeba abducta* and *Endolimax* sp. The MIS (Fe-S) assembly system was duplicated in the common ancestor of Mastigamoebidae and Pelomyxidae, and one of the copies took over Fe-S assembly in their MRO. In Entamoebidae and Rhizomastixidae, we hypothesize that Fe-S cluster assembly in both compartments may be facilitated by dual localization of the single system. We could not find evidence for changes in metabolic functions of the MRO in response to changes in habitat; it appears that such environmental drivers do not strongly affect MRO reduction in this group of eukaryotes.

## Data Summary

Raw RNA sequencing reads and transcriptome assemblies are deposited in NCBI under BioProjects PRJNA935293 and PRJNA380424. HMM profiles of the protein translocation machinery components are available on Figshare under the following link: https://figshare.com/projects/MRO-logy_of_Archamoebae_species/160169.

**Impact Statement**

Mitochondria, keystone organelles of eukaryotic cells, are often modified to reflect environmental conditions. Yet, our knowledge of the paths and processes underlying the adaptation to a low-oxygen environment is still limited. In this work, we compare predicted mitochondrial proteomes of eight species of anaerobic Archamoebae from multiple lineages within this group and of various lifestyles – free living, endobiotic and pathogenic. We reconstructed the features of the common ancestor and followed the modifications through losses and acquisitions. We infer that the evolution of the functions did not follow a lifestyle-specific pattern but rather a phylogenetic pattern in which the changes were specific to phylogenetic lineages.

## Introduction

Mitochondria are keystone organelles of eukaryotes that revolutionized energy conversion in eukaryogenesis. The site of essential metabolic pathways, mitochondria are best known for their role in aerobic oxidation through the concerted action of the respiratory chain and the tricarboxylic acid cycle (TCA). Catabolic pathways in mitochondria, such as branched-chain amino acid metabolism and beta-oxidation of fatty acids, allow balancing the cellular need for ATP and NAD(P)H. These metabolic processes require a broad repertoire of proteins, cofactors and metabolites, some synthesized *in situ* and others transported inside by dedicated metabolite transporters. For proteins, there are a complex import machines known as translocons. Most experimentally established mitochondrial proteomes consist of many hundreds to thousands of proteins [1–6].

In anaerobic and microaerophilic organisms, mitochondria are often modified reflecting the conditions imposed by their niche. These anaerobically adapted mitochondria are collectively referred to as mitochondrion-related organelles (MROs) and their functions range from multifaceted energy metabolism in hydrogen-producing mitochondria to mere cofactor synthesis mitosomes [7–9]. Consistently, MRO proteomes are smaller and altered in comparison with those of canonical aerobic mitochondria [10–17]. So far, only one lineage of eukaryotes has been shown to have completely lost mitochondria [18], highlighting the importance of these organelles.

The diversity of eukaryotes from microaerobic and anaerobic habitats, such as fresh water and ocean sediments, the intestinal tract of animals, deep soil or landfills, is tremendous with representatives found within most major eukaryotic groups [9]. Yet, our knowledge of the adaptation to these environments is still limited. Here, we focus on Archamoebae, a lineage of exclusively anaerobic and microaerophilic species originally considered to lack mitochondria [19] but for which there are now numerous reports demonstrating the presence of MROs in some of them [20–25]. The minimalistic mitosome of *Entamoeba histolytica* is involved in sulphate activation via 3′-phosphoadenosine-5′-phosphosulfate (PAPS), essential in sulpholipid production linked to both proliferation and encystation [26, 27]. The MROs of *Mastigamoeba balamuthi* and *Pelomyxa schiedti* additionally house metabolic pathways connected to glycine cleavage, iron–sulphur (Fe-S) cluster synthesis, and pyruvate metabolism concomitant to hydrogen production [28–30]. To elucidate trends shaping the biology and metabolism of mitochondria, we compare these three MROs to organelles of five additional Archamoebae species from diverse habitats that represent multiple lineages of this clade [31].

## Methods

### Sampling and cultivation conditions

The origin and culture conditions of strains IND8 of *Rhizomastix libera*, GOL1 of *Rhizomastix vacuolata*, CHOM1 of *Mastigamoeba abducta* and ATCC50342 of *Mastigella eilhardi* are described elsewhere [22–24]. A monoeukaryotic polyxenic culture of *Endolimax* sp. ZUBR was derived from the culture of *Tetratrichomonas* sp. ZUBR and cultured as described for the original culture [32].

### RNA isolation and sequencing

Cells of *R. vacuolata* and *Endolimax* sp. ZUBR were harvested from resuspended cultures at 1500 *g* to remove excess bacteria. Total RNA was isolated using TRI Reagent (Sigma-Aldrich) and enriched for poly-adenylated mRNA using the Dynabeads mRNA Purification Kit (Thermo-Fisher) following the manufacturer’s instructions. RNA sequencing (RNA-seq) libraries were prepared using the NEBNext Ultra II RNA Library Kit (New England Biolabs), adding another polyA-selection step to remove contaminant RNA. Paired-end 2×150 bp RNA-seq was performed on an Illumina NovaSeq 6000 by Macrogen. RNA-seq reads of *Mastigamoeba abducta*, *Mastigella eilhardi* and *R. libera* were produced previously and are deposited in NCBI under BioProject PRJNA380424 [33].

### Transcriptome assembly

Raw reads of *Mastigamoeba abducta*, *R. vacuolata*, *R. libera* and *Endolimax* sp. were quality and adapter trimmed by BBDuk v36.92 (part of BBTools: https://jgi.doe.gov/data-and-tools/bbtools/). Reads of *Mastigella eilhardi* were trimmed by Trimmomatic v0.32 [34] using the settings SLIDINGWINDOW:10 : 25 MINLEN:50. Trimmed reads were assembled using Trinity r20140717 or v2.8.6 [35] under default parameters. Identities of each species were assessed by extracting small subunit rRNA (SSU rRNA) genes. This identified a single archamoebal SSU rRNA gene sequence in each assembly.

To remove possible contaminants, transcript sequences from all species were subjected to blast v2.11.0 [36] searches against the NCBI nucleotide database with an E-value cut-off 1e-02, collecting the five best hits, and filtered by an in-house script (https://github.com/kikinocka/ngs/blob/master/py_scripts/NT_filt.py) with an identity threshold of 75 % (-p 75) and a query coverage threshold of 50 % (-q 50), i.e. removing any sequences reliably matching targets not taxonomically assigned to Amoebozoa. Protein sequences were then predicted by TransDecoder (https://github.com/TransDecoder/TransDecoder) under default settings, and a second round of decontamination was performed. Protein sequences were screened against the NCBI non-redundant database using DIAMOND v0.9.31 [37] with an E-value cut-off 1e-05, collecting one hit, and filtered by an in-house script (https://github.com/kikinocka/ngs/blob/master/py_scripts/NR_filt.py) with identity threshold of 70 % (-p 70) to non-Amoebozoa targets. The completeness of all assemblies was evaluated by BUSCO v5 in protein mode with the eukaryota_odb10 dataset of conserved orthologues [38].

### Evaluation of MRO prediction tools

The performance of available predictors was evaluated on a set of *bona fide* MRO and cytosolic proteins. The set of cytosolic proteins was composed of homologues of *Entamoeba histolytica* ribosomal proteins (obtained from AmoebaDB [39]), and homologues of human and *Dictyostelium discoideum* replisomal proteins [40] identified in assemblies of analysed species by reciprocal best blast hit searches using AMOEBAE [41]. The set of MRO proteins was composed of all proteins previously identified as MRO-residing in *Entamoeba histolytica*, *Mastigamoeba balamuthi* and *P. schiedti* [10, 28–30, 42]. Subcellular localizations of these proteins were predicted by TargetP v2 [43], NommPred [44], MitoFates [45], MultiLoc2 [46], Predotar [47], PProwler [48], Cello [49] and DeepLoc v2.0 [50]. NommPred was used in the MRO setting, as all species possess a reduced mitochondrion, and in the *Dictyostelium* setting, as they all belong to Amoebozoa [33]. Since Archamoebae species do not possess a plastid, non-plant options were chosen for most predictors. DeepLoc v2.0 does not allow such a choice and assigned four MRO proteins as plastid-targeted. As plastidial and mitochondrial targeting signals share some common characteristics [51], we considered these predictions mitochondrial (marked as ‘PT→MT’ in Tables S2 and S3). Next, proteins were divided into the following categories: (i) true positives (TP) – proteins previously identified to reside in MROs for which mitochondrial targeting was predicted, (ii) true negatives (TN) – ribosomal and replisomal proteins for which mitochondrial targeting was not predicted, (iii) false positives (FP) – ribosomal and replisomal proteins for which mitochondrial targeting was predicted, and (iv) false negatives (FN) – MRO proteins for which mitochondrial targeting was not predicted. The sensitivity and specificity of each predictor were then calculated as TP/(TP+FN) and TP/(TP+FP), respectively.

### Prediction of MRO proteomes

Putative MRO proteomes were predicted from the protein sets by two methods: (i) homology searches using known mitochondrial and MRO proteins as queries, and (ii) MRO targeting prediction. The mitochondrial proteome of *Acanthamoeba castellanii*, predicted hydrogenosomal proteomes of *Mastigamoeba balamuthi* and *P. schiedti*, and the mitosomal proteome of *E. histolytica* served as queries in blast v2.8.1 searches against transcriptome-derived protein databases. HMMER v3.3 [52] searches using profiles composed of known homologues from a broad eukaryotic dataset (available on Figshare: https://figshare.com/projects/MRO-logy_of_Archamoebae_species/160169) were performed to identify components of the protein translocation machinery. Protein domains were predicted by InterProScan [53] implemented in Geneious Prime v2020.2.5 [54]. Proteins were clustered to orthologous groups using OrthoFinder v2.0.0 [55] under default settings.

### Phylogenetic analyses

The SSU rRNA gene of *Endolimax* sp. ZUBR was amplified from genomic DNA, isolated by using a ZR Genomic DNA II Kit (Zymo Research), using primers PeloSSU59F and PeloSSU750R as described previously [22], and was sequenced using an ABI PRISM 3100 sequencer (Applied Biosystems). The sequence was deposited in GenBank under accession number OQ546721. For SSU rRNA gene phylogenetic analysis, a data set was created that consisted of 64 SSU rRNA gene sequences, including already published [22–24] and newly determined (*Endolimax* sp. ZUBR) sequences of strains used in this study as well as sequences extracted from the newly generated transcriptome assemblies. The sequences were aligned by MAFFT [56] using the MAFFT 7 server (http://mafft.cbrc.jp/alignment/server/) with the G-INS-i algorithm and default settings. The alignment was manually edited using BioEdit v7.0.9.0 [57] in order to trim primer sequences and remove hypervariable positions. The length of the final data set was 1430 positions. A phylogenetic tree of SSU rDNA was estimated by the maximum-likelihood method with RAxML v8.0.0 [58] under the GTRGAMMAI model. Branch support was assessed by analysis of 1000 bootstrap pseudoreplicates.

Phylogenetic analyses were conducted for selected proteins using homologous sequences retrieved from NCBI and/or the EukProt database [59]. Sequences were aligned by MAFFT v7.458 [60] under the l-INS-i setting or Muscle v5 [61], and poorly aligned positions were removed by trimAl v1.4.rev15 [62] using -gt 0.8. Maximum-likelihood phylogenetic analyses were performed by RAxML v8.2.8 [58] using the LG4X model and the number of rapid bootstrap replicates (-f a) was determined by the program as necessary for obtaining stable support values (-N autoMRE_IGN). Bayesian inference was performed by MrBayes v3.2.7a [63] under a mixed amino acid model with four gamma rate categories, and at least 10 million Markov chain Monte Carlo generations. Sampling frequency was set to every 1000 generations with the first 25 % of the runs discarded as burn-in. Tree convergence was ensured when the average standard deviation of split frequency values fell below 0.01. Bootstrap support values from maximum-likelihood analysis were overlaid onto the MrBayes tree topology with posterior probabilities.

## Results

### Species and assemblies

**Fig. 1.**
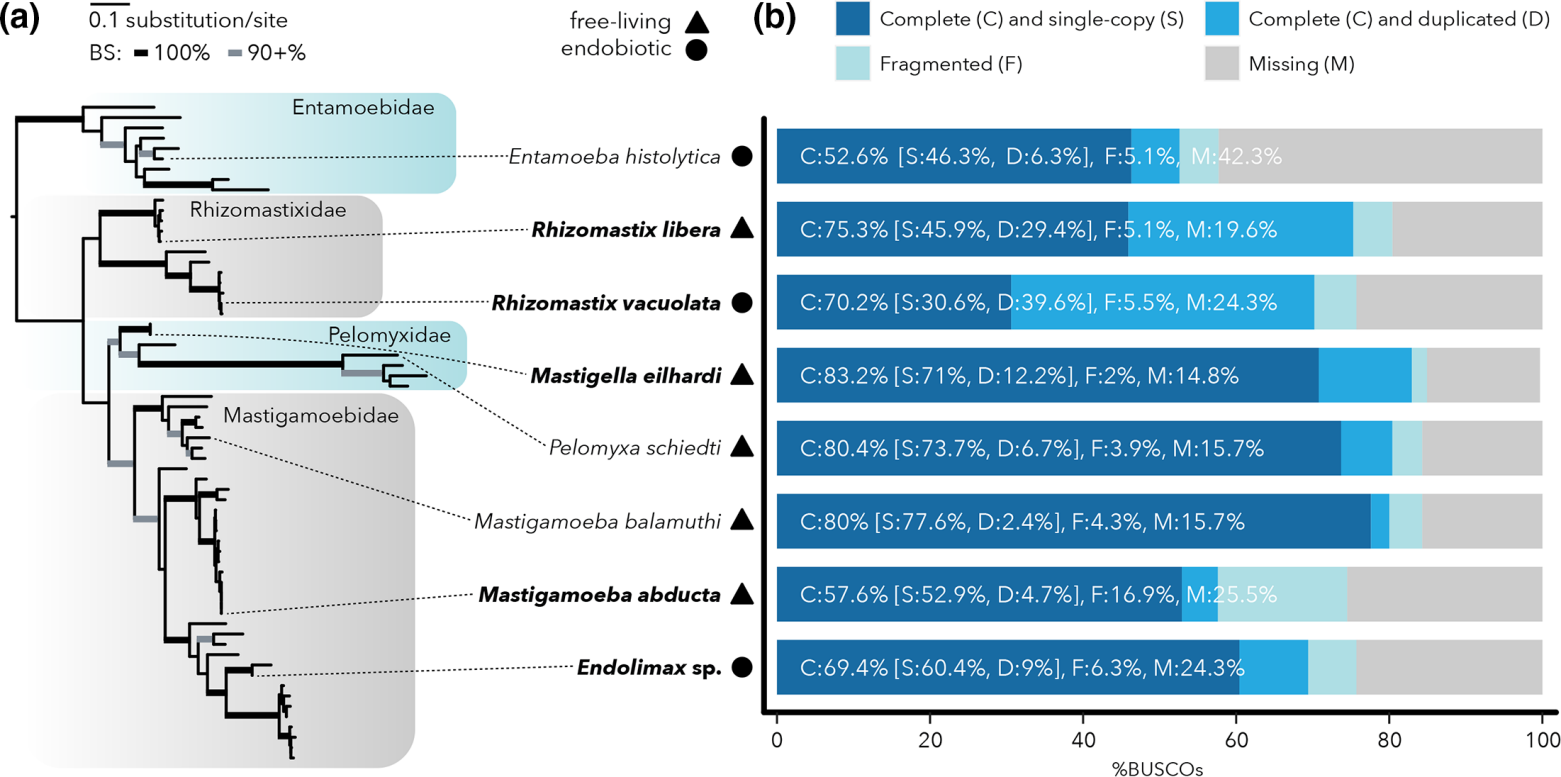
Archamoebae species investigated in this study. (**a**) SSU rRNA gene phylogeny with branches with full and high bootstrap support (BS) indicated as thicker black and grey lines, respectively (for the detailed tree see Fig. S1). Species lifestyle is marked by pictograms explained in the graphical key above. Species with new data produced within this study are shown in bold. (**b**) The completeness of transcriptome-derived protein datasets produced in this study was evaluated by BUSCO v5 using the odb10_eukaryota database and compared with the completeness of genome-derived protein datasets from *Entamoeba histolytica*, *Mastigamoeba balamuthi* and *P. schiedti*.

We compared metabolic pathways putatively localized to MROs of eight Archamoebae species, including three species investigated previously (*Entamoeba histolytica* [10, 42], *Mastigamoeba balamuthi* [28, 29] and *P. schiedti* [30]) and five species, whose transcriptomes were assembled here (*R. libera*, *R. vacuolata, Mastigella eilhardi*, *Mastigamoeba abducta*, and *Endolimax* sp. ZUBR). The sampling captures different lifestyles and comprises representatives of all major Archamoebae lineages (Figs 1a and S1, available in the online version of this article). The placement of the Rhizomastixidae clade is conflicting in phylogenomic analyses [33, 64, 65]. In one [33], Rhizomastixidae forms a sister lineage to Pelomyxidae, but maximum-likelihood analysis of the same dataset preferred the alternative, in which Pelomyxidae and Mastigamoebidae are sisters. The latter was recovered in other studies [64, 65] and in our SSU rDNA phylogeny (Fig. S1), and will serve as the framework for our evolutionary inferences.

The counts of predicted proteins for the species span between ~8000 in parasitic *Entamoeba histolytica* to >47 000 in free-living *Mastigella eilhardi* (Table S1). The completeness of all predicted proteomes was assessed using BUSCO analysis (Fig. 1b; Table S1). The percentage of missing genes varied from 14.8 % (*Mastigella eilhardi*) to 42.3 % (*Entamoeba histolytica*). In the genome-derived protein datasets of *Mastigamoeba balamuthi* and *P. schiedti*, missing genes represented only 15.7 % (both species). *Rhizomastix* spp. transcriptomes contain a considerable number of isoforms, and their removal resulted in a lower percentage of BUSCO duplicates but at the same time in a higher percentage of missing BUSCO markers (Fig. S2; Table S1). As noted previously [30, 40], the above proportion of missing BUSCO marker genes is not uncommon in non-model organisms, as they may diverge beyond recognition or become lost. The latter scenario is illustrated by the high-quality assembly of parasitic *Entamoeba histolytica* which lacks the highest number (42.3%) of BUSCO markers (Table S1). Regardless, absences of genes should be interpreted with caution as all datasets generated in this study are transcriptomes, and therefore lack genes that were not transcribed at the time of RNA isolation.

### Evaluation of MRO prediction tools

Protein translocation across the mitochondrial membranes is to some extent facilitated by membrane potential generated by the electron transport chain. N-terminal targeting sequences (NTS) of mitochondrial matrix proteins are therefore positively charged [66]. In organisms with reduced mitochondria that have been investigated, the membrane potential across the MRO inner membrane is reduced or absent [66–69]. Thus, localization prediction tools, trained on model organisms bearing canonical mitochondria, may not be reliable for organisms with MROs. For this reason, we tested several prediction tools, three of them in two different settings (see Methods and Table S2). The performance of MRO targeting prediction tools was evaluated on test sets of *bona fide* cytosolic and MRO proteins of Archamoebae (see Methods) and the sensitivity and specificity of each tool were calculated.

**Fig. 2.**
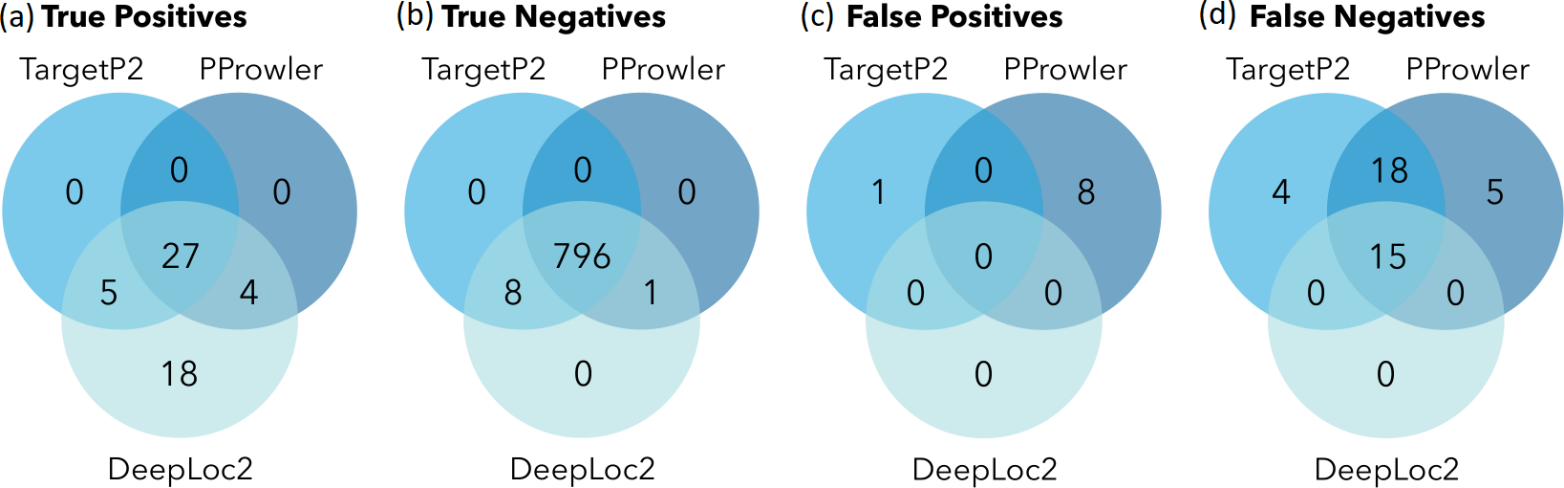
Evaluation of localization prediction tools. Localizations were predicted for proteins residing outside MROs (ribosomal and replisomal proteins) and previously identified MRO proteins [10, 28–30, 42]. Venn diagrams depict the overlaps between reference sequences truly and falsely identified as mitochondrial (i.e. positives) or non-mitochondrial (i.e. negatives) by the three best-scoring targeting predictors (Table S2).

Calculated specificity was below 35 % (i.e. >65 % of MRO predicted proteins were false positives) for all tools, except for TargetP2, PProwler and DeepLoc2, which reached 97, 79 and 100%, respectively (Table S2). Sensitivity ranged between 36 and 77 %, and while TargetP2 and PProwler showed mediocre values (46 and 45 %, respectively), DeepLoc2 reached the highest (77 %; Table S2). We then visualized how the inferred localization of individual reference proteins differed among these three highest-performing predictors (Fig. 2). Most true positives (27 MRO references identified as MRO localized) and true negatives (796 non-MRO references identified as non-MRO) were predicted by all three tools (Fig. 2a, b). Among false positives (non-MRO references identified as MRO targeted), most were assigned by PProwler, which had eight false positive identifications; TargetP2 and DeepLoc2 identified one and zero false positives, respectively (Fig. 2c). A comparable number of MRO references identified as non-MRO (false negatives) was assigned by TargetP2 and PProwler (37 and 38, respectively), while DeepLoc2 missed only 15 proteins (Fig. 2d). Combined predictions from all three tools gave robust results for the testing datasets (ribosomal, replisomal and previously identified MRO proteins), and therefore these tools were chosen to predict the localization of putative MRO proteins in the studied Archamoebae (Table S3).

### MRO proteome prediction

Proteins were classified as putative MRO proteins if they: (i) were predicted as mitochondrion-localized by at least one of the best-performing prediction tools (TargetP2, PProwler and DeepLoc2), or (ii) showed phylogenetic affinity with a previously characterized MRO protein. The compositions of predicted MRO proteomes are summarized in Table S3 and are discussed below by functional category.

### Protein import and processing

**Fig. 3.**
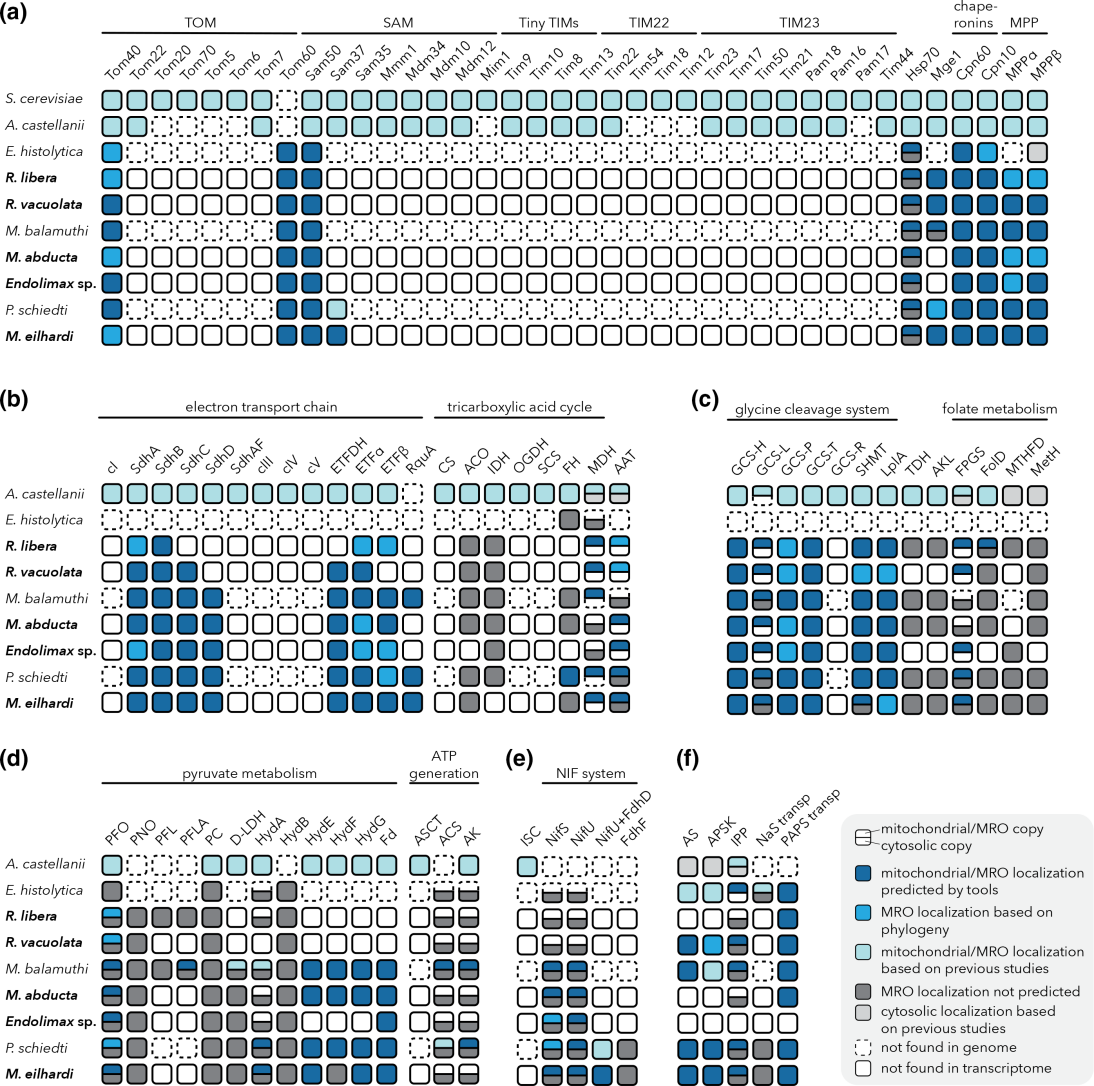
Mitochondrial pathways in Archamoebae species. Newly studied species are shown in bold. Presence/absence of components (explained in the graphical key) was compared with those of *A. castellanii* and/or yeast mitochondria. (**a**) Protein import machinery. TOM/TIM, translocase of the outer/inner membrane; SAM, sorting and assembly machinery; Pam, presequence translocase-assisted motor; Hsp70, heat shock protein 70; Mge1, nucleotide exchange factor; Cpn60/10, chaperonin 60/10; MPPα/β, mitochondrial processing peptidase α/β subunit. (**b**) Electron transfer chain and tricarboxylic acid cycle. cI, complex I; cIII–cV, complex III–V; SdhA–D, succinate dehydrogenase subunit A–D; SdhAF, succinate dehydrogenase assembly factor; ETFDH, electron transferring flavoprotein dehydrogenase; ETFα/β, electron transferring flavoprotein subunit α/β; RquA, rhodoquinone methyltransferase; CS, citrate synthase; ACO, aconitase; IDH, isocitrate dehydrogenase; OGDH, 2-oxoglutarate dehydrogenase; SCS, succinyl-coenzyme A synthetase; FH, fumarase; MDH, malate dehydrogenase; AAT, aspartate aminotransferase. (**c**) Glycine cleavage system and folate metabolism. GCS-H/L/P/T/R, glycine cleavage system H/L/P/T/R protein; LplA, lipoamide protein ligase; SHMT, serine hydroxymethyltransferase; TDH, threonine dehydrogenase; AKL, α-amino-β-ketobutyrate coenzyme A ligase; FPGS, folylpolyglutamate synthase; FolD, tetrahydrofolate dehydrogenase/cyclohydrolase; MTHFD, methylenetetrahydrofolate dehydrogenase; MetH, B_12_-dependent methionine synthase. (**d**) Pyruvate metabolism and ATP generation. PFO, pyruvate:ferredoxin oxidoreductase; PNO, pyruvate:NADP^+^ oxidoreductase; PFL, pyruvate:formate lyase; PFLA, pyruvate:formate lyase activating enzyme; PC, pyruvate carboxylase; d-LDH, d-lactate dehydrogenase; HydA, [FeFe]-hydrogenase group A; HydB, [FeFe]-hydrogenase group B; HydE/F/G, hydrogenase maturases; Fd, ferredoxin; ASCT, acetate:succinate coenzyme A-transferase; ACS, acetyl-coenzyme A synthetase; AK, adenylate kinase. (**e**) Iron–sulphur cluster assembly. ISC, iron–sulphur cluster; NIF, nitrogen fixation; NifS, cysteine desulfurase; NifU, scaffold protein; FdhD, formate dehydrogenase accessory sulfurtransferase; FdhF, formate dehydrogenase. (**f**) Sulphate activation pathway. AS, ATP sulfurylase; APSK, adenosine-5′-phosphosulphate kinase; IPP, inorganic pyrophosphatase; NaS transp, sodium sulphate transporter; PAPS transp, 3′-phosphoadenosine 5′-phosphosulfate transporter.

The protein import machineries of Archamoebae (Fig. S3) contain very few subunits known from opisthokonts or *A. castellanii* [1, 30, 42]. Therefore, they are either minimalistic, composed of components non-homologous to those in model systems, or their sequences may be too divergent from those of opisthokont and *A. castellanii* proteins to be detectable using our methods. Consistently, our searches identified only four components of the translocase of the outer membrane (TOM) and the sorting and assembly machinery (SAM) (Fig. 3a; Table S3). Tom40 and Sam50, central β-barrel pores involved in protein translocation and insertion into the outer membrane, respectively [70], and Tom60, an Archamoebae-specific shuttle receptor [30], were ubiquitously present in all studied species. Sam37 was found only in *Mastigella eilhardi* and *P. schiedti* suggesting that it was retained in Pelomyxidae species, and independently lost in other lineages. No subunits of the translocase of the inner membrane (TIM) were detected, leaving the question of the nature of the import system across the inner MRO membrane open.

The mitochondrial heat shock protein 70 (mtHsp70) is involved in pulling the translocated proteins stepwise into the mitochondrial matrix [70]. This process is ATP-dependent and aided by the nucleotide exchange factor Mge1 [71, 72]. Both proteins were identified in Archamoebae species, except for Mge1 missing in *Entamoeba histolytica*, *Mastigamoeba abducta* and *Endolimax* sp. (Fig. 3a; Table S3). After protein translocation, the NTS must be cleaved off by the matrix processing peptidase (MPP) to ensure proper folding of the protein, which is in turn facilitated by chaperonins Cpn60 and Cpn10. Cpn60, Cpn10 and both subunits of MPP were found in all species except for *Entamoeba histolytica*, which only possesses MPPβ (Fig. 3a; Table S3).

### Electron transport chain and tricarboxylic acid cycle

In aerobic mitochondria, the electron transport chain (ETC) is responsible for the reoxidation of reduced cofactors accumulated by catabolic processes, such as the TCA cycle or fatty acid oxidation, and for the transfer of electrons to their final acceptor, oxygen, in a process known as respiration. This electron flow generates membrane potential, which is harvested by F_0_F_1_ ATPase to produce ATP, and thus completes the entire process of oxidative phosphorylation. The set of ETC complexes and TCA enzymes is quite reduced in anaerobic MROs (Fig. S3). While these proteins are completely missing in the mitosome of *Entamoeba histolytica*, succinate dehydrogenase (SDH/complex II/cII) and malate dehydrogenase (MDH) were found in the hydrogenosomes of *Mastigamoeba balamuthi* and *P. schiedti* [29, 30]. Moreover, fumarate hydratase (fumarase/FH, class I) is putatively targeted to the hydrogenosomes of *P. schiedti*, thus potentially providing fumarate as an acceptor for electrons produced by SDH and yielding succinate as the end product [30]. This process of fumarate respiration, in which the TCA reactions run in a reductive direction, is known from several anaerobic mitochondria and MROs [7]. ETC complexes I and III–V were lost in all Archamoebae, but we found four SDH subunits (A–D) in the studied species, except for SdhC and SdhD in *R. libera* and SdhD in *R. vacuolata* (Fig. 3b; Table S3). The assembly factor (SdhAF/Sdh5) necessary for attaching a flavin moiety to SdhA in *A. castellanii* [1] seems to be genuinely missing from all Archamoebae (Table S3).

The reductive direction of the TCA enzymes’ activities is supported by the presence of the electron-transferring flavoprotein (ETF) complex and the methyltransferase RquA in some species. RquA is involved in the synthesis of rhodoquinone (RQ), a quinone with lower electron potential than ubiquinone. Electrons from its reduced form, rhodoquinol, may pass to fumarate under the catalysis of SDH [73, 74]. We speculate that due to the absence of complex I, rhodoquinol is regenerated by electrons transferred from the ETF complex. The ETF complex consists of the membrane-bound ETF dehydrogenase (ETFDH) and two soluble subunits, alpha and beta (ETFα and ETFβ). The whole complex was found in all species, except for *Entamoeba histolytica* and missing ETFDH in *R. libera* and ETFβ in *R. vacuolata* (Fig. 3b; Table S3). However, the distribution of RquA is patchy, as it was identified only in *Mastigella eilhardi* (Fig. 3b; Table S3) and previously in *P. schiedti* and *Mastigamoeba balamuthi* [30, 73], so the universality of this pathway in Archamoebae could not be ascertained.

Several other TCA cycle enzymes were found, but only MDH was predicted to function within MROs in most of the investigated species (Fig. 3b; Table S3). Oxaloacetate, the substrate of MDH, can be produced by aspartate aminotransferase (AAT) from 2-oxoglutarate putatively transported from the cytosol (see below). AAT was indeed identified in all species (Table S3), but only in Pelomyxidae were AAT and MDH simultaneously predicted to localize in the MRO (Fig. 3b). The sequences from *Rhizomastix* spp. branch with the putatively MRO-targeted isoforms (Fig. S4A), but clearly lack NTS (Table S3).

### Glycine cleavage system and folate metabolism

Most mitochondria take part in the metabolism of amino acids. In many MROs of free-living protists, the glycine cleavage system (GCS) is retained [75, 76]. This is a major route of glycine catabolism, whereby glycine is decomposed into CO_2_ and ammonia with an associated transfer of a methyl group into the one-carbon pool or used by serine hydroxymethyltransferase (SHMT) for serine biosynthesis [77, 78]. In these reactions, tetrahydrofolate (THF) acts as a methyl acceptor to yield N5,N10-methylenetetrahydrofolate (CH_2_-THF) (Fig. S3). Glycine could be produced from threonine by threonine dehydrogenase (TDH) and α-amino-β-ketobutyrate coenzyme A (CoA) ligase (AKL) [79, 80]. However, these enzymes are probably cytosolic in *Mastigamoeba balamuthi* and *P. schiedti* [29, 30], suggesting that glycine is probably imported from the cytosol.

GCS consists of four subunits (H-, L-, T- and P-protein). GCS-H requires the attachment of a lipoamide residue that is delivered by lipoamide protein ligase (LplA). We identified all core GCS proteins and LplA in the studied species as MRO-targeted (Fig. 3c; Table S3), except for a regulatory protein, GCS-R [1], which seems absent in Archamoebae. SHMT was found in two copies in *R. vacuolata* and *Mastigella eilhardi* (Table S3). While not all these proteins were predicted as mitochondrion-targeted (some of GCS-P, SHMT and LplA sequences), they formed robust clades in phylogenies (Fig. S4B-D). We therefore predict their involvement in MRO metabolism (Fig. 3c). The exception is the second copy of L-protein (GCS-L2) already known from *Mastigamoeba balamuthi* and *P. schiedti* and here identified in *Mastigella eilhardi* (Fig. S4E), which is putatively cytosolic and has an unclear function [29, 30].

Two products of the GCS, NADH+H^+^ and CH_2_-THF, are worth further consideration. NADH+H^+^ needs to be reoxidized, for which MDH is the most likely candidate (see above). CH_2_-THF is a one-carbon-transferring cofactor contributing to THF- and S-adenosylmethionine (SAM)-dependent methylation reactions, including RNA and DNA methylation, nucleotide and methionine synthesis and secondary metabolism; these processes mostly reside in the cytosol [81, 82]. THF- and SAM-dependent methyl pools are linked via the cytosolic methionine cycle, involving methionine synthase, SAM synthetase, methyltransferase and adenosylhomocysteinase. Consistently, B_12_-dependent methionine synthase (MetH) is present in all species except *Entamoeoba histolytica* and is presumably cytosolic (Fig. 3c; Table S3). Recently, the contribution of MRO to the cytosolic one-carbon pool was highlighted in the anaerobic metamonad *Paratrimastix pyriformis* [17]. There, both the MRO and the cytosol house enzymes interconverting CH_2_-THF and formate, i.e. THF dehydrogenase/cyclohydrolase (FolD) and CH_2_-THF dehydrogenase (MTHFD), and probably exchange these metabolites. Importantly, THF cofactors can be compartmentalized (prevented from diffusing) through polyglutamylation by folylpolyglutamate synthase (FPGS) [83]. These enzymes were present in most studied Archamoebae, but none of them had an NTS except for one copy of FolD in *R. libera* and FPGS in all species except Mastigamoebidae (note that Pelomyxidae possess two copies of the enzyme, while all other Archamoebae possess only one) (Fig. 3c; Table S3). This suggests that polyglutamylation of THF in most species takes place in MRO, but enzymes interconverting CH_2_-THF and formate are restricted to the cytosol. One-carbon units are probably transported from the MRO as CH_2_-THF, the product of reactions catalysed by GCS or SHMT.

### Pyruvate metabolism and ATP generation

Pyruvate is central to many metabolic pathways and in aerobic catabolism it is typically decarboxylated by the mitochondrial pyruvate dehydrogenase complex to acetyl-CoA to feed the TCA cycle. In anaerobic derivatives of mitochondria, pyruvate is converted to acetyl-CoA by oxygen-sensitive enzymes (Fig. S3), such as pyruvate:ferredoxin oxidoreductase (PFO), pyruvate:NADP^+^ oxidoreductase (PNO) or pyruvate:formate lyase (PFL). *Entamoeba histolytica* bears only cytosolic PFO, but *Mastigamoeba balamuthi* and probably also *P. schiedti* possess both hydrogenosomal and cytosolic PFO, in addition to cytosolic PNO [29, 30]. PFL has been only found in the cytosol of *Mastigamoeba balamuthi* [29], whereas in *P. schiedti*, pyruvate carboxylase (PC), carboxylating pyruvate to oxaloacetate, was weakly predicted to localize in the MRO [30]. In all studied Archamoebae, we only identified cytosolic forms of PNO and PFL, if present (Fig. 3d; Table S3). PC was also predicted to be cytosolic in all species including *P. schiedti* (Table S3). For PFO, we found evidence for both cytosolic and MRO copies in all species with the exception of *Entamoeba histolytica*. In *Mastigamoeba abducta*, *Endolimax* sp. and *Mastigella eilhardi*, the putative MRO copy had a predicted NTS. In both *Rhizomastix* species, the N-termini were probably incomplete, missing the starting methionine (Fig. S5A) and thus a potential NTS, but in both cases they cluster with MRO copies in the phylogenetic tree (Fig. S4F, Table S3). Pyruvate can also be interconverted with lactate by lactate dehydrogenase. This enzyme was previously found to be cytosol-localized in *Mastigamoeba balamuthi* and *P. schiedti* with an additional MRO-localizing copy in *Mastigamoeba balamuthi* [29, 30]. In other studied Archamoebae, we identified only a cytosolic copy (Fig. 3d; Table S3). Phylogenetic analysis showed that the MRO-targeted version arose by duplication specific for *Mastigamoeba balamuthi* (Fig. S4G). In other species, pyruvate is probably imported via an unidentified pyruvate carrier (Table S3).

In many MROs, electrons produced by the oxidation of pyruvate can be transferred via ferredoxin (Fd) to [FeFe]-hydrogenase producing molecular hydrogen (Fig. S3). [FeFe]-hydrogenases form four distinct phylogenetic groups (HydA, -B, -C and -D). HydA requires maturation by hydrogenase maturases (HydE, -F and -G) [84], and this is the type that putatively produces hydrogen in the MROs of free-living species *Mastigamoeba balamuthi*, *Mastigamoeba abducta*, *P. schiedti* and *Mastigella eilhardi* (Fig. 3d; Fig. S4H; Table S3) [29, 30]. Although HydA was not predicted to localize in MROs of *Mastigamoeba abducta*, the presence of maturases and ferredoxin in its MRO strongly suggests the presence of HydA in the same compartment. HydF from *Mastigella eilhardi* was incomplete at its N-terminus (Fig. S5B), which precluded predicting its localization (Fig. 3d; Table S3). Notably, both *Rhizomastix* spp. uniformly lacked all these proteins in their MRO (Fig. 3d; Table S3), as did *Endolimax* sp. and parasitic *Entamoeba histolytica*. This suggests that pyruvate metabolism has been independently lost from the MRO at least twice in the Archamoebae. The situation is somewhat more complicated for *Endolimax* sp. It possesses MRO-targeted PFO and Fd, but no HydA and its maturases were found (Fig. 3d; Table S3). Therefore, the fate of electrons produced by PFO-mediated pyruvate oxidation is unclear in this species. All Archamoebae encoded a probable cytosolic hydrogenase homologue HydB.

Acetyl-CoA produced by pyruvate decarboxylation can be converted to acetate by acetyl-CoA synthetase (ACS), with concomitant CoA release and ATP production (Fig. S3). Alternatively, this can be achieved by the combined action of acetate:succinate CoA-transferase (ASCT) and succinyl-CoA synthetase (SCS) [7]. ATP may also be generated by adenylate kinase (AK) by the interconversion of adenine nucleotides (Fig. S3). All Archamoebae species lacked ASCT and SCS (Fig. 3b, d; Table S3). To our surprise, none of the identified ACS and AK had a predicted NTS, except for one *Mastigamoeba balamuthi* ACS and AK, and possibly three *P. schiedti* AKs. The reported MRO targeting of *P. schiedti* ACS [29, 30] was not confirmed here by validated predictors (Table S3). The phylogenies are inconclusive, i.e. both MRO-targeted and cytosolic ACS copies of *Mastigamoeba balamuthi* reside in one clade (Fig. S4I). Similarly, *P. schiedti* MRO-targeted and cytosolic AKs cluster in one clade, and the *Mastigamoeba balamuthi* MRO copy forms a separate lineage (Fig. S4J). Therefore, Archamoebae are unlikely to synthesize ATP by MRO-localized AK and/or ACS except for *Mastigamoeba balamuthi* and *P. schiedti*.

### Iron–sulphur cluster assembly

Iron–sulphur (Fe-S) clusters are cofactors necessary for a proper function of dozens of essential proteins and there are three dedicated systems in eukaryotes that ensure their formation and insertion into proteins. In a textbook plastid-bearing eukaryotic cell (e.g. *Arabidopsis thaliana*) the cytosolic iron–sulphur cluster assembly (CIA), the mitochondrial iron–sulphur cluster pathway (ISC) and the plastid sulphur mobilization pathway (SUF) are in place [85, 86]. Although CIA and ISC operate in *A. castellanii* [1], Archamoebae have replaced the latter by the minimal Fe-S (MIS) system [87] of NIF (nitrogen fixation) type via horizontal gene transfer from bacteria [28, 30]. The system (Fig. S3), composed of the scaffold protein NifU and cysteine desulfurase NifS, is duplicated in *Mastigamoeba balamuthi* and localized in both the cytosol and the hydrogenosome, while in *Entamoeba histolytica* one copy is present, operating probably in the cytosol [28, 29]. In addition to the cytosolic and putative MRO copies of NifS and NifU in *P. schiedti*, there are four additional NifS copies with unknown localization and a fused protein containing the N-terminal domain of NifU and the C-terminal domain of formate dehydrogenase accessory sulfurtransferase (FdhD) [30]. This NifU+FdhD is probably MRO-targeted and in theory may play a role in the activation of formate dehydrogenase (FdhF), which is present but without evidence for its MRO localization [30].

The five additional Archamoebae examined here contain only the NIF pathway (Fig. 3e; Table S3). Notably, both *Rhizomastix* species possessed only one copy of each NIF protein, which fell outside the mitochondrial clades in phylogenies (Fig. S4K-L), resembling the situation in *Entamoeba histolytica*. Similarly to *Mastigamoeba balamuthi*, *Mastigamoeba abducta* and *Endolimax* sp. contained two copies of both NIF proteins, which were clearly separated to cytosolic and mitochondrial homologues in the tree (Fig. S4K-L), with *Endolimax* sp. NifS not predicted as mitochondrial (Table S3). Finally, *Mastigella eilhardi*, like *P. schiedti*, contained besides cytosolic and MRO copies of both NifS and NifU, a third copy of a putatively cytosolic NifS and a fusion protein NifU+FdhD predicted to reside in the MRO (Fig. 3e; Table S3). Clearly, the composition of the NIF system in Archamoebae varies but is conserved within clades.

The absence of Fe-S cluster assembly in the *Entamoeba histolytica* mitosome is plausible, as there are no known client proteins for Fe-S clusters in the compartment. However, the presence of Fe-S proteins, SdhB and possibly PFO, in *Rhizomastix* spp. MROs requires their assembly directly in the organelle. This may be theoretically achieved by dual localization of the single copy of the NIF system to both compartments as was previously speculated for *Entamoeba histolytica* [88]. The localization of two CIA components, Cia2 and Nbp35, in the mitosome of *Giardia intestinalis* [89] also hints to the fact that such dual localization of Fe-S cluster assembly proteins might be achieved without a recognizable NTS.

### Sulphate activation pathway

The sulphate activation pathway is the only experimentally verified pathway in the mitosomes of *Entamoeba histolytica* [27]. The final product, PAPS, is produced by the activities of ATP sulfurylase (AS) and adenosine-5′-phosphosulfate kinase (APSK) from sulphate, imported via a sodium/sulphate (NaS) symporter, and ATP (Fig. S3). Pyrophosphate produced in the first reaction is hydrolysed to phosphate by inorganic pyrophosphatase (IPP). PAPS is then exported to the cytosol in exchange for ATP via a PAPS transporter belonging to the mitochondrial carrier family (MCF) proteins. In the cytosol, it serves as a sulphur donor for the synthesis of sulpholipids necessary for the parasite’s proliferation and encystation [26, 27, 90]. The sulphate activation pathway was found also in the hydrogenosomes of *Mastigamoeba balamuthi* and *P. schiedti*, although the identity of the MRO-localized NaS symporter could not be ascertained [29, 30]. We identified the pathway in *Mastigella eilhardi* and *R. vacuolata* (Figs 3f and S4M,N; Table S3); in the latter species APSK did not possess an NTS but clustered together with other MRO-targeted APSKs (Fig. S4M). Notably, the pathway was completely missing in *Endolimax* sp., *Mastigamoeba abducta* and *R. libera* (Fig. 3f; Table S3). Presence of the PAPS transporter by itself is not significant as it may transport other adenosine moieties, and represent a means of ATP import into their MROs [91].

### Transporters

MCF transporters transfer small molecules between mitochondria and cytosol. To gain insights into which moieties could be transferred in Archamoebae, we searched in their datasets for 53 MCF transporters previously identified in human [92] and placed them into the phylogenetic tree including transporters from yeast, *Arabidopsis thaliana*, *Acanthamoeba castellanii* and *Dictyostelium discoideum* [1, 93, 94]. Not surprisingly, the number of transporters was significantly smaller (Fig. S4O; Table S3). The highly divergent phosphate transporter of *Entamoeba histolytica* [42] was not identified in any other Archamoebae. All except *Endolimax* sp. possess PAPS transporters (see above), and this was the only identified transporter in *Rhizomastix* spp. All Mastigamoebidae species possess a transporter from a clade of ATP, NAD, FAD/folate and pyrimidine transporters, but without a close affinity to any of them. In addition, *Endolimax* sp. encodes a GTP/GDP carrier.

The broadest inventory of transporters was found in Pelomyxidae which besides PAPS transporter encode four more types of carriers (Fig. S4O). The first is a clear homologue of pyrimidine transporter. The second is affiliated to carnitine, ornithine and amino acid transporters (including aspartate/glutamate antiporters) and NAD transporters but without statistical support. The third branched together with citrate (CIC) transporters inside a larger clade of oxodicarboxylic acid transporters (topology unsupported in maximum-likelihood analysis). The last showed affinity to 2-oxoglutarate/malate antiporters. The combination of putative aspartate/glutamate and 2-oxoglutarate/malate antiporters recalls the malate–aspartate shuttle known from textbook mitochondria. If acting in reverse, this system would provide oxaloacetate for MDH and remove the reduced malate, thus maintaining the redox balance of the MRO. However, it requires also cytosolic activity of AAT and MDH, which in the case of MDH is not supported by our data. Regardless, the inventory of carriers in Pelomyxidae suggests richer biochemical potentials of their MROs compared to other Archamoebae, the functions of which may relate to the presence of reversed partial TCA cycle or redox balance maintenance.

### Lipid transport between MRO and endoplasmic reticulum

To identify additional MRO proteins that were not queried by targeted searches described above, all predicted proteins were clustered into orthologous groups (OGs) and their predicted subcellular targeting was integrated with this clustering. We reasoned that true positives would cluster, and so we should see OGs enriched with proteins predicted to be MRO-localized. By this approach we identified the mitochondrial Rho GTPase (Miro) and vacuolar sorting protein 13 (Vps13). Miro is involved in calcium-dependent regulation of mitochondrial transport and maintenance of mitochondrial morphology and homeostasis [95]. It bears a C-terminal transmembrane domain (TMD) that confers its insertion and anchors it in the mitochondrial membrane [96]. The protein identity of found archamoebal homologues was confirmed by reverse blast searches into human and yeast protein databases (Table S4). Sequences with a predicted C-terminal TMD, i.e. from Mastigamoebidae and Pelomixidae (Table S3), were included into a dataset taken from previous studies [97–101] and formed a supported clade (Fig. S6).

Vps13 was found in all species including *Entamoeba histolytica* and predicted as MRO-localized only in three of 42 cases (Table S2). In fact, these three might represent false positives, as Vps13 belongs to a family of lipid transporters that bridge membranes to build a channel for lipid transport between organelles and is not expected to bear NTS [102]. One of its paralogues bridges the endoplasmic reticulum to mitochondria and peroxisomes where it is recruited by Miro [103]. The presence of several Vps13 paralogues and Miro in Archamoebae and *Acanthamoeba castellanii* (Table S4) indicate that lipid transport is essential irrespective of the functional or metabolic state of the mitochondrial organelle (mitochondrion vs. hydrogenosome vs. mitosome).

## Discussion

**Fig. 4.**
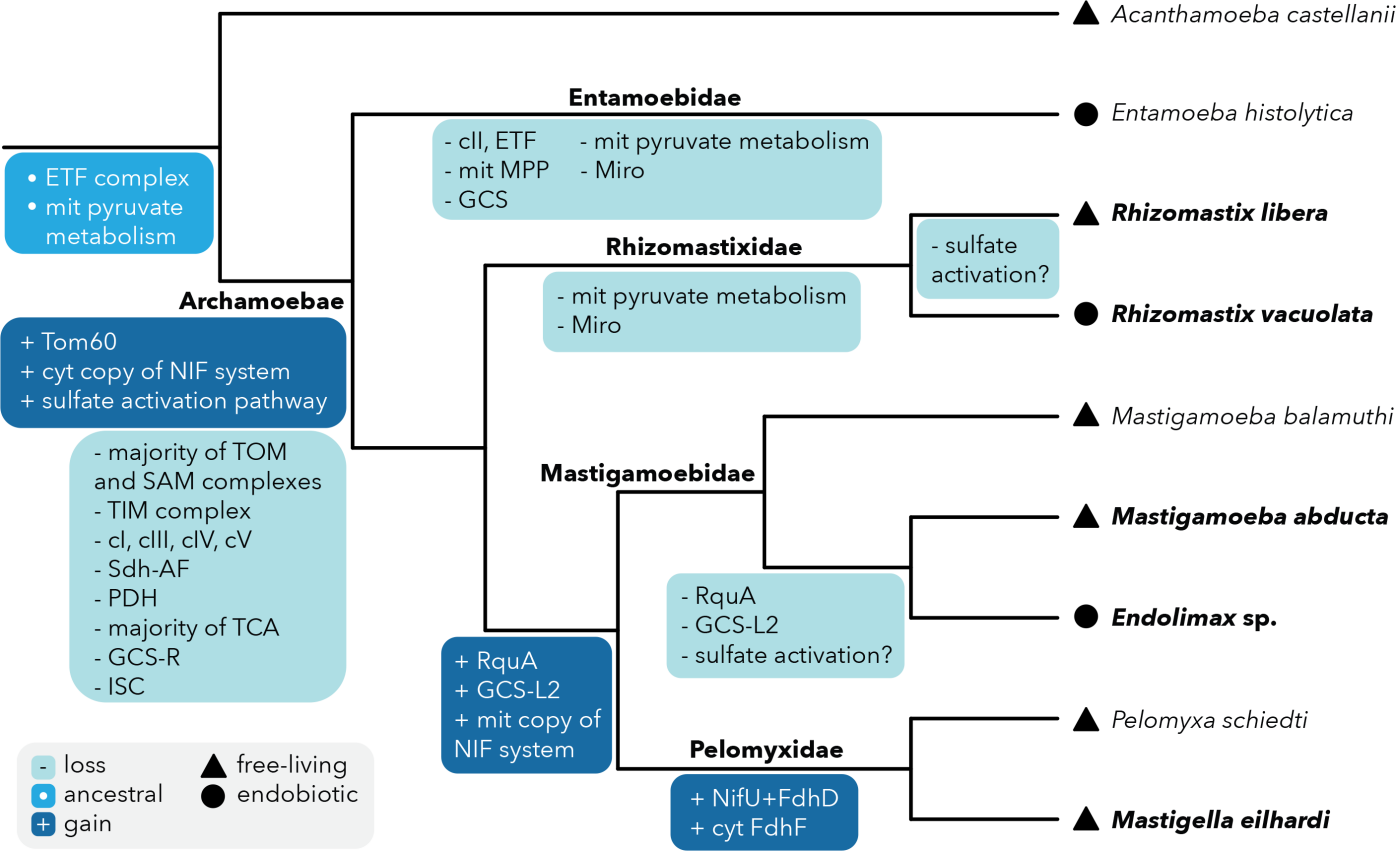
The evolutionary history of MRO-localized pathways in Archamoebae. Ancestral, gained and lost enzymes and metabolic pathways are shown by different shades of blue on a schematic phylogenetic tree. Lifestyles are shown for all included species by a different symbol as explained in the graphical legend. Species with new data produced within this study are in bold type. Main clades of Archamoebae are shown at respective branches.

Altogether, our results suggest that the common ancestor of Archamoebae contained a highly reduced and modified MRO (Fig. 4), which is consistent with the exclusive occurrence of these species in anoxic or microaerobic environments. This MRO had already lost many features typical for aerobic mitochondria, including most subunits of the TOM/TIM translocons, respiratory chain complexes I and III–V, oxygen-dependent pyruvate dehydrogenase, most TCA cycle enzymes and the ISC-type Fe-S cluster assembly system. On the other hand, it had acquired multiple functions unobserved in other representatives of Amoebozoa, including a Tom60 receptor in the outer membrane translocon, sulphate activation pathway and MIS-type of the prokaryotic Fe-S cluster assembly.

Since the common ancestor of Archamoebae, MRO metabolism underwent lineage-specific modifications by functional losses and acquisitions (Fig. 4). The evolution of MRO functions did not follow a strictly habitat-driven path (compare free-living and endobiotic species); rather, there is a distinction among major lineages. Entamoebidae and Rhizomastixidae underwent further reductions of pyruvate metabolism in their MROs and potentially a loss or simplification of complex II and the ETF, with an extreme reduction in the parasitic *Entamoeba histolytica*. Hydrogenases were also lost, suggesting ATP production is no longer maintained in these MROs. Meanwhile, in Pelomyxidae and Mastigamoebidae, the MROs remained active in ATP and hydrogen production with the help of fumarate respiration probably connected with the rhodoquinone-type electron transfer between ETF and SDH complexes on the inner membrane. These MROs also acquired a dedicated NIF-like Fe-S cluster assembly via duplication of the cytosolic system and, specifically in Pelomyxidae, a third branch derived from the NIF system to possibly serve formate dehydrogenase activation. Catabolic functions performed by GCS and SDH appear widely conserved across Archamoebae, the former possibly coupled to AAT and MDH activity required for NADH reoxidation. GCS and SHMT probably supply one-carbon units to the cytosolic pool by folate species exchange. The occurrence of the sulphate activation pathway is patchy, with possible losses observed in unrelated lineages of *R. libera*, *Endolimax* sp. and *Mastigamoeba abducta*, and its importance for various Archamoebae remains to be clarified.

## Supplementary Data

Supplementary material 1

Supplementary material 2
