# Supplementary material for: Comparative analysis of mitochondrion-related organelles in anaerobic amoebozoans
Source: Microb Genom. 2023 Nov 23;9(11):001143. doi: 10.1099/mgen.0.001143 (PMC10711303; doi:10.1099/mgen.0.001143)
Supplement: Supplementary material 1 [file mgen-9-1143-s001.pdf]

Comparative analysis of mitochondrion-related organelles in anaerobic amoebozoans

Kristína Záhonová[1,2,3,4,*,#], Zoltán Füssy[1,*], Courtney W. Stairs[5,6], Michelle M. Leger[5,7], Jan Tachezy[1], Ivan Čepička[8], Andrew J. Roger[5], Vladimír Hampl[1,#]

[1] Department of Parasitology, Faculty of Science, Charles University, BIOCEV, Vestec, Czech Republic

[2] Institute of Parasitology, Biology Centre, Czech Academy of Sciences, České Budějovice (Budweis), Czech Republic

[3] Life Science Research Centre, Department of Biology and Ecology, Faculty of Science, University of Ostrava, Ostrava, Czech Republic

[4] Division of Infectious Diseases, Department of Medicine, Faculty of Medicine and Dentistry, University of Alberta, Edmonton, Canada

[5] Centre for Comparative Genomics and Evolutionary Bioinformatics, and Department of Biochemistry and Molecular Biology, Dalhousie University, Halifax, Canada

[6] Current address: Microbiology research group, Department of Biology, Lund University, Lund, Sweden

[7] Current address: Institute of Evolutionary Biology (CSIC-Universitat Pompeu Fabra), Barcelona, Spain

[8] Department of Zoology, Faculty of Science, Charles University, Prague, Czech Republic

[*] These authors contributed equally to this work.

[#] Corresponding authors: kika.zahonova@gmail.com (KZ); vlada@natur.cuni.cz (VH)

Supplementary Figures S1-S6

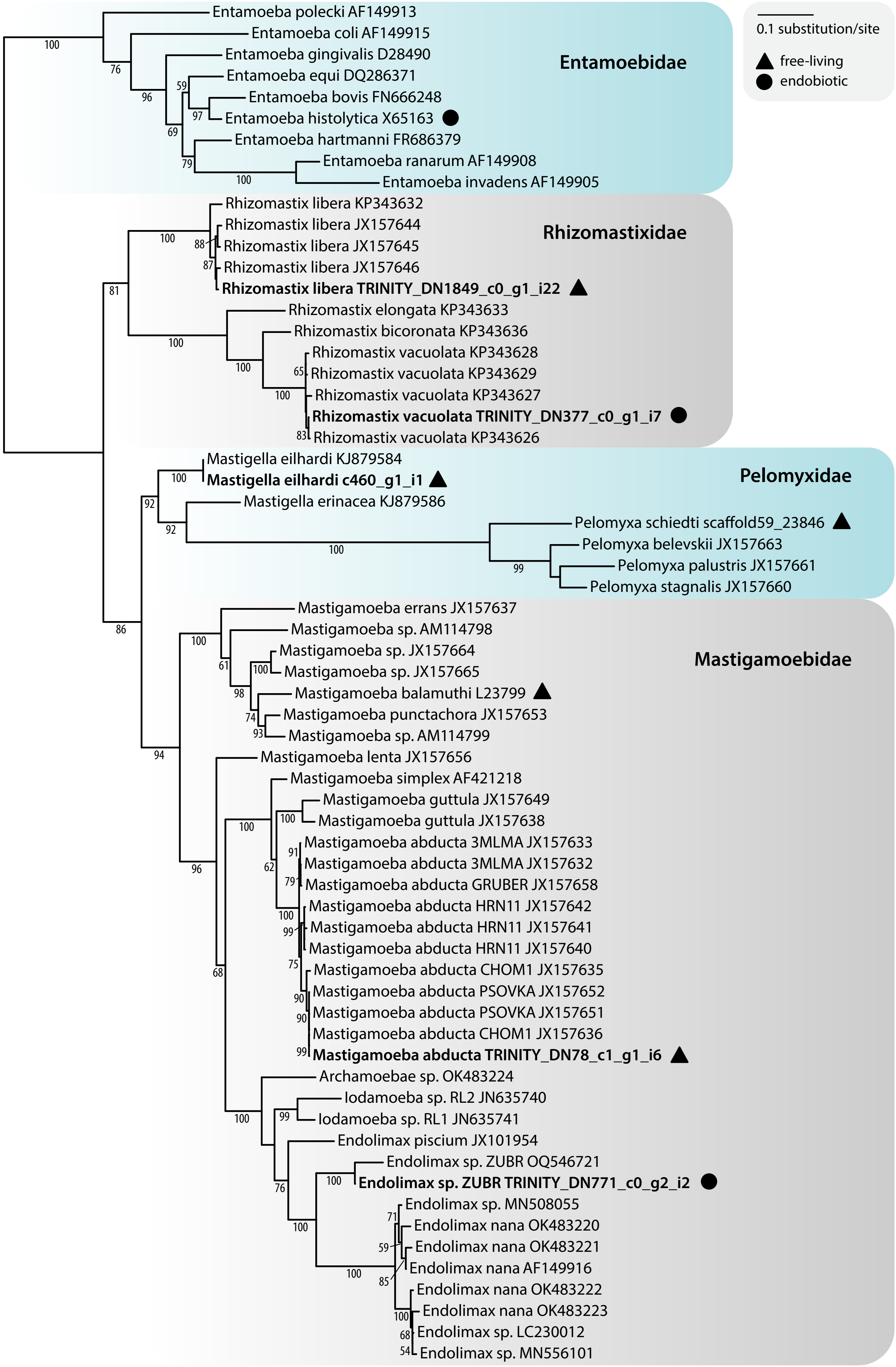

Fig. S1. Phylogenetic analysis of SSU rRNA gene of Archamoebae species. Species with new data produced within this study are in bold. All species included in the study are marked by a symbol of their lifestyle as explained in the graphical legend. Standard bootstrap support values are shown when ≥ 50%.

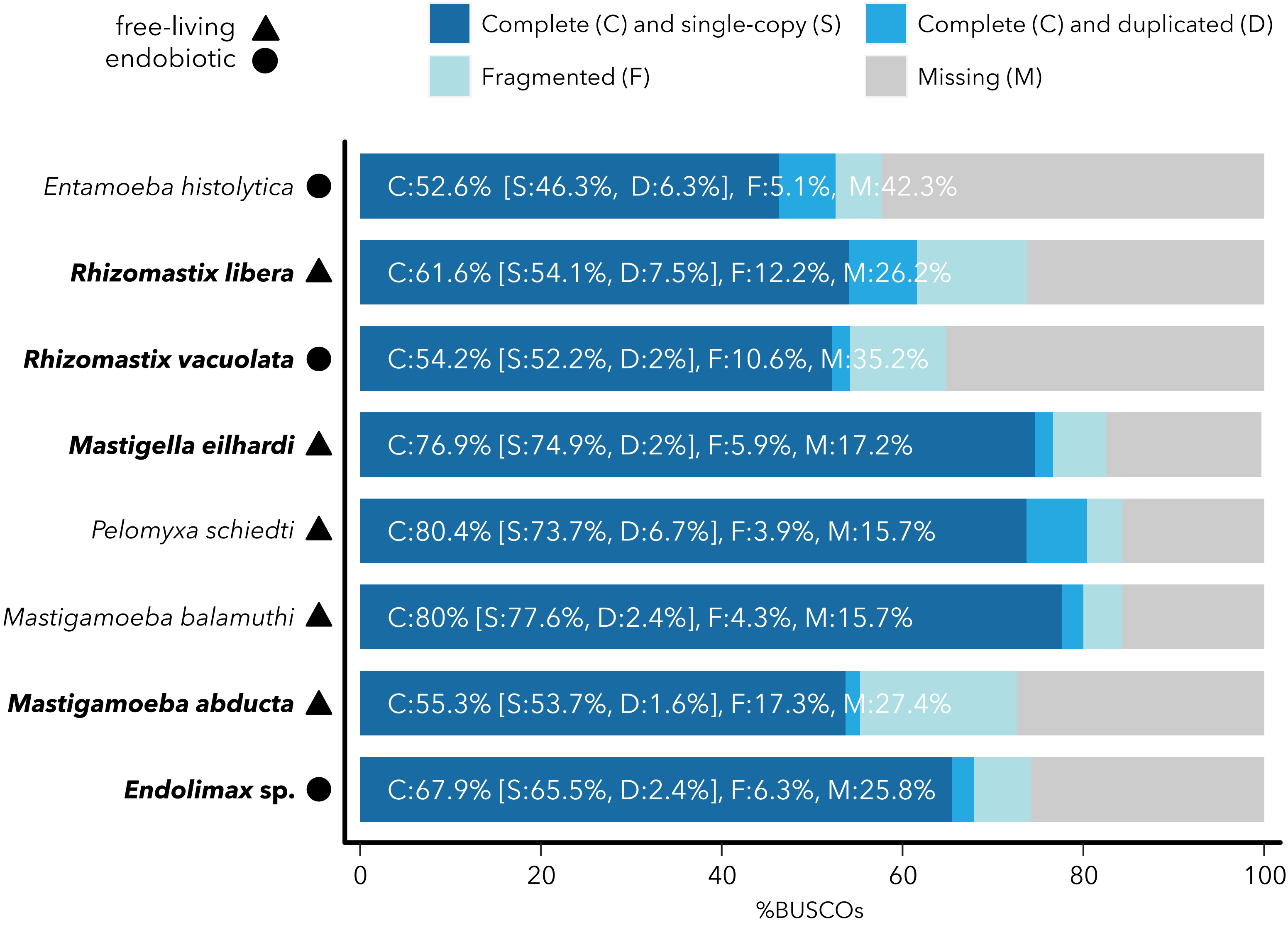

Fig. S2. BUSCO after isoform removal. The completeness of transcriptome-derived protein datasets after isoform removal (species in bold) was evaluated by BUSCO v5 using the odb10_eukaryota database and compared with the completeness of genome-derived protein datasets from *E. histolytica*, *M. balamuthi*, and *P. schiedti*. Species lifestyle is marked by pictograms explained in the graphical legend above.

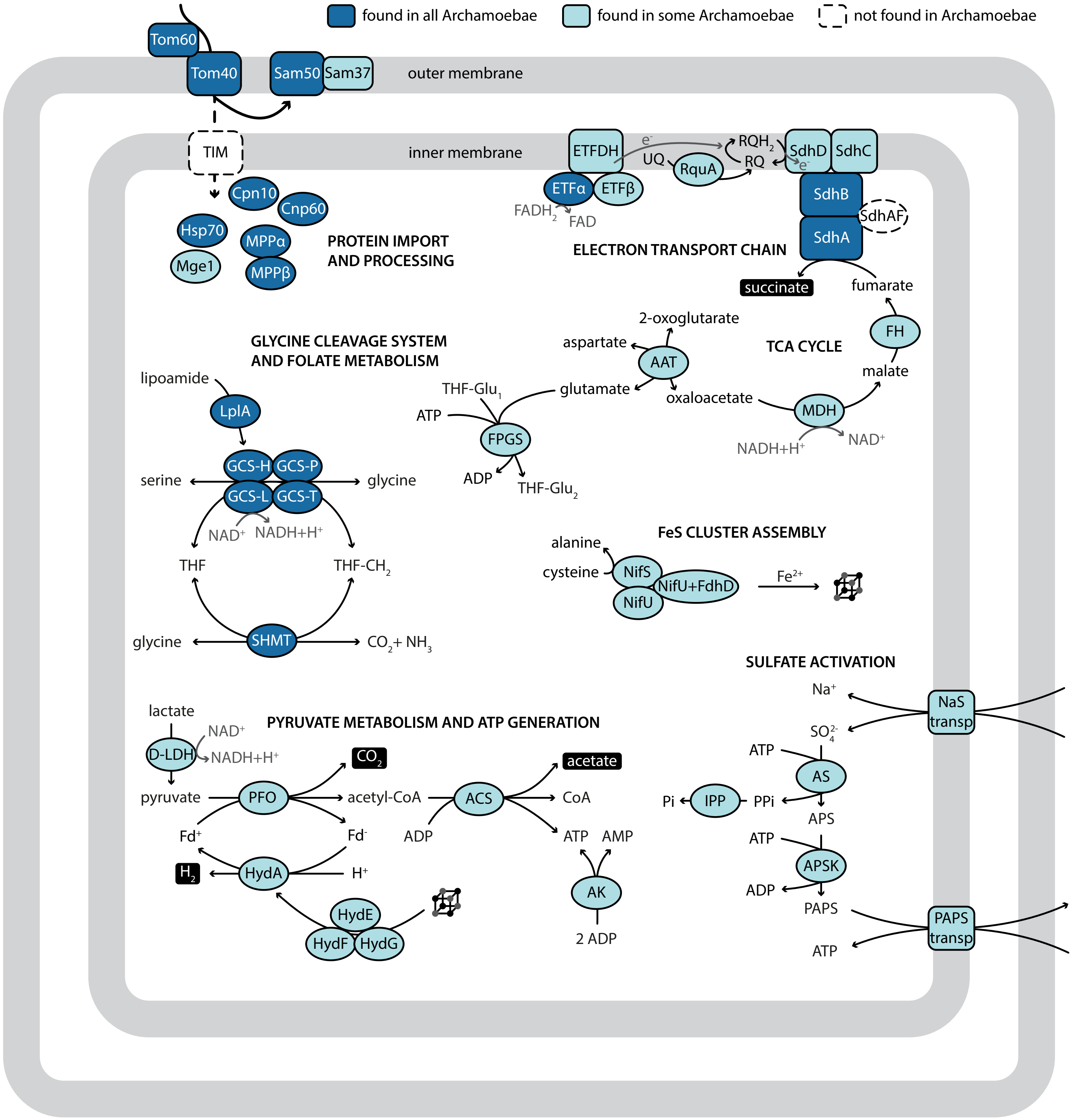

Fig. S3. Overview of predicted MRO pathways in Archamoebae. Proteins present in all or some species are shown in dark- or light-blue color, respectively, whereas missing proteins are shown in white (see graphical legend above the scheme). Reaction end products are highlighted by white font on black background. Note that *E. histolytica* was not considered, as its mitosomes harbor only the sulfate activation pathway. Abbreviations are as in Fig. 3.

A)

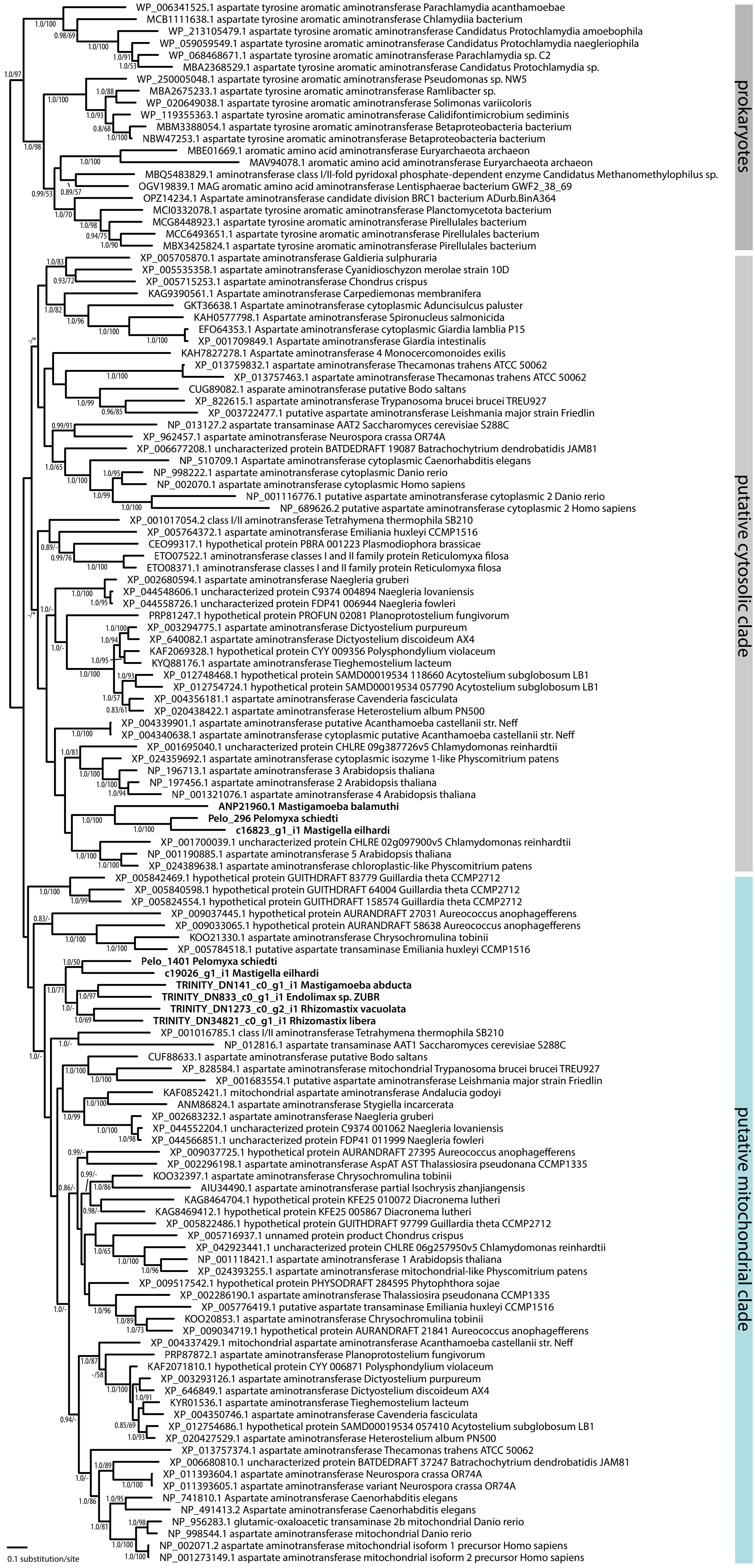

WP_006341525.1 aspartate tyrosine aromatic aminotransferase Parachlamydia acanthamoebae
MCB1111638.1 aspartate tyrosine aromatic aminotransferase Chlamydiia bacterium
WP_213105479.1 aspartate tyrosine aromatic aminotransferase Candidatus Protochlamydia amoebophila
WP_059059549.1 aspartate tyrosine aromatic aminotransferase Candidatus Protochlamydia naegleriophila
WP_068468671.1 aspartate tyrosine aromatic aminotransferase Parachlamydia sp. C2
MBA2368529.1 aspartate tyrosine aromatic aminotransferase Candidatus Protochlamydia sp.
WP_250005048.1 aspartate tyrosine aromatic aminotransferase Pseudomonas sp. NW5
MBA2675233.1 aspartate tyrosine aromatic aminotransferase Ramlibacter sp.
WP_020649038.1 aspartate tyrosine aromatic aminotransferase Solimonas variicoloris
WP_119355363.1 aspartate tyrosine aromatic aminotransferase Calidifontimicrobium sediminis
MBM3388054.1 aspartate tyrosine aromatic aminotransferase Betaproteobacteria bacterium
NBW47253.1 aspartate tyrosine aromatic aminotransferase Betaproteobacteria bacterium
MBE01669.1 aromatic amino acid aminotransferase Euryarchaeota archaeon
MAV94078.1 aromatic amino acid aminotransferase Euryarchaeota archaeon
MBQ5483829.1 aminotransferase class I/II-fold pyridoxal phosphate-dependent enzyme Candidatus Methanomethylophilus sp.
OGV19839.1 MAG aromatic amino acid aminotransferase Lentisphaerae bacterium GWF2_38_69
OPZ14234.1 Aspartate aminotransferase candidate division BRC1 bacterium ADurb.BinA364
MCI0332078.1 aspartate tyrosine aromatic aminotransferase Planctomycetota bacterium
MCG8448923.1 aspartate tyrosine aromatic aminotransferase Pirellulales bacterium
MCC6493651.1 aspartate tyrosine aromatic aminotransferase Pirellulales bacterium
MBX3425824.1 aspartate tyrosine aromatic aminotransferase Pirellulales bacterium
prokaryotes
XP_005705870.1 aspartate aminotransferase Galdieria sulphuraria
XP_005535358.1 aspartate aminotransferase Cyanidioschyzon merolae strain 10D
XP_005715253.1 aspartate aminotransferase Chondrus crispus
KAG9390561.1 Aspartate aminotransferase Carpediemonas membranifera
GKT36638.1 Aspartate aminotransferase cytoplasmic Aduncisulcus paluster
KAH0577798.1 Aspartate aminotransferase Spironucleus salmonicida
EFO64353.1 Aspartate aminotransferase cytoplasmic Giardia lamblia P15
XP_001709849.1 Aspartate aminotransferase Giardia intestinalis
KAH7827278.1 Aspartate aminotransferase 4 Monocercomonoides exilis
XP_013759832.1 aspartate aminotransferase Thecamonas trahens ATCC 50062
XP_013757463.1 aspartate aminotransferase Thecamonas trahens ATCC 50062
CUG89082.1 asparate aminotransferase putative Bodo saltans
XP_822615.1 aspartate aminotransferase Trypanosoma brucei brucei TREU927
XP_003722477.1 putative aspartate aminotransferase Leishmania major strain Friedlin
NP_013127.2 aspartate transaminase AAT2 Saccharomyces cerevisiae S288C
XP_962457.1 aspartate aminotransferase Neurospora crassa OR74A
XP_006677208.1 uncharacterized protein BATDEDRAFT 19087 Batrachochytrium dendrobatidis JAM81
NP_510709.1 Aspartate aminotransferase cytoplasmic Caenorhabditis elegans
NP_998222.1 aspartate aminotransferase cytoplasmic Danio rerio
NP_002070.1 aspartate aminotransferase cytoplasmic Homo sapiens
NP_001116776.1 putative aspartate aminotransferase cytoplasmic 2 Danio rerio
NP_689626.2 putative aspartate aminotransferase cytoplasmic 2 Homo sapiens
XP_001017054.2 class I/II aminotransferase Tetrahymena thermophila SB210
XP_005764372.1 aspartate aminotransferase Emiliania huxleyi CCMP1516
CEO99317.1 hypothetical protein PBRA 001223 Plasmodiophora brassicae
ETO07522.1 aminotransferase classes I and II family protein Reticulomyxa filosa
ETO08371.1 aminotransferase classes I and II family protein Reticulomyxa filosa
XP_002680594.1 aspartate aminotransferase Naegleria gruberi
XP_044548606.1 uncharacterized protein C9374 004894 Naegleria lovaniensis
XP_044558726.1 uncharacterized protein FDP41 006944 Naegleria fowleri
PRP81247.1 hypothetical protein PROFUN 02081 Planoprotostelium fungivorum
XP_003294775.1 aspartate aminotransferase Dictyostelium purpureum
XP_640082.1 aspartate aminotransferase Dictyostelium discoideum AX4
KAF2069328.1 hypothetical protein CYY 009356 Polysphondylium violaceum
KYQ88176.1 aspartate aminotransferase Tieghemostelium lacteum
XP_012748468.1 hypothetical protein SAMD00019534 118660 Acytostelium subglobosum LB1
XP_012754724.1 hypothetical protein SAMD00019534 057790 Acytostelium subglobosum LB1
XP_004356181.1 aspartate aminotransferase Cavenderia fasciculata
XP_020438422.1 aspartate aminotransferase Heterostelium album PN500
XP_004339901.1 aspartate aminotransferase putative Acanthamoeba castellanii str. Neff
XP_004340638.1 aspartate aminotransferase cytoplasmic putative Acanthamoeba castellanii str. Neff
XP_001695040.1 uncharacterized protein CHLRE 09g387726v5 Chlamydomonas reinhardtii
XP_024359692.1 aspartate aminotransferase cytoplasmic isozyme 1-like Physcomitrium patens
NP_196713.1 aspartate aminotransferase 3 Arabidopsis thaliana
NP_197456.1 aspartate aminotransferase 2 Arabidopsis thaliana
NP_001321076.1 aspartate aminotransferase 4 Arabidopsis thaliana
ANP21960.1 Mastigamoeba balamuthi
Pelo_296 Pelomyxa schiedti
c16823_g1_i1 Mastigella eilhardi
XP_001700039.1 uncharacterized protein CHLRE 02g097900v5 Chlamydomonas reinhardtii
NP_001190885.1 aspartate aminotransferase 5 Arabidopsis thaliana
XP_024389638.1 aspartate aminotransferase chloroplastic-like Physcomitrium patens
putative cytosolic clade
XP_005842469.1 hypothetical protein GUITHDRAFT 83779 Guillardia theta CCMP2712
XP_005840598.1 hypothetical protein GUITHDRAFT 64004 Guillardia theta CCMP2712
XP_005824554.1 hypothetical protein GUITHDRAFT 158574 Guillardia theta CCMP2712
XP_009037445.1 hypothetical protein AURANDRAFT 27031 Aureococcus anophagefferens
XP_009033065.1 hypothetical protein AURANDRAFT 58638 Aureococcus anophagefferens
KOO21330.1 aspartate aminotransferase Chrysochromulina tobinii
XP_005784518.1 putative aspartate transaminase Emiliania huxleyi CCMP1516
Pelo_1401 Pelomyxa schiedti
c19026_g1_i1 Mastigella eilhardi
TRINITY_DN141_c0_g1_i1 Mastigamoeba abducta
TRINITY_DN833_c0_g1_i1 Endolimax sp. ZUBR
TRINITY_DN1273_c0_g2_i1 Rhizomastix vacuolata
TRINITY_DN34821_c0_g1_i1 Rhizomastix libera
XP_001016785.1 class I/II aminotransferase Tetrahymena thermophila SB210
NP_012816.1 aspartate transaminase AAT1 Saccharomyces cerevisiae S288C
CUF88633.1 aspartate aminotransferase putative Bodo saltans
XP_828584.1 aspartate aminotransferase mitochondrial Trypanosoma brucei brucei TREU927
XP_001683554.1 putative aspartate aminotransferase Leishmania major strain Friedlin
KAF0852421.1 mitochondrial aspartate aminotransferase Andalucia godoyi
ANM86824.1 aspartate aminotransferase Stygiella incarcerata
XP_002683232.1 aspartate aminotransferase Naegleria gruberi
XP_044552204.1 uncharacterized protein C9374 001062 Naegleria lovaniensis
XP_044566851.1 uncharacterized protein FDP41 011999 Naegleria fowleri
XP_009037725.1 hypothetical protein AURANDRAFT 27395 Aureococcus anophagefferens
XP_002296198.1 aspartate aminotransferase AspAT AST Thalassiosira pseudonana CCMP1335
KOO32397.1 aspartate aminotransferase Chrysochromulina tobinii
AIU34490.1 aspartate aminotransferase partial Isochrysis zhanjiangensis
KAG8464704.1 hypothetical protein KFE25 010072 Diacronema lutheri
KAG8469412.1 hypothetical protein KFE25 005867 Diacronema lutheri
XP_005822486.1 hypothetical protein GUITHDRAFT 97799 Guillardia theta CCMP2712
XP_005716937.1 unnamed protein product Chondrus crispus
XP_042923441.1 uncharacterized protein CHLRE 06g257950v5 Chlamydomonas reinhardtii
NP_001118421.1 aspartate aminotransferase 1 Arabidopsis thaliana
XP_024393255.1 aspartate aminotransferase mitochondrial-like Physcomitrium patens
XP_009517542.1 hypothetical protein PHYSODRAFT 284595 Phytophthora sojae
XP_002286190.1 aspartate aminotransferase Thalassiosira pseudonana CCMP1335
XP_005776419.1 putative aspartate transaminase Emiliania huxleyi CCMP1516
KOO20853.1 aspartate aminotransferase Chrysochromulina tobinii
XP_009034719.1 hypothetical protein AURANDRAFT 21841 Aureococcus anophagefferens
XP_004337429.1 mitochondrial aspartate aminotransferase Acanthamoeba castellanii str. Neff
PRP87872.1 aspartate aminotransferase Planoprotostelium fungivorum
KAF2071810.1 hypothetical protein CYY 006871 Polysphondylium violaceum
XP_003293126.1 aspartate aminotransferase Dictyostelium purpureum
XP_646849.1 aspartate aminotransferase Dictyostelium discoideum AX4
KYR01536.1 aspartate aminotransferase Tieghemostelium lacteum
XP_004350746.1 aspartate aminotransferase Cavenderia fasciculata
XP_012754686.1 hypothetical protein SAMD00019534 057410 Acytostelium subglobosum LB1
XP_020427529.1 aspartate aminotransferase Heterostelium album PN500
XP_013757374.1 aspartate aminotransferase Thecamonas trahens ATCC 50062
XP_006680810.1 uncharacterized protein BATDEDRAFT 37247 Batrachochytrium dendrobatidis JAM81
XP_011393604.1 aspartate aminotransferase Neurospora crassa OR74A
XP_011393605.1 aspartate aminotransferase variant Neurospora crassa OR74A
NP_741810.1 Aspartate aminotransferase Caenorhabditis elegans
NP_491413.2 Aspartate aminotransferase Caenorhabditis elegans
NP_956283.1 glutamic-oxaloacetic transaminase 2b mitochondrial Danio rerio
NP_998544.1 aspartate aminotransferase mitochondrial Danio rerio
NP_002071.2 aspartate aminotransferase mitochondrial isoform 1 precursor Homo sapiens
NP_001273149.1 aspartate aminotransferase mitochondrial isoform 2 precursor Homo sapiens
putative mitochondrial clade
0.1 substitution/site

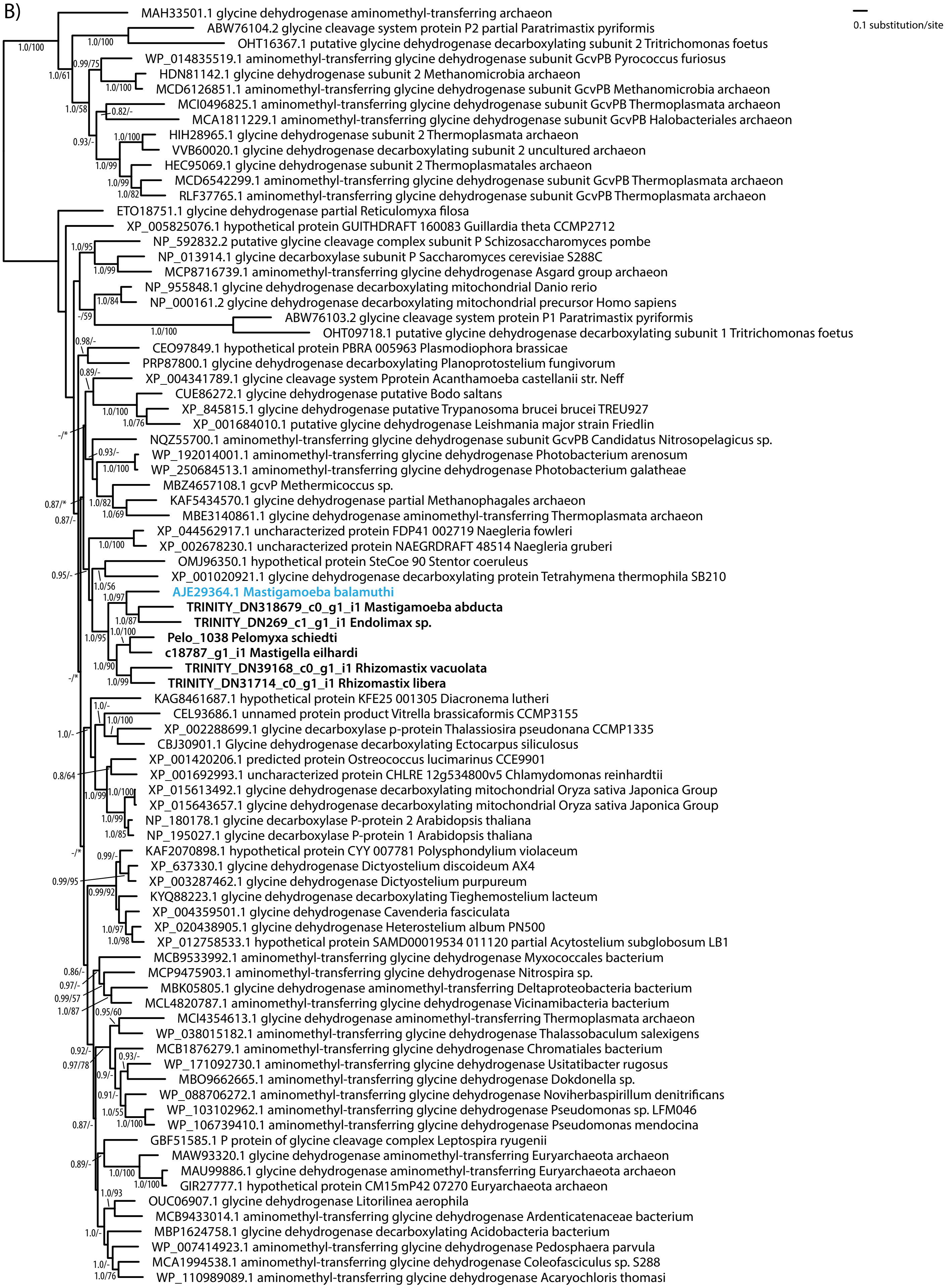
B)
0.1 substitution/site
MAH33501.1 glycine dehydrogenase aminomethyl-transferring archaeon
ABW76104.2 glycine cleavage system protein P2 partial Paratrimastix pyriformis
OHT16367.1 putative glycine dehydrogenase decarboxylating subunit 2 Tritrichomonas foetus
WP_014835519.1 aminomethyl-transferring glycine dehydrogenase subunit GcvPB Pyrococcus furiosus
HDN81142.1 glycine dehydrogenase subunit 2 Methanomicrobia archaeon
MCD6126851.1 aminomethyl-transferring glycine dehydrogenase subunit GcvPB Methanomicrobia archaeon
MCI0496825.1 aminomethyl-transferring glycine dehydrogenase subunit GcvPB Thermoplasmata archaeon
MCA1811229.1 aminomethyl-transferring glycine dehydrogenase subunit GcvPB Halobacteriales archaeon
HIH28965.1 glycine dehydrogenase subunit 2 Thermoplasmata archaeon
VVB60020.1 glycine dehydrogenase decarboxylating subunit 2 uncultured archaeon
HEC95069.1 glycine dehydrogenase subunit 2 Thermoplasmatales archaeon
MCD6542299.1 aminomethyl-transferring glycine dehydrogenase subunit GcvPB Thermoplasmata archaeon
RLF37765.1 aminomethyl-transferring glycine dehydrogenase subunit GcvPB Thermoplasmata archaeon
ETO18751.1 glycine dehydrogenase partial Reticulomyxa filosa
XP_005825076.1 hypothetical protein GUITHDRAFT 160083 Guillardia theta CCMP2712
NP_592832.2 putative glycine cleavage complex subunit P Schizosaccharomyces pombe
NP_013914.1 glycine decarboxylase subunit P Saccharomyces cerevisiae S288C
MCP8716739.1 aminomethyl-transferring glycine dehydrogenase Asgard group archaeon
NP_955848.1 glycine dehydrogenase decarboxylating mitochondrial Danio rerio
NP_000161.2 glycine dehydrogenase decarboxylating mitochondrial precursor Homo sapiens
ABW76103.2 glycine cleavage system protein P1 Paratrimastix pyriformis
OHT09718.1 putative glycine dehydrogenase decarboxylating subunit 1 Tritrichomonas foetus
CEO97849.1 hypothetical protein PBRA 005963 Plasmodiophora brassicae
PRP87800.1 glycine dehydrogenase decarboxylating Planoprotostelium fungivorum
XP_004341789.1 glycine cleavage system Pprotein Acanthamoeba castellanii str. Neff
CUE86272.1 glycine dehydrogenase putative Bodo saltans
XP_845815.1 glycine dehydrogenase putative Trypanosoma brucei brucei TREU927
XP_001684010.1 putative glycine dehydrogenase Leishmania major strain Friedlin
NQZ55700.1 aminomethyl-transferring glycine dehydrogenase subunit GcvPB Candidatus Nitrosopelagicus sp.
WP_192014001.1 aminomethyl-transferring glycine dehydrogenase Photobacterium arenosum
WP_250684513.1 aminomethyl-transferring glycine dehydrogenase Photobacterium galatheae
MBZ4657108.1 gcvP Methermicoccus sp.
KAF5434570.1 glycine dehydrogenase partial Methanophagales archaeon
MBE3140861.1 glycine dehydrogenase aminomethyl-transferring Thermoplasmata archaeon
XP_044562917.1 uncharacterized protein FDP41 002719 Naegleria fowleri
XP_002678230.1 uncharacterized protein NAEGRDRAFT 48514 Naegleria gruberi
OMJ96350.1 hypothetical protein SteCoe 90 Stentor coeruleus
XP_001020921.1 glycine dehydrogenase decarboxylating protein Tetrahymena thermophila SB210
AJE29364.1 Mastigamoeba balamuthi
TRINITY_DN318679_c0_g1_i1 Mastigamoeba abducta
TRINITY_DN269_c1_g1_i1 Endolimax sp.
Pelo_1038 Pelomyxa schiedti
c18787_g1_i1 Mastigella eilhardi
TRINITY_DN39168_c0_g1_i1 Rhizomastix vacuolata
TRINITY_DN31714_c0_g1_i1 Rhizomastix libera
KAG8461687.1 hypothetical protein KFE25 001305 Diacronema lutheri
CEL93686.1 unnamed protein product Vitrella brassicaformis CCMP3155
XP_002288699.1 glycine decarboxylase p-protein Thalassiosira pseudonana CCMP1335
CBJ30901.1 Glycine dehydrogenase decarboxylating Ectocarpus siliculosus
XP_001420206.1 predicted protein Ostreococcus lucimarinus CCE9901
XP_001692993.1 uncharacterized protein CHLRE 12g534800v5 Chlamydomonas reinhardtii
XP_015613492.1 glycine dehydrogenase decarboxylating mitochondrial Oryza sativa Japonica Group
XP_015643657.1 glycine dehydrogenase decarboxylating mitochondrial Oryza sativa Japonica Group
NP_180178.1 glycine decarboxylase P-protein 2 Arabidopsis thaliana
NP_195027.1 glycine decarboxylase P-protein 1 Arabidopsis thaliana
KAF2070898.1 hypothetical protein CYY 007781 Polysphondylium violaceum
XP_637330.1 glycine dehydrogenase Dictyostelium discoideum AX4
XP_003287462.1 glycine dehydrogenase Dictyostelium purpureum
KYQ88223.1 glycine dehydrogenase decarboxylating Tieghemostelium lacteum
XP_004359501.1 glycine dehydrogenase Cavenderia fasciculata
XP_020438905.1 glycine dehydrogenase Heterostelium album PN500
XP_012758533.1 hypothetical protein SAMD00019534 011120 partial Acytostelium subglobosum LB1
MCB9533992.1 aminomethyl-transferring glycine dehydrogenase Myxococcales bacterium
MCP9475903.1 aminomethyl-transferring glycine dehydrogenase Nitrospira sp.
MBK05805.1 glycine dehydrogenase aminomethyl-transferring Deltaproteobacteria bacterium
MCL4820787.1 aminomethyl-transferring glycine dehydrogenase Vicinamibacteria bacterium
MCI4354613.1 glycine dehydrogenase aminomethyl-transferring Thermoplasmata archaeon
WP_038015182.1 aminomethyl-transferring glycine dehydrogenase Thalassobaculum salexigens
MCB1876279.1 aminomethyl-transferring glycine dehydrogenase Chromatiales bacterium
WP_171092730.1 aminomethyl-transferring glycine dehydrogenase Usitatibacter rugosus
MBO9662665.1 aminomethyl-transferring glycine dehydrogenase Dokdonella sp.
WP_088706272.1 aminomethyl-transferring glycine dehydrogenase Noviherbaspirillum denitrificans
WP_103102962.1 aminomethyl-transferring glycine dehydrogenase Pseudomonas sp. LFM046
WP_106739410.1 aminomethyl-transferring glycine dehydrogenase Pseudomonas mendocina
GBF51585.1 P protein of glycine cleavage complex Leptospira ryugenii
MAW93320.1 glycine dehydrogenase aminomethyl-transferring Euryarchaeota archaeon
MAU99886.1 glycine dehydrogenase aminomethyl-transferring Euryarchaeota archaeon
GIR27777.1 hypothetical protein CM15mP42 07270 Euryarchaeota archaeon
OUC06907.1 glycine dehydrogenase Litorilinea aerophila
MCB9433014.1 aminomethyl-transferring glycine dehydrogenase Ardenticatenaceae bacterium
MBP1624758.1 glycine dehydrogenase decarboxylating Acidobacteria bacterium
WP_007414923.1 aminomethyl-transferring glycine dehydrogenase Pedosphaera parvula
MCA1994538.1 aminomethyl-transferring glycine dehydrogenase Coleofasciculus sp. S288
WP_110989089.1 aminomethyl-transferring glycine dehydrogenase Acaryochloris thomasi

C)

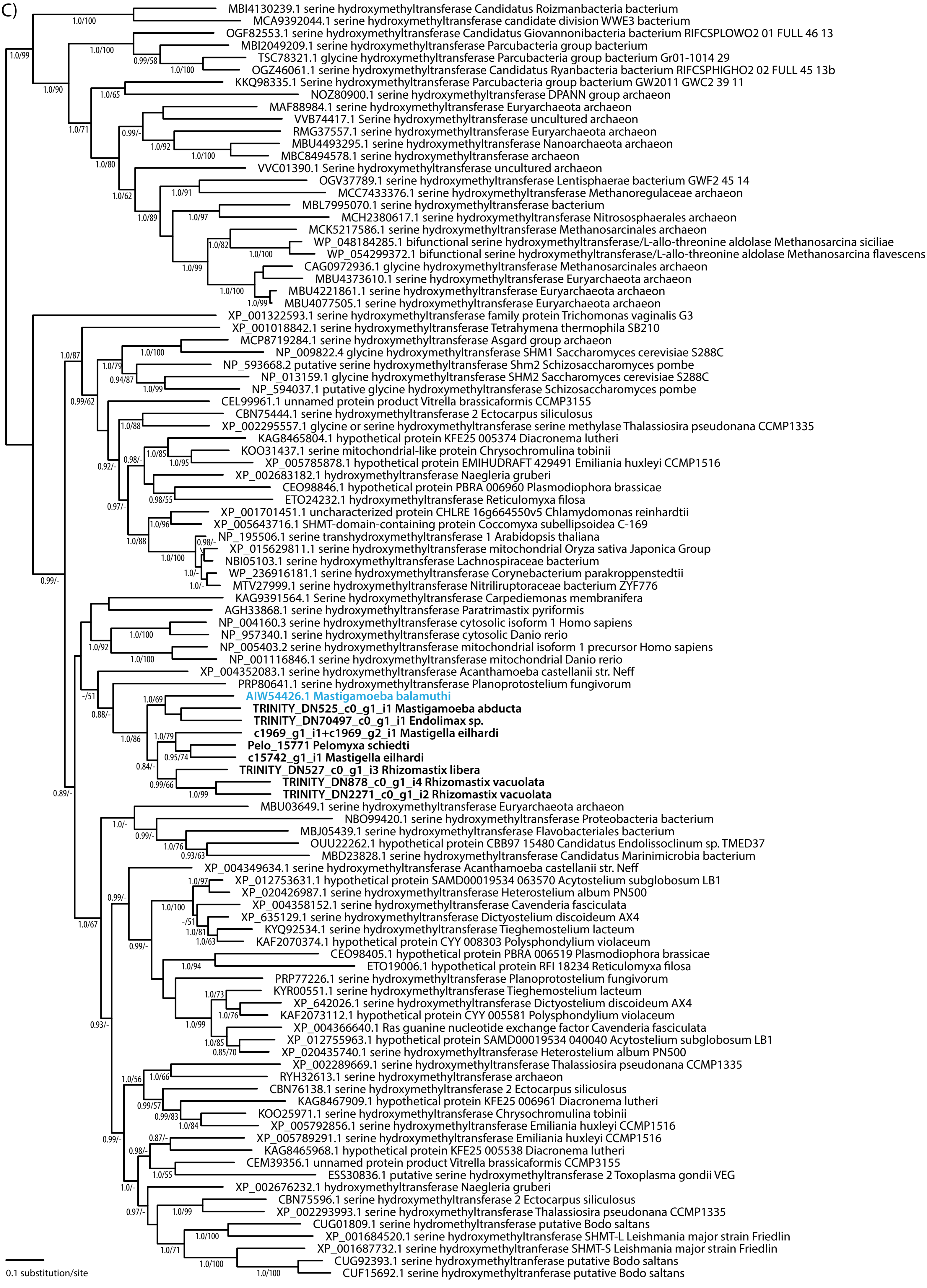
MBI4130239.1 serine hydroxymethyltransferase Candidatus Roizmanbacteria bacterium
MCA9392044.1 serine hydroxymethyltransferase candidate division WWE3 bacterium
OGF82553.1 serine hydroxymethyltransferase Candidatus Giovannonibacteria bacterium RIFCSPLOWO2 01 FULL 46 13
MBI2049209.1 serine hydroxymethyltransferase Parcubacteria group bacterium
TSC78321.1 glycine hydroxymethyltransferase Parcubacteria group bacterium Gr01-1014 29
OGZ46061.1 serine hydroxymethyltransferase Candidatus Ryanbacteria bacterium RIFCSPHIGHO2 02 FULL 45 13b
KKQ98335.1 Serine hydroxymethyltransferase Parcubacteria group bacterium GW2011 GWC2 39 11
NOZ80900.1 serine hydroxymethyltransferase DPANN group archaeon
MAF88984.1 serine hydroxymethyltransferase Euryarchaeota archaeon
VVB74417.1 Serine hydroxymethyltransferase uncultured archaeon
RMG37557.1 serine hydroxymethyltransferase Euryarchaeota archaeon
MBU4493295.1 serine hydroxymethyltransferase Nanoarchaeota archaeon
MBC8494578.1 serine hydroxymethyltransferase archaeon
VVC01390.1 Serine hydroxymethyltransferase uncultured archaeon
OGV37789.1 serine hydroxymethyltransferase Lentisphaerae bacterium GWF2 45 14
MCC7433376.1 serine hydroxymethyltransferase Methanoregulaceae archaeon
MBL7995070.1 serine hydroxymethyltransferase bacterium
MCH2380617.1 serine hydroxymethyltransferase Nitrososphaerales archaeon
MCK5217586.1 serine hydroxymethyltransferase Methanosarcinales archaeon
WP_048184285.1 bifunctional serine hydroxymethyltransferase/L-allo-threonine aldolase Methanosarcina siciliae
WP_054299372.1 bifunctional serine hydroxymethyltransferase/L-allo-threonine aldolase Methanosarcina flavescens
CAG0972936.1 glycine hydroxymethyltransferase Methanosarcinales archaeon
MBU4373610.1 serine hydroxymethyltransferase Euryarchaeota archaeon
MBU4221861.1 serine hydroxymethyltransferase Euryarchaeota archaeon
MBU4077505.1 serine hydroxymethyltransferase Euryarchaeota archaeon
XP_001322593.1 serine hydroxymethyltransferase family protein Trichomonas vaginalis G3
XP_001018842.1 serine hydroxymethyltransferase Tetrahymena thermophila SB210
MCP8719284.1 serine hydroxymethyltransferase Asgard group archaeon
NP_009822.4 glycine hydroxymethyltransferase SHM1 Saccharomyces cerevisiae S288C
NP_593668.2 putative serine hydroxymethyltransferase Shm2 Schizosaccharomyces pombe
NP_013159.1 glycine hydroxymethyltransferase SHM2 Saccharomyces cerevisiae S288C
NP_594037.1 putative glycine hydroxymethyltransferase Schizosaccharomyces pombe
CEL99961.1 unnamed protein product Vitrella brassicaformis CCMP3155
CBN75444.1 serine hydroxymethyltransferase 2 Ectocarpus siliculosus
XP_002295557.1 glycine or serine hydroxymethyltransferase serine methylase Thalassiosira pseudonana CCMP1335
KAG8465804.1 hypothetical protein KFE25 005374 Diacronema lutheri
KOO31437.1 serine mitochondrial-like protein Chrysochromulina tobinii
XP_005785878.1 hypothetical protein EMIHUDRAFT 429491 Emiliania huxleyi CCMP1516
XP_002683182.1 hydroxymethyltransferase Naegleria gruberi
CEO98846.1 hypothetical protein PBRA 006960 Plasmodiophora brassicae
ETO24232.1 hydroxymethyltransferase Reticulomyxa filosa
XP_001701451.1 uncharacterized protein CHLRE 16g664550v5 Chlamydomonas reinhardtii
XP_005643716.1 SHMT-domain-containing protein Coccomyxa subellipsoidea C-169
NP_195506.1 serine transhydroxymethyltransferase 1 Arabidopsis thaliana
XP_015629811.1 serine hydroxymethyltransferase mitochondrial Oryza sativa Japonica Group
NBI05103.1 serine hydroxymethyltransferase Lachnospiraceae bacterium
WP_236916181.1 serine hydroxymethyltransferase Corynebacterium parakroppenstedtii
MTV27999.1 serine hydroxymethyltransferase Nitriliruptoraceae bacterium ZYF776
KAG9391564.1 Serine hydroxymethyltransferase Carpediemonas membranifera
AGH33868.1 serine hydroxymethyltransferase Paratrimastix pyriformis
NP_004160.3 serine hydroxymethyltransferase cytosolic isoform 1 Homo sapiens
NP_957340.1 serine hydroxymethyltransferase cytosolic Danio rerio
NP_005403.2 serine hydroxymethyltransferase mitochondrial isoform 1 precursor Homo sapiens
NP_001116846.1 serine hydroxymethyltransferase mitochondrial Danio rerio
XP_004352083.1 serine hydroxymethyltransferase Acanthamoeba castellanii str. Neff
PRP80641.1 serine hydroxymethyltransferase Planoprotostelium fungivorum
AIW54426.1 Mastigamoeba balamuthi
TRINITY_DN525_c0_g1_i1 Mastigamoeba abducta
TRINITY_DN70497_c0_g1_i1 Endolimax sp.
c1969_g1_i1+c1969_g2_i1 Mastigella eilhardi
Pelo_15771 Pelomyxa schiedti
c15742_g1_i1 Mastigella eilhardi
TRINITY_DN527_c0_g1_i3 Rhizomastix libera
TRINITY_DN878_c0_g1_i4 Rhizomastix vacuolata
TRINITY_DN2271_c0_g1_i2 Rhizomastix vacuolata
MBU03649.1 serine hydroxymethyltransferase Euryarchaeota archaeon
NBO99420.1 serine hydroxymethyltransferase Proteobacteria bacterium
MBJ05439.1 serine hydroxymethyltransferase Flavobacteriales bacterium
OUU22262.1 hypothetical protein CBB97 15480 Candidatus Endolissoclinum sp. TMED37
MBD23828.1 serine hydroxymethyltransferase Candidatus Marinimicrobia bacterium
XP_004349634.1 serine hydroxymethyltransferase Acanthamoeba castellanii str. Neff
XP_012753631.1 hypothetical protein SAMD00019534 063570 Acytostelium subglobosum LB1
XP_020426987.1 serine hydroxymethyltransferase Heterostelium album PN500
XP_004358152.1 serine hydroxymethyltransferase Cavenderia fasciculata
XP_635129.1 serine hydroxymethyltransferase Dictyostelium discoideum AX4
KYQ92534.1 serine hydroxymethyltransferase Tieghemostelium lacteum
KAF2070374.1 hypothetical protein CYY 008303 Polysphondylium violaceum
CEO98405.1 hypothetical protein PBRA 006519 Plasmodiophora brassicae
ETO19006.1 hypothetical protein RFI 18234 Reticulomyxa filosa
PRP77226.1 serine hydroxymethyltransferase Planoprotostelium fungivorum
KYR00551.1 serine hydroxymethyltransferase Tieghemostelium lacteum
XP_642026.1 serine hydroxymethyltransferase Dictyostelium discoideum AX4
KAF2073112.1 hypothetical protein CYY 005581 Polysphondylium violaceum
XP_004366640.1 Ras guanine nucleotide exchange factor Cavenderia fasciculata
XP_012755963.1 hypothetical protein SAMD00019534 040040 Acytostelium subglobosum LB1
XP_020435740.1 serine hydroxymethyltransferase Heterostelium album PN500
XP_002289669.1 serine hydroxymethyltransferase Thalassiosira pseudonana CCMP1335
RYH32613.1 serine hydroxymethyltransferase archaeon
CBN76138.1 serine hydroxymethyltransferase 2 Ectocarpus siliculosus
KAG8467909.1 hypothetical protein KFE25 006961 Diacronema lutheri
KOO25971.1 serine hydroxymethyltransferase Chrysochromulina tobinii
XP_005792856.1 serine hydroxymethyltransferase Emiliania huxleyi CCMP1516
XP_005789291.1 serine hydroxymethyltransferase Emiliania huxleyi CCMP1516
KAG8465968.1 hypothetical protein KFE25 005538 Diacronema lutheri
CEM39356.1 unnamed protein product Vitrella brassicaformis CCMP3155
ESS30836.1 putative serine hydroxymethyltransferase 2 Toxoplasma gondii VEG
XP_002676232.1 hydroxymethyltransferase Naegleria gruberi
CBN75596.1 serine hydroxymethyltransferase 2 Ectocarpus siliculosus
XP_002293993.1 serine hydroxymethyltransferase Thalassiosira pseudonana CCMP1335
CUG01809.1 serine hydromethyltransferase putative Bodo saltans
XP_001684520.1 serine hydroxymethyltransferase SHMT-L Leishmania major strain Friedlin
XP_001687732.1 serine hydroxymethyltranferase SHMT-S Leishmania major strain Friedlin
CUG92393.1 serine hydroxymethyltranferase putative Bodo saltans
CUF15692.1 serine hydroxymethyltranferase putative Bodo saltans
0.1 substitution/site

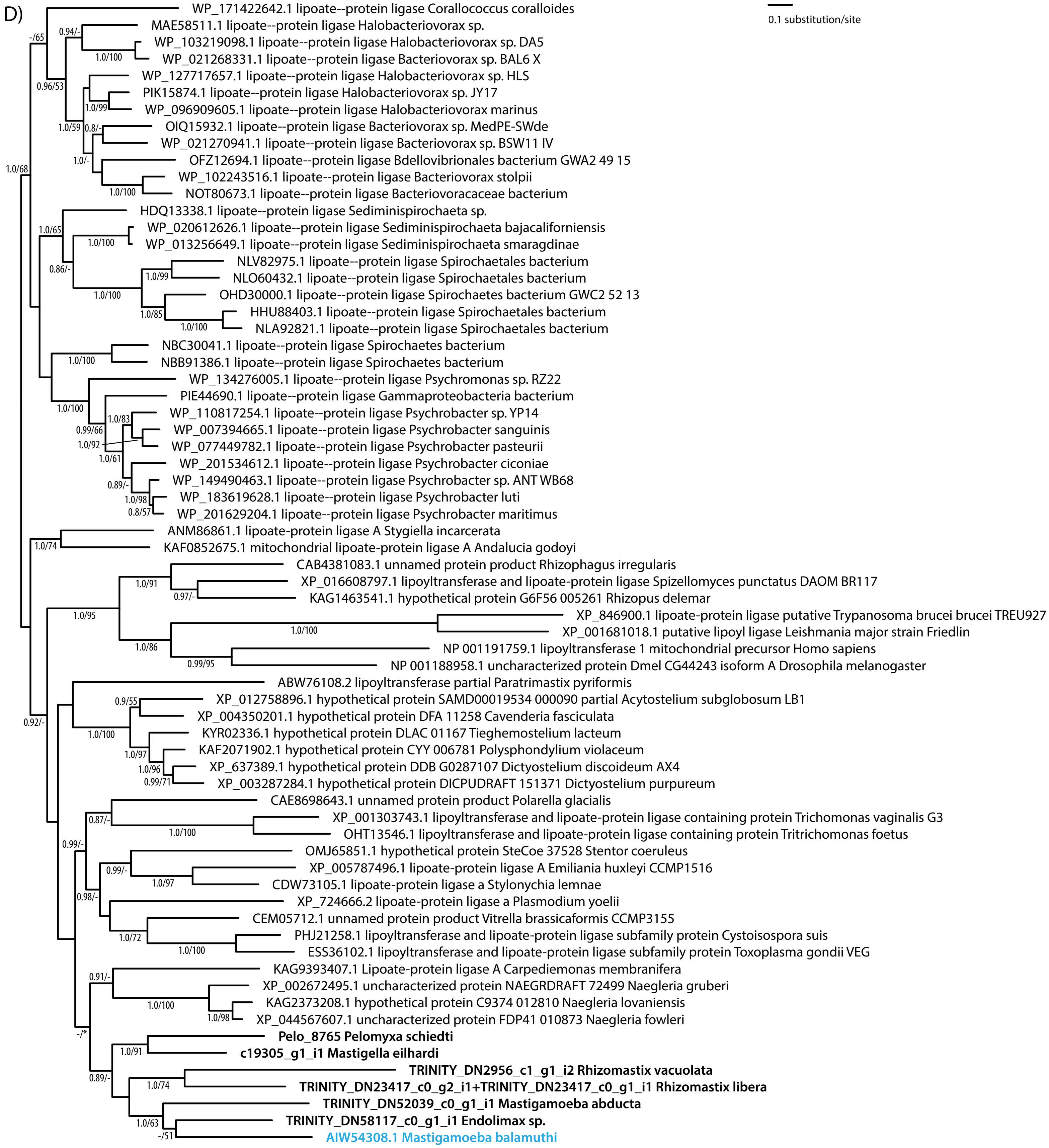
D)
0.1 substitution/site
WP_171422642.1 lipoate--protein ligase Corallococcus coralloides
MAE58511.1 lipoate--protein ligase Halobacteriovorax sp.
WP_103219098.1 lipoate--protein ligase Halobacteriovorax sp. DA5
WP_021268331.1 lipoate--protein ligase Bacteriovorax sp. BAL6 X
WP_127717657.1 lipoate--protein ligase Halobacteriovorax sp. HLS
PIK15874.1 lipoate--protein ligase Halobacteriovorax sp. JY17
WP_096909605.1 lipoate--protein ligase Halobacteriovorax marinus
OIQ15932.1 lipoate--protein ligase Bacteriovorax sp. MedPE-SWde
WP_021270941.1 lipoate--protein ligase Bacteriovorax sp. BSW11 IV
OFZ12694.1 lipoate--protein ligase Bdellovibrionales bacterium GWA2 49 15
WP_102243516.1 lipoate--protein ligase Bacteriovorax stolpii
NOT80673.1 lipoate--protein ligase Bacteriovoracaceae bacterium
HDQ13338.1 lipoate--protein ligase Sediminispirochaeta sp.
WP_020612626.1 lipoate--protein ligase Sediminispirochaeta bajacaliforniensis
WP_013256649.1 lipoate--protein ligase Sediminispirochaeta smaragdinae
NLV82975.1 lipoate--protein ligase Spirochaetales bacterium
NLO60432.1 lipoate--protein ligase Spirochaetales bacterium
OHD30000.1 lipoate--protein ligase Spirochaetes bacterium GWC2 52 13
HHU88403.1 lipoate--protein ligase Spirochaetales bacterium
NLA92821.1 lipoate--protein ligase Spirochaetales bacterium
NBC30041.1 lipoate--protein ligase Spirochaetes bacterium
NBB91386.1 lipoate--protein ligase Spirochaetes bacterium
WP_134276005.1 lipoate--protein ligase Psychromonas sp. RZ22
PIE44690.1 lipoate--protein ligase Gammaproteobacteria bacterium
WP_110817254.1 lipoate--protein ligase Psychrobacter sp. YP14
WP_007394665.1 lipoate--protein ligase Psychrobacter sanguinis
WP_077449782.1 lipoate--protein ligase Psychrobacter pasteurii
WP_201534612.1 lipoate--protein ligase Psychrobacter ciconiae
WP_149490463.1 lipoate--protein ligase Psychrobacter sp. ANT WB68
WP_183619628.1 lipoate--protein ligase Psychrobacter luti
WP_201629204.1 lipoate--protein ligase Psychrobacter maritimus
ANM86861.1 lipoate-protein ligase A Stygiella incarcerata
KAF0852675.1 mitochondrial lipoate-protein ligase A Andalucia godoyi
CAB4381083.1 unnamed protein product Rhizophagus irregularis
XP_016608797.1 lipoyltransferase and lipoate-protein ligase Spizellomyces punctatus DAOM BR117
KAG1463541.1 hypothetical protein G6F56 005261 Rhizopus delemar
XP_846900.1 lipoate-protein ligase putative Trypanosoma brucei brucei TREU927
XP_001681018.1 putative lipoyl ligase Leishmania major strain Friedlin
NP 001191759.1 lipoyltransferase 1 mitochondrial precursor Homo sapiens
NP 001188958.1 uncharacterized protein Dmel CG44243 isoform A Drosophila melanogaster
ABW76108.2 lipoyltransferase partial Paratrimastix pyriformis
XP_012758896.1 hypothetical protein SAMD00019534 000090 partial Acytostelium subglobosum LB1
XP_004350201.1 hypothetical protein DFA 11258 Cavenderia fasciculata
KYR02336.1 hypothetical protein DLAC 01167 Tieghemostelium lacteum
KAF2071902.1 hypothetical protein CYY 006781 Polysphondylium violaceum
XP_637389.1 hypothetical protein DDB G0287107 Dictyostelium discoideum AX4
XP_003287284.1 hypothetical protein DICPUDRAFT 151371 Dictyostelium purpureum
CAE8698643.1 unnamed protein product Polarella glacialis
XP_001303743.1 lipoyltransferase and lipoate-protein ligase containing protein Trichomonas vaginalis G3
OHT13546.1 lipoyltransferase and lipoate-protein ligase containing protein Tritrichomonas foetus
OMJ65851.1 hypothetical protein SteCoe 37528 Stentor coeruleus
XP_005787496.1 lipoate-protein ligase A Emiliania huxleyi CCMP1516
CDW73105.1 lipoate-protein ligase a Stylonychia lemnae
XP_724666.2 lipoate-protein ligase a Plasmodium yoelii
CEM05712.1 unnamed protein product Vitrella brassicaformis CCMP3155
PHJ21258.1 lipoyltransferase and lipoate-protein ligase subfamily protein Cystoisospora suis
ESS36102.1 lipoyltransferase and lipoate-protein ligase subfamily protein Toxoplasma gondii VEG
KAG9393407.1 Lipoate-protein ligase A Carpediemonas membranifera
XP_002672495.1 uncharacterized protein NAEGRDRAFT 72499 Naegleria gruberi
KAG2373208.1 hypothetical protein C9374 012810 Naegleria lovaniensis
XP_044567607.1 uncharacterized protein FDP41 010873 Naegleria fowleri
Pelo_8765 Pelomyxa schiedti
c19305_g1_i1 Mastigella eilhardi
TRINITY_DN2956_c1_g1_i2 Rhizomastix vacuolata
TRINITY_DN23417_c0_g2_i1+TRINITY_DN23417_c0_g1_i1 Rhizomastix libera
TRINITY_DN52039_c0_g1_i1 Mastigamoeba abducta
TRINITY_DN58117_c0_g1_i1 Endolimax sp.
AIW54308.1 Mastigamoeba balamuthi
-/65
0.94/-
1.0/100
0.96/53
1.0/99
1.0/59
0.8/-
1.0/-
1.0/100
1.0/68
1.0/65
1.0/100
0.86/-
1.0/99
1.0/100
1.0/85
1.0/100
1.0/100
1.0/100
0.99/66
1.0/83
1.0/92
1.0/61
0.89/-
1.0/98
0.8/57
1.0/74
1.0/91
0.97/-
1.0/95
1.0/100
1.0/86
0.99/95
0.92/-
0.9/55
1.0/100
1.0/97
1.0/96
0.99/71
0.87/-
1.0/100
0.99/-
0.99/-
1.0/97
0.98/-
1.0/72
1.0/100
0.91/-
1.0/100
1.0/98
-/*
1.0/91
0.89/-
1.0/74
1.0/63
-/51

E)

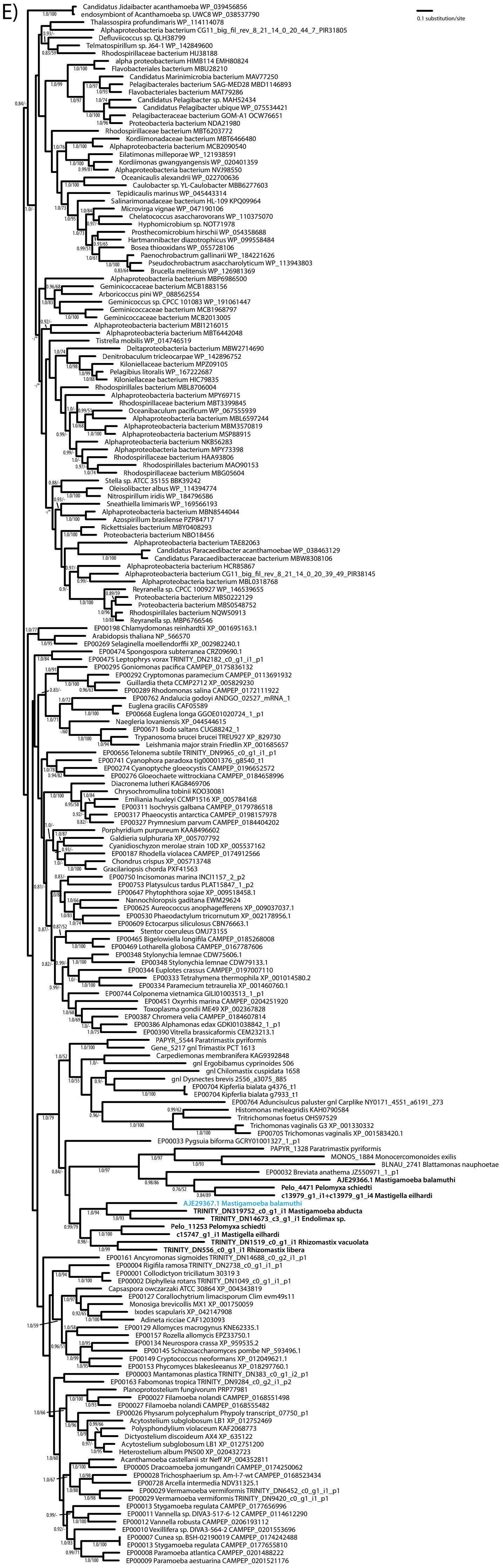

0.1 substitution/site
Candidatus Jidaibacter acanthamoeba WP_039456856
endosymbiont of Acanthamoeba sp. UWC8 WP_038537790
Thalassospira profundimaris WP_114114078
Alphaproteobacteria bacterium CG11_big_fil_rev_8_21_14_0_20_44_7_PIR31805
Defluviicoccus sp. QLH38799
Telmatospirillum sp. J64-1 WP_142849600
Rhodospirillaceae bacterium HIJ38188
alpha proteobacterium HIMB114 EMH80824
Flavobacteriales bacterium MBU28210
Candidatus Marinimicrobia bacterium MAV77250
Pelagibacterales bacterium SAG-MED28 MBD1146893
Flavobacteriales bacterium MAT79286
Candidatus Pelagibacter sp. MAH52434
Candidatus Pelagibacter ubique WP_075534421
Pelagibacteraceae bacterium GOM-A1 OCW76651
Proteobacteria bacterium NDA21980
Rhodospirillaceae bacterium MBT6203772
Kordiimonadaceae bacterium MBT6466480
Alphaproteobacteria bacterium MCB2090540
Eilatimonas milleporae WP_121938591
Kordiimonas gwangyangensis WP_020401359
Alphaproteobacteria bacterium NVJ98550
Oceanicaulis alexandrii WP_022700636
Caulobacter sp. YL-Caulobacter MBB6277603
Tepidicaulis marinus WP_045443314
Salinarimonadaceae bacterium HL-109 KPQ09964
Microvirga vignae WP_047190106
Chelatococcus asaccharovorans WP_110375070
Hyphomicrobium sp. NOT71978
Prosthecomicrobium hirschii WP_054358688
Hartmannibacter diazotrophicus WP_099558484
Bosea thiooxidans WP_055728106
Paenochrobactrum gallinarii WP_184221626
Pseudochrobactrum asaccharolyticum WP_113943803
Brucella melitensis WP_126981369
Alphaproteobacteria bacterium MBP6986500
Geminicoccaceae bacterium MCB1883156
Arboricoccus pini WP_088562554
Geminicoccus sp. CPCC 101083 WP_191061447
Geminicoccaceae bacterium MCB1968797
Geminicoccaceae bacterium MCB2013005
Alphaproteobacteria bacterium MBI1216015
Alphaproteobacteria bacterium MBT6442048
Tistrella mobilis WP_014746519
Deltaproteobacteria bacterium MBW2714690
Denitrobaculum tricleocarpae WP_142896752
Kiloniellaceae bacterium MPZ09105
Pelagibius litoralis WP_167222687
Kiloniellaceae bacterium HIC79835
Rhodospirillales bacterium MBL8706004
Alphaproteobacteria bacterium MPY69715
Rhodospirillaceae bacterium MBT3399845
Oceanibaculum pacificum WP_067555939
Alphaproteobacteria bacterium MBL6597244
Alphaproteobacteria bacterium MBM3570819
Alphaproteobacteria bacterium MSP88915
Alphaproteobacteria bacterium NKB56283
Alphaproteobacteria bacterium MPY73398
Rhodospirillaceae bacterium HAA93806
Rhodospirillales bacterium MAO90153
Rhodospirillaceae bacterium MBG05604
Stella sp. ATCC 35155 BBK39242
Oleisolibacter albus WP_114394774
Nitrospirillum iridis WP_184796586
Sneathiella limimaris WP_169566193
Alphaproteobacteria bacterium MBN8544044
Azospirillum brasilense PZP84717
Rickettsiales bacterium MBY0408293
Proteobacteria bacterium NBO18456
Alphaproteobacteria bacterium TAE82063
Candidatus Paracaedibacter acanthamoebae WP_038463129
Candidatus Paracaedibacteraceae bacterium MBW8308106
Alphaproteobacteria bacterium HCR85867
Alphaproteobacteria bacterium CG11_big_fil_rev_8_21_14_0_20_39_49_PIR38145
Alphaproteobacteria bacterium MBL0318768
Reyranella sp. CPCC 100927 WP_146539655
Proteobacteria bacterium MBS0222129
Proteobacteria bacterium MBS0548752
Rhodospirillales bacterium NQW50913
Reyranella sp. MBP6766546
EP00198 Chlamydomonas reinhardtii XP_001695163.1
Arabidopsis thaliana NP_566570
EP00269 Selaginella moellendorffii XP_002982240.1
EP00474 Spongospora subterranea CRZ09690.1
EP00475 Leptophrys vorax TRINITY_DN2182_c0_g1_i1_p1
EP00295 Goniomonas pacifica CAMPEP_0175836132
EP00292 Cryptomonas paramecium CAMPEP_0113691932
Guillardia theta CCMP2712 XP_005829230
EP00289 Rhodomonas salina CAMPEP_0172111922
EP00762 Andalucia godoyi ANDGO_02527_mRNA_1
Euglena gracilis CAF05589
EP00668 Euglena longa GGOE01020724_1_p1
Naegleria lovaniensis XP_044544615
EP00671 Bodo saltans CUG88242_1
Trypanosoma brucei brucei TREU927 XP_829730
Leishmania major strain Friedlin XP_001685657
EP00656 Telonema subtile TRINITY_DN9965_c0_g1_i1_p1
EP00741 Cyanophora paradoxa tig00001376_g8540_t1
EP00274 Cyanoptyche gloeocystis CAMPEP_0196652572
EP00276 Gloeochaete wittrockiana CAMPEP_0184658996
Diacronema lutheri KAG8469706
Chrysochromulina tobinii KOO30081
Emiliania huxleyi CCMP1516 XP_005784168
EP00311 Isochrysis galbana CAMPEP_0179786518
EP00317 Phaeocystis antarctica CAMPEP_0198157978
EP00327 Prymnesium parvum CAMPEP_0184404202
Porphyridium purpureum KAA8496602
Galdieria sulphuraria XP_005707792
Cyanidioschyzon merolae strain 10D XP_005537162
EP00187 Rhodella violacea CAMPEP_0174912566
Chondrus crispus XP_005713748
Gracilariopsis chorda PXF41563
EP00750 Incisomonas marina INCI1157_2_p2
EP00753 Platysulcus tardus PLAT15847_1_p2
EP00647 Phytophthora sojae XP_009518458.1
Nannochloropsis gaditana EWM29624
EP00625 Aureococcus anophagefferens XP_009037037.1
EP00530 Phaeodactylum tricornutum XP_002178956.1
EP00609 Ectocarpus siliculosus CBN76663.1
Stentor coeruleus OMJ73155
EP00465 Bigelowiella longifila CAMPEP_0185268008
EP00469 Lotharella globosa CAMPEP_0167787606
EP00348 Stylonychia lemnae CDW75606.1
EP00348 Stylonychia lemnae CDW79133.1
EP00344 Euplotes crassus CAMPEP_0197007110
EP00333 Tetrahymena thermophila XP_001014580.2
EP00334 Paramecium tetraurelia XP_001460760.1
EP00744 Colponema vietnamica GILI01003513_1_p1
EP00451 Oxyrrhis marina CAMPEP_0204251920
Toxoplasma gondii ME49 XP_002367828
EP00387 Chromera velia CAMPEP_0184607814
EP00386 Alphamonas edax GDKI01038842_1_p1
EP00390 Vitrella brassicaformis CEM23213.1
PAPYR_5544 Paratrimastix pyriformis
Gene_5217 gnl Trimastix PCT 1613
Carpediemonas membranifera KAG9392848
gnl Ergobibamus cyprinoides 506
gnl Chilomastix cuspidata 1658
gnl Dysnectes brevis 2556_a3075_885
EP00704 Kipferlia bialata g4376_t1
EP00704 Kipferlia bialata g7933_t1
EP00764 Aduncisulcus paluster gnl Carplike NY0171_4551_a6191_273
Histomonas meleagridis KAH0790584
Tritrichomonas foetus OHS97529
Trichomonas vaginalis G3 XP_001330332
EP00705 Trichomonas vaginalis XP_001583420.1
EP00033 Pygsuia biforma GCRY01001327_1_p1
PAPYR_1328 Paratrimastix pyriformis
MONOS_1884 Monocercomonoides exilis
BLNAU_2741 Blattamonas nauphoetae
EP00032 Breviata anathema JZ550971_1_p1
AJE29366.1 Mastigamoeba balamuthi
Pelo_4471 Pelomyxa schiedti
c13979_g1_i1+c13979_g1_i4 Mastigella eilhardi
AJE29367.1 Mastigamoeba balamuthi
TRINITY_DN319752_c0_g1_i1 Mastigamoeba abducta
TRINITY_DN14673_c3_g1_i1 Endolimax sp.
Pelo_11253 Pelomyxa schiedti
c15747_g1_i1 Mastigella eilhardi
TRINITY_DN1519_c0_g1_i1 Rhizomastix vacuolata
TRINITY_DN556_c0_g1_i1 Rhizomastix libera
EP00161 Ancyromonas sigmoides TRINITY_DN14688_c0_g2_i1_p1
EP00004 Rigifila ramosa TRINITY_DN2738_c0_g1_i1_p1
EP00001 Collodictyon triciliatum 30319 3
EP00002 Diphylleia rotans TRINITY_DN1049_c0_g1_i1_p1
Capsaspora owczarzaki ATCC 30864 XP_004343819
EP00127 Corallochytrium limacisporum Clim evm49s11
Monosiga brevicollis MX1 XP_001750059
Ixodes scapularis XP_042147908
Adineta ricciae CAF1203093
EP00129 Allomyces macrogynus KNE62335.1
EP00157 Rozella allomycis EPZ33750.1
EP00134 Neurospora crassa XP_959535.2
EP00145 Schizosaccharomyces pombe NP_593496.1
EP00149 Cryptococcus neoformans XP_012049621.1
EP00153 Phycomyces blakesleeanus XP_018297760.1
EP00003 Mantamonas plastica TRINITY_DN383_c0_g1_i2_p1
EP00163 Fabomonas tropica TRINITY_DN9284_c0_g2_i1_p2
Planoprotostelium fungivorum PRP77981
EP00027 Filamoeba nolandi CAMPEP_0168551498
EP00027 Filamoeba nolandi CAMPEP_0168555482
EP00026 Physarum polycephalum Phypoly transcript_07750_p1
Acytostelium subglobosum LB1 XP_012752469
Polysphondylium violaceum KAF2068773
Dictyostelium discoideum AX4 XP_635122
Acytostelium subglobosum LB1 XP_012751200
Heterostelium album PN500 XP_020432723
Acanthamoeba castellanii str Neff XP_004352811
EP00005 Dracoamoeba jomungandri CAMPEP_0174250062
EP00028 Trichosphaerium sp. Am-I-7-wt CAMPEP_0168523434
EP00728 Arcella intermedia NDV31325.1
EP00029 Vermamoeba vermiformis TRINITY_DN6452_c0_g1_i1_p1
EP00029 Vermamoeba vermiformis TRINITY_DN9420_c0_g1_i1_p1
EP00013 Stygamoeba regulata CAMPEP_0177656996
EP00011 Vannella sp. DIVA3-517-6-12 CAMPEP_0114612290
EP00012 Vannella robusta CAMPEP_0206193112
EP00010 Vexillifera sp. DIVA3-564-2 CAMPEP_0201553696
EP00007 Cunea sp. BSH-02190019 CAMPEP_0174242488
EP00013 Stygamoeba regulata CAMPEP_0177655810
EP00008 Paramoeba atlantica CAMPEP_0201488222
EP00009 Paramoeba aestuarina CAMPEP_0201521176

F)

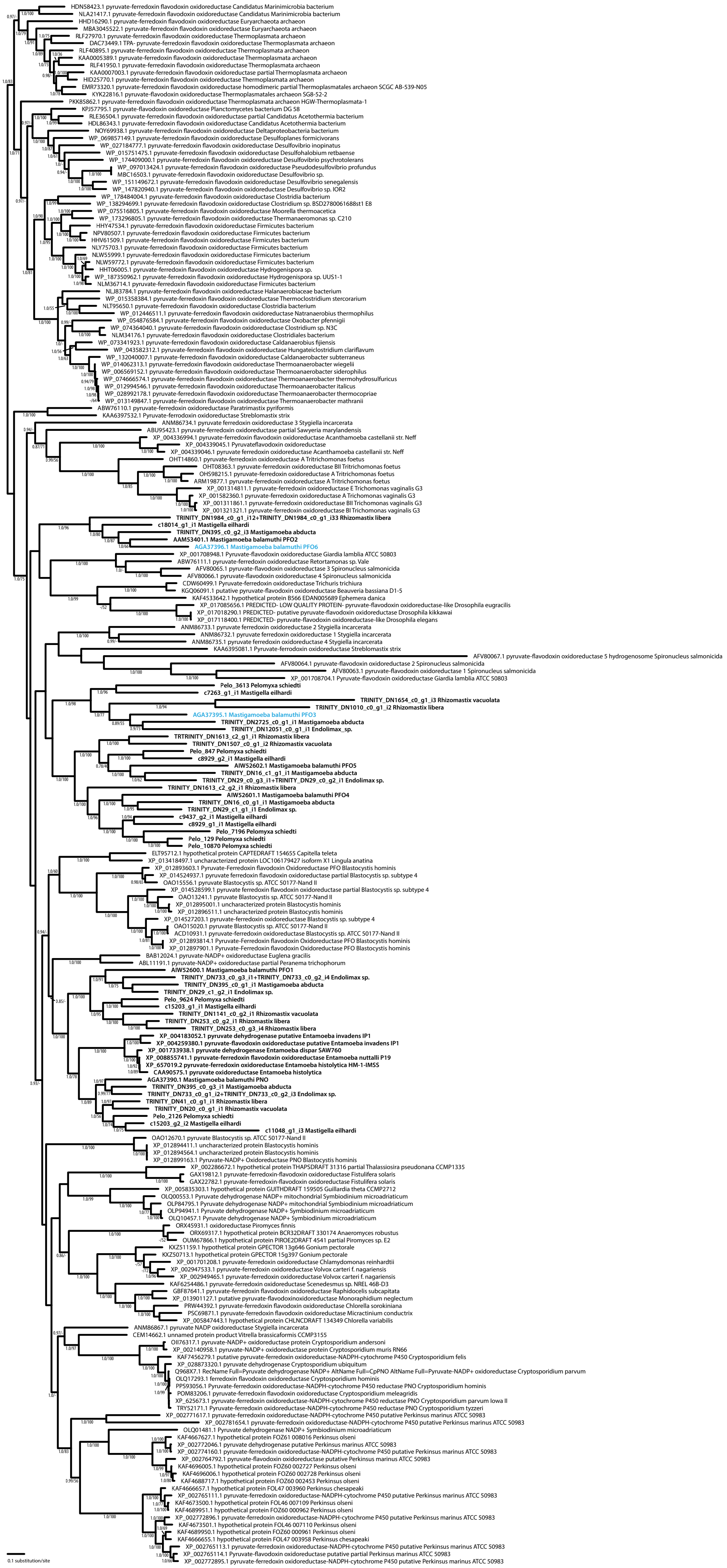

HDN58423.1 pyruvate-ferredoxin flavodoxin oxidoreductase Candidatus Marinimicrobia bacterium
NLA21417.1 pyruvate-ferredoxin flavodoxin oxidoreductase Candidatus Marinimicrobia bacterium
HHD16290.1 pyruvate-ferredoxin flavodoxin oxidoreductase Euryarchaeota archaeon
MBA3045522.1 pyruvate-ferredoxin flavodoxin oxidoreductase Euryarchaeota archaeon
RLF27970.1 pyruvate-ferredoxin flavodoxin oxidoreductase Thermoplasmata archaeon
DAC73449.1 TPA- pyruvate-ferredoxin flavodoxin oxidoreductase Thermoplasmata archaeon
RLF40895.1 pyruvate-ferredoxin flavodoxin oxidoreductase Thermoplasmata archaeon
KAA0005389.1 pyruvate-ferredoxin flavodoxin oxidoreductase Thermoplasmata archaeon
RLF41950.1 pyruvate-ferredoxin flavodoxin oxidoreductase Thermoplasmata archaeon
KAA0007003.1 pyruvate-ferredoxin flavodoxin oxidoreductase partial Thermoplasmata archaeon
HID25770.1 pyruvate-ferredoxin flavodoxin oxidoreductase Thermoplasmata archaeon
EMR73320.1 pyruvate-ferredoxin flavodoxin oxidoreductase homodimeric partial Thermoplasmatales archaeon SCGC AB-539-N05
KYK22816.1 pyruvate-flavodoxin oxidoreductase Thermoplasmatales archaeon SG8-52-2
PKK85862.1 pyruvate-ferredoxin flavodoxin oxidoreductase Thermoplasmata archaeon HGW-Thermoplasmata-1
KPJ57795.1 pyruvate-flavodoxin oxidoreductase Planctomycetes bacterium DG 58
RLE36504.1 pyruvate-ferredoxin flavodoxin oxidoreductase partial Candidatus Acetothermia bacterium
HDL86343.1 pyruvate-ferredoxin flavodoxin oxidoreductase Candidatus Acetothermia bacterium
NOY69938.1 pyruvate-ferredoxin flavodoxin oxidoreductase Deltaproteobacteria bacterium
WP_069857149.1 pyruvate-ferredoxin flavodoxin oxidoreductase Desulfoplanes formicivorans
WP_027184777.1 pyruvate-ferredoxin flavodoxin oxidoreductase Desulfovibrio inopinatus
WP_015751475.1 pyruvate-ferredoxin flavodoxin oxidoreductase Desulfohalobium retbaense
WP_174409000.1 pyruvate-ferredoxin flavodoxin oxidoreductase Desulfovibrio psychrotolerans
WP_097013424.1 pyruvate-ferredoxin flavodoxin oxidoreductase Pseudodesulfovibrio profundus
MBC16503.1 pyruvate-ferredoxin flavodoxin oxidoreductase Desulfovibrio sp.
WP_151149672.1 pyruvate-ferredoxin flavodoxin oxidoreductase Desulfovibrio senegalensis
WP_147820940.1 pyruvate-ferredoxin flavodoxin oxidoreductase Desulfovibrio sp. IOR2
WP_178484004.1 pyruvate-ferredoxin flavodoxin oxidoreductase Clostridia bacterium
WP_138294699.1 pyruvate-ferredoxin flavodoxin oxidoreductase Clostridium sp. BSD2780061688st1 E8
WP_075516805.1 pyruvate-ferredoxin flavodoxin oxidoreductase Moorella thermoacetica
WP_173296805.1 pyruvate-ferredoxin flavodoxin oxidoreductase Thermanaeromonas sp. C210
HHY47534.1 pyruvate-ferredoxin flavodoxin oxidoreductase Firmicutes bacterium
NPV80507.1 pyruvate-ferredoxin flavodoxin oxidoreductase Firmicutes bacterium
HHV61509.1 pyruvate-ferredoxin flavodoxin oxidoreductase Firmicutes bacterium
NLY75703.1 pyruvate-ferredoxin flavodoxin oxidoreductase Firmicutes bacterium
NLW55999.1 pyruvate-ferredoxin flavodoxin oxidoreductase Firmicutes bacterium
NLW59772.1 pyruvate-ferredoxin flavodoxin oxidoreductase Firmicutes bacterium
HHT06005.1 pyruvate-ferredoxin flavodoxin oxidoreductase Hydrogenispora sp.
WP_187350962.1 pyruvate-ferredoxin flavodoxin oxidoreductase Hydrogenispora sp. UUS1-1
NLM36714.1 pyruvate-ferredoxin flavodoxin oxidoreductase Firmicutes bacterium
NLJ83784.1 pyruvate-ferredoxin flavodoxin oxidoreductase Halanaerobiaceae bacterium
WP_015358384.1 pyruvate-ferredoxin flavodoxin oxidoreductase Thermoclostridium stercorarium
NLT95650.1 pyruvate-ferredoxin flavodoxin oxidoreductase Clostridia bacterium
WP_012446511.1 pyruvate-ferredoxin flavodoxin oxidoreductase Natranaerobius thermophilus
WP_054876584.1 pyruvate-ferredoxin flavodoxin oxidoreductase Oxobacter pfennigii
WP_074364040.1 pyruvate-ferredoxin flavodoxin oxidoreductase Clostridium sp. N3C
NLM34176.1 pyruvate-ferredoxin flavodoxin oxidoreductase Clostridiales bacterium
WP_073341923.1 pyruvate-ferredoxin flavodoxin oxidoreductase Caldanaerobius fijiensis
WP_043582312.1 pyruvate-ferredoxin flavodoxin oxidoreductase Hungateiclostridium clariflavum
WP_132040007.1 pyruvate-ferredoxin flavodoxin oxidoreductase Caldanaerobacter subterraneus
WP_014062313.1 pyruvate-ferredoxin flavodoxin oxidoreductase Thermoanaerobacter wiegelii
WP_006569152.1 pyruvate-ferredoxin flavodoxin oxidoreductase Thermoanaerobacter siderophilus
WP_074666574.1 pyruvate-ferredoxin flavodoxin oxidoreductase Thermoanaerobacter thermohydrosulfuricus
WP_012994546.1 pyruvate-ferredoxin flavodoxin oxidoreductase Thermoanaerobacter italicus
WP_028992178.1 pyruvate-ferredoxin flavodoxin oxidoreductase Thermoanaerobacter thermocopriae
WP_013149847.1 pyruvate-ferredoxin flavodoxin oxidoreductase Thermoanaerobacter mathranii
ABW76110.1 pyruvate-ferredoxin oxidoreductase Paratrimastix pyriformis
KAA6397532.1 Pyruvate-ferrodoxin oxidoreductase Streblomastix strix
ANM86734.1 pyruvate ferredoxin oxidoreductase 3 Stygiella incarcerata
ABU95423.1 pyruvate-ferredoxin oxidoreductase partial Sawyeria marylandensis
XP_004336994.1 pyruvate-ferredoxin flavodoxin oxidoreductase Acanthamoeba castellanii str. Neff
XP_004339045.1 Pyruvateflavodoxin oxidoreductase
XP_004339046.1 pyruvate ferredoxin oxidoreductase Acanthamoeba castellanii str. Neff
OHT14860.1 pyruvate-ferredoxin oxidoreductase A Tritrichomonas foetus
OHT08363.1 pyruvate-ferredoxin oxidoreductase BII Tritrichomonas foetus
OHS98215.1 pyruvate-ferredoxin oxidoreductase A Tritrichomonas foetus
ARM19877.1 pyruvate-ferredoxin oxidoreductase A Tritrichomonas foetus
XP_001314811.1 pyruvate-ferredoxin oxidoreductase E Trichomonas vaginalis G3
XP_001582360.1 pyruvate-ferredoxin oxidoreductase A Trichomonas vaginalis G3
XP_001311861.1 pyruvate-ferredoxin oxidoreductase BII Trichomonas vaginalis G3
XP_001321321.1 pyruvate-ferredoxin oxidoreductase BI Trichomonas vaginalis G3
TRINITY_DN1984_c0_g1_i12+TRINITY_DN1984_c0_g1_i33 Rhizomastix libera
c18014_g1_i1 Mastigella eilhardi
TRINITY_DN395_c0_g2_i3 Mastigamoeba abducta
AAM53401.1 Mastigamoeba balamuthi PFO2
AGA37396.1 Mastigamoeba balamuthi PFO6
XP_001708948.1 Pyruvate-flavodoxin oxidoreductase Giardia lamblia ATCC 50803
ABW76111.1 pyruvate-ferredoxin oxidoreductase Retortamonas sp. Vale
AFV80065.1 pyruvate-flavodoxin oxidoreductase 3 Spironucleus salmonicida
AFV80066.1 pyruvate-flavodoxin oxidoreductase 4 Spironucleus salmonicida
CDW60499.1 Pyruvate-ferredoxin oxidoreductase Trichuris trichiura
KGQ06091.1 putative pyruvate-flavodoxin oxidoreductase Beauveria bassiana D1-5
KAF4533642.1 hypothetical protein B566 EDAN005689 Ephemera danica
XP_017085656.1 PREDICTED- LOW QUALITY PROTEIN- pyruvate-flavodoxin oxidoreductase-like Drosophila eugracilis
XP_017018290.1 PREDICTED- putative pyruvate-flavodoxin oxidoreductase Drosophila kikkawai
XP_017118400.1 PREDICTED- pyruvate-flavodoxin oxidoreductase-like Drosophila elegans
ANM86733.1 pyruvate ferredoxin oxidoreductase 2 Stygiella incarcerata
ANM86732.1 pyruvate ferredoxin oxidoreductase 1 Stygiella incarcerata
ANM86735.1 pyruvate ferredoxin oxidoreductase 4 Stygiella incarcerata
KAA6395081.1 Pyruvate-ferrodoxin oxidoreductase Streblomastix strix
AFV80067.1 pyruvate-flavodoxin oxidoreductase 5 hydrogenosome Spironucleus salmonicida
AFV80064.1 pyruvate-flavodoxin oxidoreductase 2 Spironucleus salmonicida
AFV80063.1 pyruvate-flavodoxin oxidoreductase 1 Spironucleus salmonicida
XP_001708704.1 Pyruvate-flavodoxin oxidoreductase Giardia lamblia ATCC 50803
Pelo_3613 Pelomyxa schiedti
c7263_g1_i1 Mastigella eilhardi
TRINITY_DN1654_c0_g1_i3 Rhizomastix vacuolata
TRINITY_DN1010_c0_g1_i2 Rhizomastix libera
AGA37395.1 Mastigamoeba balamuthi PFO3
TRINITY_DN2725_c0_g1_i1 Mastigamoeba abducta
TRINITY_DN12051_c0_g1_i1 Endolimax_sp.
TRTRINITY_DN1613_c2_g1_i1 Rhizomastix libera
TRINITY_DN1507_c0_g1_i2 Rhizomastix vacuolata
Pelo_847 Pelomyxa schiedti
c8929_g2_i1 Mastigella eilhardi
AIW52602.1 Mastigamoeba balamuthi PFO5
TRINITY_DN16_c1_g1_i1 Mastigamoeba abducta
TRINITY_DN29_c0_g3_i1+TRINITY_DN29_c0_g2_i1 Endolimax sp.
TRINITY_DN1613_c2_g2_i1 Rhizomastix libera
AIW52601.1 Mastigamoeba balamuthi PFO4
TRINITY_DN16_c0_g1_i1 Mastigamoeba abducta
TRINITY_DN29_c1_g1_i1 Endolimax sp.
c9437_g2_i1 Mastigella eilhardi
c8929_g1_i1 Mastigella eilhardi
Pelo_7196 Pelomyxa schiedti
Pelo_129 Pelomyxa schiedti
Pelo_10870 Pelomyxa schiedti
ELT95712.1 hypothetical protein CAPTEDRAFT 154655 Capitella teleta
XP_013418497.1 uncharacterized protein LOC106179427 isoform X1 Lingula anatina
XP_012893603.1 Pyruvate-Ferredoxin flavodoxin Oxidoreductase PFO Blastocystis hominis
XP_014524937.1 pyruvate ferredoxin flavodoxin oxidoreductase partial Blastocystis sp. subtype 4
OAO15556.1 pyruvate Blastocystis sp. ATCC 50177-Nand II
XP_014528599.1 pyruvate ferredoxin flavodoxin oxidoreductase partial Blastocystis sp. subtype 4
OAO13241.1 pyruvate Blastocystis sp. ATCC 50177-Nand II
XP_012895001.1 uncharacterized protein Blastocystis hominis
XP_012896511.1 uncharacterized protein Blastocystis hominis
XP_014527203.1 pyruvate-ferredoxin oxidoreductase Blastocystis sp. subtype 4
OAO15020.1 pyruvate Blastocystis sp. ATCC 50177-Nand II
ACD10931.1 pyruvate-ferredoxin oxidoreductase Blastocystis sp. ATCC 50177-Nand II
XP_012893814.1 Pyruvate-Ferredoxin flavodoxin Oxidoreductase PFO Blastocystis hominis
XP_012897901.1 Pyruvate-Ferredoxin flavodoxin Oxidoreductase PFO Blastocystis hominis
BAB12024.1 pyruvate-NADP+ oxidoreductase Euglena gracilis
ABL11191.1 pyruvate-NADP+ oxidoreductase partial Peranema trichophorum
AIW52600.1 Mastigamoeba balamuthi PFO1
TRINITY_DN733_c0_g3_i1+TRINITY_DN733_c0_g2_i4 Endolimax sp.
TRINITY_DN395_c0_g1_i1 Mastigamoeba abducta
TRINITY_DN29_c1_g2_i1 Endolimax sp.
Pelo_9624 Pelomyxa schiedti
c15203_g1_i1 Mastigella eilhardi
TRINITY_DN1141_c0_g2_i1 Rhizomastix vacuolata
TRINITY_DN253_c0_g2_i1 Rhizomastix libera
TRINITY_DN253_c0_g3_i4 Rhizomastix libera
XP_004183052.1 pyruvate dehydrogenase putative Entamoeba invadens IP1
XP_004259380.1 pyruvate-flavodoxin oxidoreductase putative Entamoeba invadens IP1
XP_001733938.1 pyruvate dehydrogenase Entamoeba dispar SAW760
XP_008855741.1 pyruvate-ferredoxin flavodoxin oxidoreductase Entamoeba nuttalli P19
XP_657019.2 pyruvate-ferredoxin oxidoreductase Entamoeba histolytica HM-1-IMSS
CAA90575.1 pyruvate oxidoreductase Entamoeba histolytica
AGA37390.1 Mastigamoeba balamuthi PNO
TRINITY_DN395_c0_g3_i1 Mastigamoeba abducta
TRINITY_DN733_c0_g1_i2+TRINITY_DN733_c0_g2_i3 Endolimax sp.
TRINITY_DN41_c0_g1_i1 Rhizomastix libera
TRINITY_DN20_c0_g1_i1 Rhizomastix vacuolata
Pelo_2126 Pelomyxa schiedti
c15203_g2_i2 Mastigella eilhardi
c11048_g1_i3 Mastigella eilhardi
OAO12670.1 pyruvate Blastocystis sp. ATCC 50177-Nand II
XP_012894411.1 uncharacterized protein Blastocystis hominis
XP_012894564.1 uncharacterized protein Blastocystis hominis
XP_012899163.1 Pyruvate-NADP+ Oxidoreductase PNO Blastocystis hominis
XP_002286672.1 hypothetical protein THAPSDRAFT 31316 partial Thalassiosira pseudonana CCMP1335
GAX19812.1 pyruvate-ferredoxin-flavodoxin oxidoreductase Fistulifera solaris
GAX22782.1 pyruvate-ferredoxin-flavodoxin oxidoreductase Fistulifera solaris
XP_005835303.1 hypothetical protein GUITHDRAFT 159505 Guillardia theta CCMP2712
OLQ00553.1 Pyruvate dehydrogenase NADP+ mitochondrial Symbiodinium microadriaticum
OLP84795.1 Pyruvate dehydrogenase NADP+ mitochondrial Symbiodinium microadriaticum
OLP94941.1 Pyruvate dehydrogenase NADP+ Symbiodinium microadriaticum
OLQ10457.1 Pyruvate dehydrogenase NADP+ Symbiodinium microadriaticum
ORX45931.1 oxidoreductase Piromyces finnis
ORX69317.1 hypothetical protein BCR32DRAFT 330174 Anaeromyces robustus
OUM67866.1 hypothetical protein PIROE2DRAFT 4541 partial Piromyces sp. E2
KXZ51159.1 hypothetical protein GPECTOR 13g646 Gonium pectorale
KXZ50713.1 hypothetical protein GPECTOR 15g397 Gonium pectorale
XP_001701208.1 pyruvate-ferredoxin oxidoreductase Chlamydomonas reinhardtii
XP_002947533.1 pyruvate-ferredoxin oxidoreductase Volvox carteri f. nagariensis
XP_002949465.1 pyruvate-ferredoxin oxidoreductase Volvox carteri f. nagariensis
KAF6254486.1 pyruvate-ferredoxin oxidoreductase Scenedesmus sp. NREL 46B-D3
GBF87641.1 pyruvate-ferredoxin flavodoxin oxidoreductase Raphidocelis subcapitata
XP_013901127.1 putative pyruvate-flavodoxinoxidoreductase Monoraphidium neglectum
PRW44392.1 pyruvate-ferredoxin flavodoxin oxidoreductase Chlorella sorokiniana
PSC69871.1 pyruvate-ferredoxin flavodoxin oxidoreductase Micractinium conductrix
XP_005847443.1 hypothetical protein CHLNCDRAFT 134349 Chlorella variabilis
ANM86867.1 pyruvate NADP oxidoreductase Stygiella incarcerata
CEM14662.1 unnamed protein product Vitrella brassicaformis CCMP3155
OII76317.1 pyruvate-NADP+ oxidoreductase protein Cryptosporidium andersoni
XP_002140958.1 pyruvate-NADP+ oxidoreductase protein Cryptosporidium muris RN66
KAF7456279.1 putative pyruvate-ferredoxin oxidoreductase-NADPH-cytochrome P450 Cryptosporidium felis
XP_028873320.1 pyruvate dehydrogenase Cryptosporidium ubiquitum
Q968X7.1 RecName Full=Pyruvate dehydrogenase NADP+ AltName Full=CpPNO AltName Full=Pyruvate-NADP+ oxidoreductase Cryptosporidium parvum
OLQ17293.1 ferredoxin flavodoxin oxidoreductase Cryptosporidium hominis
PPS93056.1 Pyruvate-ferredoxin oxidoreductase-NADPH-cytochrome P450 reductase PNO Cryptosporidium hominis
POM83206.1 pyruvate-ferredoxin flavodoxin oxidoreductase Cryptosporidium meleagridis
XP_625673.1 pyruvate-ferredoxin oxidoreductase-NADPH-cytochrome P450 reductase PNO Cryptosporidium parvum Iowa II
TRY52171.1 Pyruvate-ferredoxin oxidoreductase-NADPH-cytochrome P450 reductase PNO Cryptosporidium tyzzeri
XP_002771617.1 pyruvate-ferredoxin oxidoreductase-NADPH-cytochrome P450 putative Perkinsus marinus ATCC 50983
XP_002781654.1 pyruvate-ferredoxin oxidoreductase-NADPH-cytochrome P450 putative Perkinsus marinus ATCC 50983
OLQ01481.1 Pyruvate dehydrogenase NADP+ Symbiodinium microadriaticum
KAF4667627.1 hypothetical protein FOZ61 008016 Perkinsus olseni
XP_002772046.1 pyruvate dehydrogenase putative Perkinsus marinus ATCC 50983
XP_002774160.1 pyruvate-ferredoxin oxidoreductase-NADPH-cytochrome P450 putative Perkinsus marinus ATCC 50983
XP_002764792.1 pyruvate dehydrogenase putative Perkinsus marinus ATCC 50983
KAF4696005.1 hypothetical protein FOZ60 002727 Perkinsus olseni
KAF4696006.1 hypothetical protein FOZ60 002728 Perkinsus olseni
KAF4688717.1 hypothetical protein FOZ60 002453 Perkinsus olseni
KAF4666657.1 hypothetical protein FOL47 003960 Perkinsus chesapeaki
XP_002765111.1 pyruvate-ferredoxin oxidoreductase-NADPH-cytochrome P450 putative Perkinsus marinus ATCC 50983
KAF4673500.1 hypothetical protein FOL46 007109 Perkinsus olseni
KAF4689951.1 hypothetical protein FOZ60 000962 Perkinsus olseni
XP_002772896.1 pyruvate-ferredoxin oxidoreductase-NADPH-cytochrome P450 putative Perkinsus marinus ATCC 50983
KAF4673501.1 hypothetical protein FOL46 007110 Perkinsus olseni
KAF4689950.1 hypothetical protein FOZ60 000961 Perkinsus olseni
KAF4666655.1 hypothetical protein FOL47 003958 Perkinsus chesapeaki
XP_002765113.1 pyruvate-ferredoxin oxidoreductase-NADPH-cytochrome P450 putative Perkinsus marinus ATCC 50983
XP_002765114.1 Pyruvate-ferredoxin oxidoreductase putative partial Perkinsus marinus ATCC 50983
XP_002772895.1 pyruvate-ferredoxin oxidoreductase-NADPH-cytochrome P450 putative Perkinsus marinus ATCC 50983
0.1 substitution/site

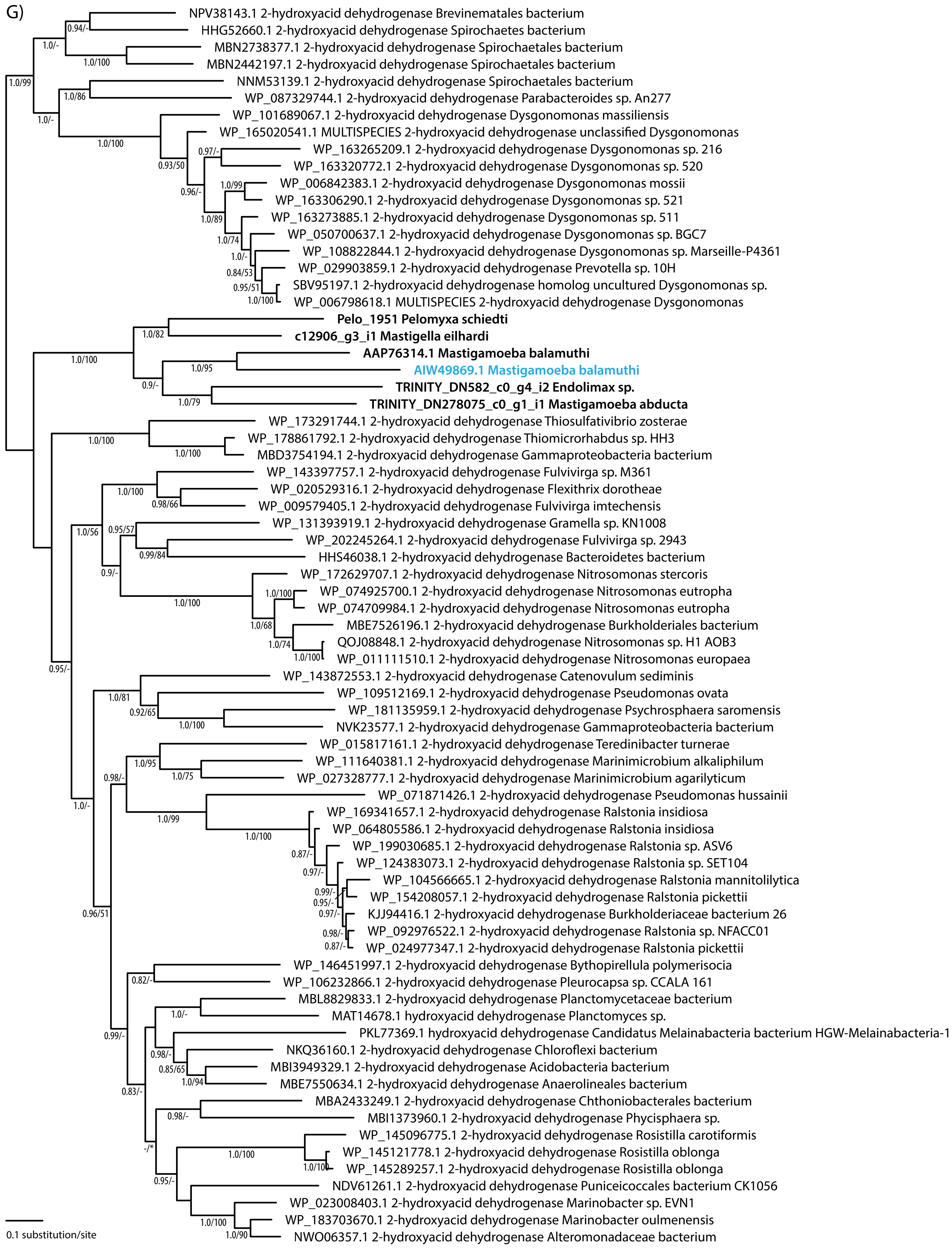
G)
NPV38143.1 2-hydroxyacid dehydrogenase Brevinematales bacterium
HHG52660.1 2-hydroxyacid dehydrogenase Spirochaetes bacterium
MBN2738377.1 2-hydroxyacid dehydrogenase Spirochaetales bacterium
MBN2442197.1 2-hydroxyacid dehydrogenase Spirochaetales bacterium
NNM53139.1 2-hydroxyacid dehydrogenase Spirochaetales bacterium
WP_087329744.1 2-hydroxyacid dehydrogenase Parabacteroides sp. An277
WP_101689067.1 2-hydroxyacid dehydrogenase Dysgonomonas massiliensis
WP_165020541.1 MULTISPECIES 2-hydroxyacid dehydrogenase unclassified Dysgonomonas
WP_163265209.1 2-hydroxyacid dehydrogenase Dysgonomonas sp. 216
WP_163320772.1 2-hydroxyacid dehydrogenase Dysgonomonas sp. 520
WP_006842383.1 2-hydroxyacid dehydrogenase Dysgonomonas mossii
WP_163306290.1 2-hydroxyacid dehydrogenase Dysgonomonas sp. 521
WP_163273885.1 2-hydroxyacid dehydrogenase Dysgonomonas sp. 511
WP_050700637.1 2-hydroxyacid dehydrogenase Dysgonomonas sp. BGC7
WP_108822844.1 2-hydroxyacid dehydrogenase Dysgonomonas sp. Marseille-P4361
WP_029903859.1 2-hydroxyacid dehydrogenase Prevotella sp. 10H
SBV95197.1 2-hydroxyacid dehydrogenase homolog uncultured Dysgonomonas sp.
WP_006798618.1 MULTISPECIES 2-hydroxyacid dehydrogenase Dysgonomonas
Pelo_1951 Pelomyxa schiedti
c12906_g3_i1 Mastigella eilhardi
AAP76314.1 Mastigamoeba balamuthi
AIW49869.1 Mastigamoeba balamuthi
TRINITY_DN582_c0_g4_i2 Endolimax sp.
TRINITY_DN278075_c0_g1_i1 Mastigamoeba abducta
WP_173291744.1 2-hydroxyacid dehydrogenase Thiosulfativibrio zosterae
WP_178861792.1 2-hydroxyacid dehydrogenase Thiomicrorhabdus sp. HH3
MBD3754194.1 2-hydroxyacid dehydrogenase Gammaproteobacteria bacterium
WP_143397757.1 2-hydroxyacid dehydrogenase Fulvivirga sp. M361
WP_020529316.1 2-hydroxyacid dehydrogenase Flexithrix dorotheae
WP_009579405.1 2-hydroxyacid dehydrogenase Fulvivirga imtechensis
WP_131393919.1 2-hydroxyacid dehydrogenase Gramella sp. KN1008
WP_202245264.1 2-hydroxyacid dehydrogenase Fulvivirga sp. 2943
HHS46038.1 2-hydroxyacid dehydrogenase Bacteroidetes bacterium
WP_172629707.1 2-hydroxyacid dehydrogenase Nitrosomonas stercoris
WP_074925700.1 2-hydroxyacid dehydrogenase Nitrosomonas eutropha
WP_074709984.1 2-hydroxyacid dehydrogenase Nitrosomonas eutropha
MBE7526196.1 2-hydroxyacid dehydrogenase Burkholderiales bacterium
QOJ08848.1 2-hydroxyacid dehydrogenase Nitrosomonas sp. H1 AOB3
WP_011111510.1 2-hydroxyacid dehydrogenase Nitrosomonas europaea
WP_143872553.1 2-hydroxyacid dehydrogenase Catenovulum sediminis
WP_109512169.1 2-hydroxyacid dehydrogenase Pseudomonas ovata
WP_181135959.1 2-hydroxyacid dehydrogenase Psychrosphaera saromensis
NVK23577.1 2-hydroxyacid dehydrogenase Gammaproteobacteria bacterium
WP_015817161.1 2-hydroxyacid dehydrogenase Teredinibacter turnerae
WP_111640381.1 2-hydroxyacid dehydrogenase Marinimicrobium alkaliphilum
WP_027328777.1 2-hydroxyacid dehydrogenase Marinimicrobium agarilyticum
WP_071871426.1 2-hydroxyacid dehydrogenase Pseudomonas hussainii
WP_169341657.1 2-hydroxyacid dehydrogenase Ralstonia insidiosa
WP_064805586.1 2-hydroxyacid dehydrogenase Ralstonia insidiosa
WP_199030685.1 2-hydroxyacid dehydrogenase Ralstonia sp. ASV6
WP_124383073.1 2-hydroxyacid dehydrogenase Ralstonia sp. SET104
WP_104566665.1 2-hydroxyacid dehydrogenase Ralstonia mannitolilytica
WP_154208057.1 2-hydroxyacid dehydrogenase Ralstonia pickettii
KJJ94416.1 2-hydroxyacid dehydrogenase Burkholderiaceae bacterium 26
WP_092976522.1 2-hydroxyacid dehydrogenase Ralstonia sp. NFACC01
WP_024977347.1 2-hydroxyacid dehydrogenase Ralstonia pickettii
WP_146451997.1 2-hydroxyacid dehydrogenase Bythopirellula polymerisocia
WP_106232866.1 2-hydroxyacid dehydrogenase Pleurocapsa sp. CCALA 161
MBL8829833.1 2-hydroxyacid dehydrogenase Planctomycetaceae bacterium
MAT14678.1 hydroxyacid dehydrogenase Planctomyces sp.
PKL77369.1 hydroxyacid dehydrogenase Candidatus Melainabacteria bacterium HGW-Melainabacteria-1
NKQ36160.1 2-hydroxyacid dehydrogenase Chloroflexi bacterium
MBI3949329.1 2-hydroxyacid dehydrogenase Acidobacteria bacterium
MBE7550634.1 2-hydroxyacid dehydrogenase Anaerolineales bacterium
MBA2433249.1 2-hydroxyacid dehydrogenase Chthoniobacterales bacterium
MBI1373960.1 2-hydroxyacid dehydrogenase Phycisphaera sp.
WP_145096775.1 2-hydroxyacid dehydrogenase Rosistilla carotiformis
WP_145121778.1 2-hydroxyacid dehydrogenase Rosistilla oblonga
WP_145289257.1 2-hydroxyacid dehydrogenase Rosistilla oblonga
NDV61261.1 2-hydroxyacid dehydrogenase Puniceicoccales bacterium CK1056
WP_023008403.1 2-hydroxyacid dehydrogenase Marinobacter sp. EVN1
WP_183703670.1 2-hydroxyacid dehydrogenase Marinobacter oulmenensis
NWO06357.1 2-hydroxyacid dehydrogenase Alteromonadaceae bacterium
0.1 substitution/site

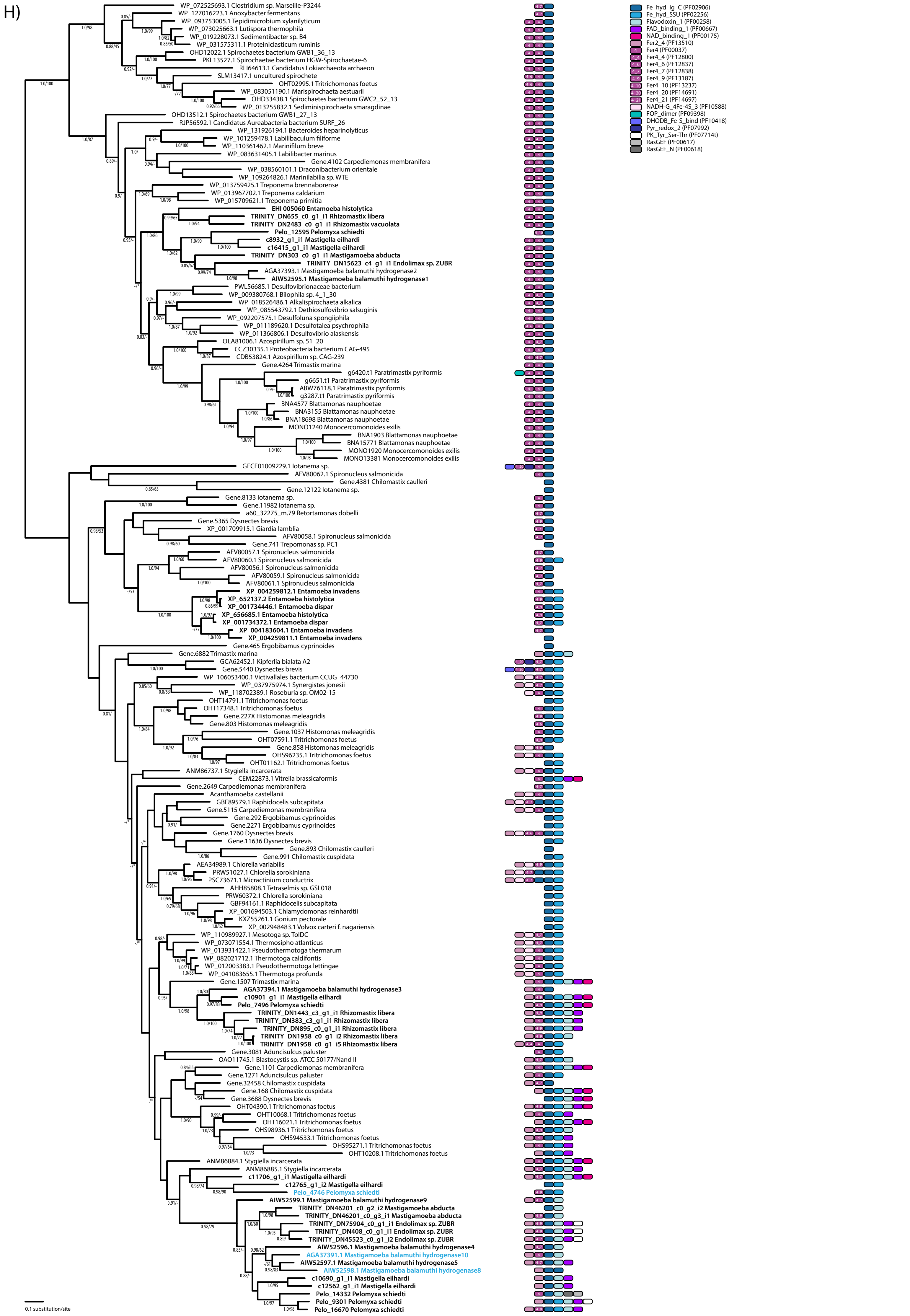
H)
WP_072525693.1 Clostridium sp. Marseille-P3244
WP_127016223.1 Anoxybacter fermentans
WP_093753005.1 Tepidimicrobium xylanilyticum
WP_073025663.1 Lutispora thermophila
WP_019228073.1 Sedimentibacter sp. B4
WP_031575311.1 Proteiniclasticum ruminis
OHD12022.1 Spirochaetes bacterium GWB1_36_13
PKL13527.1 Spirochaetae bacterium HGW-Spirochaetae-6
RLI64613.1 Candidatus Lokiarchaeota archaeon
SLM13417.1 uncultured spirochete
OHT02995.1 Tritrichomonas foetus
WP_083051190.1 Marispirochaeta aestuarii
OHD33438.1 Spirochaetes bacterium GWC2_52_13
WP_013255832.1 Sediminispirochaeta smaragdinae
OHD13512.1 Spirochaetes bacterium GWB1_27_13
RJP56592.1 Candidatus Aureabacteria bacterium SURF_26
WP_131926194.1 Bacteroides heparinolyticus
WP_101259478.1 Labilibaculum filiforme
WP_110361462.1 Marinifilum breve
WP_083631405.1 Labilibacter marinus
Gene.4102 Carpediemonas membranifera
WP_038560101.1 Draconibacterium orientale
WP_109264826.1 Marinilabilia sp. WTE
WP_013759425.1 Treponema brennaborense
WP_013967702.1 Treponema caldarium
WP_015709621.1 Treponema primitia
EHI 005060 Entamoeba histolytica
TRINITY_DN655_c0_g1_i1 Rhizomastix libera
TRINITY_DN2483_c0_g1_i1 Rhizomastix vacuolata
Pelo_12595 Pelomyxa schiedti
c8932_g1_i1 Mastigella eilhardi
c16415_g1_i1 Mastigella eilhardi
TRINITY_DN303_c0_g1_i1 Mastigamoeba abducta
TRINITY_DN15623_c4_g1_i1 Endolimax sp. ZUBR
AGA37393.1 Mastigamoeba balamuthi hydrogenase2
AIW52595.1 Mastigamoeba balamuthi hydrogenase1
PWL56685.1 Desulfovibrionaceae bacterium
WP_009380768.1 Bilophila sp. 4_1_30
WP_018526486.1 Alkalispirochaeta alkalica
WP_085543792.1 Dethiosulfovibrio salsuginis
WP_092207575.1 Desulfoluna spongiiphila
WP_011189620.1 Desulfotalea psychrophila
WP_011366806.1 Desulfovibrio alaskensis
OLA81006.1 Azospirillum sp. 51_20
CCZ30335.1 Proteobacteria bacterium CAG-495
CDB53824.1 Azospirillum sp. CAG-239
Gene.4264 Trimastix marina
g6420.t1 Paratrimastix pyriformis
g6651.t1 Paratrimastix pyriformis
ABW76118.1 Paratrimastix pyriformis
g3287.t1 Paratrimastix pyriformis
BNA4577 Blattamonas nauphoetae
BNA3155 Blattamonas nauphoetae
BNA18698 Blattamonas nauphoetae
MONO1240 Monocercomonoides exilis
BNA1903 Blattamonas nauphoetae
BNA15771 Blattamonas nauphoetae
MONO1920 Monocercomonoides exilis
MONO13381 Monocercomonoides exilis
GFCE01009229.1 Iotanema sp.
AFV80062.1 Spironucleus salmonicida
Gene.4381 Chilomastix caulleri
Gene.12122 Iotanema sp.
Gene.8133 Iotanema sp.
Gene.11982 Iotanema sp.
a60_32275_m.79 Retortamonas dobelli
Gene.5365 Dysnectes brevis
XP_001709915.1 Giardia lamblia
AFV80058.1 Spironucleus salmonicida
Gene.741 Trepomonas sp. PC1
AFV80057.1 Spironucleus salmonicida
AFV80060.1 Spironucleus salmonicida
AFV80056.1 Spironucleus salmonicida
AFV80059.1 Spironucleus salmonicida
AFV80061.1 Spironucleus salmonicida
XP_004259812.1 Entamoeba invadens
XP_652137.2 Entamoeba histolytica
XP_001734446.1 Entamoeba dispar
XP_656685.1 Entamoeba histolytica
XP_001734372.1 Entamoeba dispar
XP_004183604.1 Entamoeba invadens
XP_004259811.1 Entamoeba invadens
Gene.465 Ergobibamus cyprinoides
Gene.6882 Trimastix marina
GCA62452.1 Kipferlia bialata A2
Gene.5440 Dysnectes brevis
WP_106053400.1 Victivallales bacterium CCUG_44730
WP_037975974.1 Synergistes jonesii
WP_118702389.1 Roseburia sp. OM02-15
OHT14791.1 Tritrichomonas foetus
OHT17348.1 Tritrichomonas foetus
Gene.227X Histomonas meleagridis
Gene.803 Histomonas meleagridis
Gene.1037 Histomonas meleagridis
OHT07591.1 Tritrichomonas foetus
Gene.858 Histomonas meleagridis
OHS96235.1 Tritrichomonas foetus
OHT01162.1 Tritrichomonas foetus
ANM86737.1 Stygiella incarcerata
CEM22873.1 Vitrella brassicaformis
Gene.2649 Carpediemonas membranifera
Acanthamoeba castellanii
GBF89579.1 Raphidocelis subcapitata
Gene.5115 Carpediemonas membranifera
Gene.292 Ergobibamus cyprinoides
Gene.2271 Ergobibamus cyprinoides
Gene.1760 Dysnectes brevis
Gene.11636 Dysnectes brevis
Gene.893 Chilomastix caulleri
Gene.991 Chilomastix cuspidata
AEA34989.1 Chlorella variabilis
PRW51027.1 Chlorella sorokiniana
PSC73671.1 Micractinium conductrix
AHH85808.1 Tetraselmis sp. GSL018
PRW60372.1 Chlorella sorokiniana
GBF94161.1 Raphidocelis subcapitata
XP_001694503.1 Chlamydomonas reinhardtii
KXZ55261.1 Gonium pectorale
XP_002948483.1 Volvox carteri f. nagariensis
WP_110989927.1 Mesotoga sp. TolDC
WP_073071554.1 Thermosipho atlanticus
WP_013931422.1 Pseudothermotoga thermarum
WP_082021712.1 Thermotoga caldifontis
WP_012003383.1 Pseudothermotoga lettingae
WP_041083655.1 Thermotoga profunda
Gene.1507 Trimastix marina
AGA37394.1 Mastigamoeba balamuthi hydrogenase3
c10901_g1_i1 Mastigella eilhardi
Pelo_7496 Pelomyxa schiedti
TRINITY_DN1443_c3_g1_i1 Rhizomastix libera
TRINITY_DN383_c3_g1_i1 Rhizomastix libera
TRINITY_DN895_c0_g1_i1 Rhizomastix libera
TRINITY_DN1958_c0_g1_i2 Rhizomastix libera
TRINITY_DN1958_c0_g1_i5 Rhizomastix libera
Gene.3081 Aduncisulcus paluster
OAO11745.1 Blastocystis sp. ATCC 50177/Nand II
Gene.1101 Carpediemonas membranifera
Gene.1271 Aduncisulcus paluster
Gene.32458 Chilomastix cuspidata
Gene.168 Chilomastix cuspidata
Gene.3688 Dysnectes brevis
OHT04390.1 Tritrichomonas foetus
OHT10068.1 Tritrichomonas foetus
OHT16021.1 Tritrichomonas foetus
OHS98936.1 Tritrichomonas foetus
OHS94533.1 Tritrichomonas foetus
OHS95271.1 Tritrichomonas foetus
OHT10208.1 Tritrichomonas foetus
ANM86884.1 Stygiella incarcerata
ANM86885.1 Stygiella incarcerata
c11706_g1_i1 Mastigella eilhardi
c12765_g1_i2 Mastigella eilhardi
Pelo_4746 Pelomyxa schiedti
AIW52599.1 Mastigamoeba balamuthi hydrogenase9
TRINITY_DN46201_c0_g2_i2 Mastigamoeba abducta
TRINITY_DN46201_c0_g3_i1 Mastigamoeba abducta
TRINITY_DN75904_c0_g1_i1 Endolimax sp. ZUBR
TRINITY_DN408_c0_g1_i1 Endolimax sp. ZUBR
TRINITY_DN45523_c0_g1_i2 Endolimax sp. ZUBR
AIW52596.1 Mastigamoeba balamuthi hydrogenase4
AGA37391.1 Mastigamoeba balamuthi hydrogenase10
AIW52597.1 Mastigamoeba balamuthi hydrogenase5
AIW52598.1 Mastigamoeba balamuthi hydrogenase8
c10690_g1_i1 Mastigella eilhardi
c12562_g1_i1 Mastigella eilhardi
Pelo_14332 Pelomyxa schiedti
Pelo_9301 Pelomyxa schiedti
Pelo_16670 Pelomyxa schiedti
0.1 substitution/site
Fe_hyd_lg_C (PF02906)
Fe_hyd_SSU (PF02256)
Flavodoxin_1 (PF00258)
FAD_binding_1 (PF00667)
NAD_binding_1 (PF00175)
Fer2_4 (PF13510)
Fer4 (PF00037)
Fer4_4 (PF12800)
Fer4_6 (PF12837)
Fer4_7 (PF12838)
Fer4_9 (PF13187)
Fer4_10 (PF13237)
Fer4_20 (PF14691)
Fer4_21 (PF14697)
NADH-G_4Fe-4S_3 (PF10588)
FOP_dimer (PF09398)
DHODB_Fe-S_bind (PF10418)
Pyr_redox_2 (PF07992)
PK_Tyr_Ser-Thr (PF07714t)
RasGEF (PF00617)
RasGEF_N (PF00618)

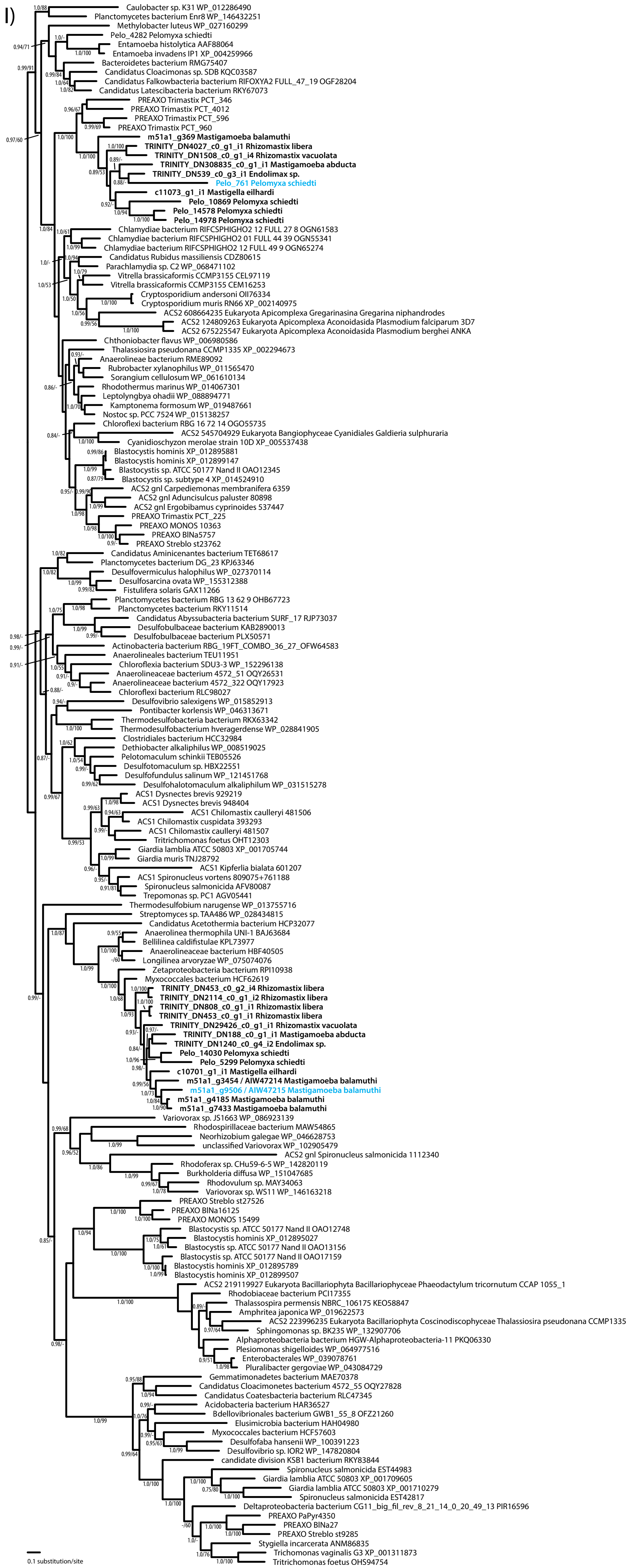
I)
Caulobacter sp. K31 WP_012286490
Planctomycetes bacterium Enr8 WP_146432251
Methylobacter luteus WP_027160299
Pelo_4282 Pelomyxa schiedti
Entamoeba histolytica AAF88064
Entamoeba invadens IP1 XP_004259966
Bacteroidetes bacterium RMG75407
Candidatus Cloacimonas sp. SDB KQC03587
Candidatus Falkowbacteria bacterium RIFOXYA2 FULL_47_19 OGF28204
Candidatus Latescibacteria bacterium RKY67073
PREAXO Trimastix PCT_346
PREAXO Trimastix PCT_4012
PREAXO Trimastix PCT_596
PREAXO Trimastix PCT_960
m51a1_g369 Mastigamoeba balamuthi
TRINITY_DN4027_c0_g1_i1 Rhizomastix libera
TRINITY_DN1508_c0_g1_i4 Rhizomastix vacuolata
TRINITY_DN308835_c0_g1_i1 Mastigamoeba abducta
TRINITY_DN539_c0_g3_i1 Endolimax sp.
Pelo_761 Pelomyxa schiedti
c11073_g1_i1 Mastigella eilhardi
Pelo_10869 Pelomyxa schiedti
Pelo_14578 Pelomyxa schiedti
Pelo_14978 Pelomyxa schiedti
Chlamydiae bacterium RIFCSPHIGHO2 12 FULL 27 8 OGN61583
Chlamydiae bacterium RIFCSPHIGHO2 01 FULL 44 39 OGN55341
Chlamydiae bacterium RIFCSPHIGHO2 12 FULL 49 9 OGN65274
Candidatus Rubidus massiliensis CDZ80615
Parachlamydia sp. C2 WP_068471102
Vitrella brassicaformis CCMP3155 CEL97119
Vitrella brassicaformis CCMP3155 CEM16253
Cryptosporidium andersoni OII76334
Cryptosporidium muris RN66 XP_002140975
ACS2 608664235 Eukaryota Apicomplexa Gregarinasina Gregarina niphandrodes
ACS2 124809263 Eukaryota Apicomplexa Aconoidasida Plasmodium falciparum 3D7
ACS2 675225547 Eukaryota Apicomplexa Aconoidasida Plasmodium berghei ANKA
Chthoniobacter flavus WP_006980586
Thalassiosira pseudonana CCMP1335 XP_002294673
Anaerolineae bacterium RME89092
Rubrobacter xylanophilus WP_011565470
Sorangium cellulosum WP_061610134
Rhodothermus marinus WP_014067301
Leptolyngbya ohadii WP_088894771
Kamptonema formosum WP_019487661
Nostoc sp. PCC 7524 WP_015138257
Chloroflexi bacterium RBG 16 72 14 OGO55735
ACS2 545704929 Eukaryota Bangiophyceae Cyanidiales Galdieria sulphuraria
Cyanidioschyzon merolae strain 10D XP_005537438
Blastocystis hominis XP_012895881
Blastocystis hominis XP_012899147
Blastocystis sp. ATCC 50177 Nand II OAO12345
Blastocystis sp. subtype 4 XP_014524910
ACS2 gnl Carpediemonas membranifera 6359
ACS2 gnl Aduncisulcus paluster 80898
ACS2 gnl Ergobibamus cyprinoides 537447
PREAXO Trimastix PCT_225
PREAXO MONOS 10363
PREAXO BINa5757
PREAXO Streblo st23762
Candidatus Aminicenantes bacterium TET68617
Planctomycetes bacterium DG_23 KPJ63346
Desulfovermiculus halophilus WP_027370114
Desulfosarcina ovata WP_155312388
Fistulifera solaris GAX11266
Planctomycetes bacterium RBG 13 62 9 OHB67723
Planctomycetes bacterium RKY11514
Candidatus Abyssubacteria bacterium SURF_17 RJP73037
Desulfobulbaceae bacterium KAB2890013
Desulfobulbaceae bacterium PLX50571
Actinobacteria bacterium RBG_19FT_COMBO_36_27_OFW64583
Anaerolineales bacterium TEU11951
Chloroflexia bacterium SDU3-3 WP_152296138
Anaerolineaceae bacterium 4572_51 OQY26531
Anaerolineaceae bacterium 4572_322 OQY17923
Chloroflexi bacterium RLC98027
Desulfovibrio salexigens WP_015852913
Pontibacter korlensis WP_046313671
Thermodesulfobacteria bacterium RKX63342
Thermodesulfobacterium hveragerdense WP_028841905
Clostridiales bacterium HCC32984
Dethiobacter alkaliphilus WP_008519025
Pelotomaculum schinkii TEB05526
Desulfotomaculum sp. HBX22551
Desulfofundulus salinum WP_121451768
Desulfohalotomaculum alkaliphilum WP_031515278
ACS1 Dysnectes brevis 929219
ACS1 Dysnectes brevis 948404
ACS1 Chilomastix caulleryi 481506
ACS1 Chilomastix cuspidata 393293
ACS1 Chilomastix caulleryi 481507
Tritrichomonas foetus OHT12303
Giardia lamblia ATCC 50803 XP_001705744
Giardia muris TNJ28792
ACS1 Kipferlia bialata 601207
ACS1 Spironucleus vortens 809075+761188
Spironucleus salmonicida AFV80087
Trepomonas sp. PC1 AGV05441
Thermodesulfobium narugense WP_013755716
Streptomyces sp. TAA486 WP_028434815
Candidatus Acetothermia bacterium HCP32077
Anaerolinea thermophila UNI-1 BAJ63684
Bellilinea caldifistulae KPL73977
Anaerolineaceae bacterium HBF40505
Longilinea arvoryzae WP_075074076
Zetaproteobacteria bacterium RPI10938
Myxococcales bacterium HCF62619
TRINITY_DN453_c0_g2_i4 Rhizomastix libera
TRINITY_DN2114_c0_g1_i2 Rhizomastix libera
TRINITY_DN808_c0_g1_i1 Rhizomastix libera
TRINITY_DN453_c0_g1_i1 Rhizomastix libera
TRINITY_DN29426_c0_g1_i1 Rhizomastix vacuolata
TRINITY_DN188_c0_g1_i1 Mastigamoeba abducta
TRINITY_DN1240_c0_g4_i2 Endolimax sp.
Pelo_14030 Pelomyxa schiedti
Pelo_5299 Pelomyxa schiedti
c10701_g1_i1 Mastigella eilhardi
m51a1_g3454 / AIW47214 Mastigamoeba balamuthi
m51a1_g9506 / AIW47215 Mastigamoeba balamuthi
m51a1_g4185 Mastigamoeba balamuthi
m51a1_g7433 Mastigamoeba balamuthi
Variovorax sp. JS1663 WP_086923139
Rhodospirillaceae bacterium MAW54865
Neorhizobium galegae WP_046628753
unclassified Variovorax WP_102905479
ACS2 gnl Spironucleus salmonicida 1112340
Rhodoferax sp. CHu59-6-5 WP_142820119
Burkholderia diffusa WP_151047685
Rhodovulum sp. MAY34063
Variovorax sp. WS11 WP_146163218
PREAXO Streblo st27526
PREAXO BINa16125
PREAXO MONOS 15499
Blastocystis sp. ATCC 50177 Nand II OAO12748
Blastocystis hominis XP_012895027
Blastocystis sp. ATCC 50177 Nand II OAO13156
Blastocystis sp. ATCC 50177 Nand II OAO17159
Blastocystis hominis XP_012895789
Blastocystis hominis XP_012899507
ACS2 219119927 Eukaryota Bacillariophyta Bacillariophyceae Phaeodactylum tricornutum CCAP 1055_1
Rhodobiaceae bacterium PCI17355
Thalassospira permensis NBRC_106175 KEO58847
Amphritea japonica WP_019622573
ACS2 223996235 Eukaryota Bacillariophyta Coscinodiscophyceae Thalassiosira pseudonana CCMP1335
Sphingomonas sp. BK235 WP_132907706
Alphaproteobacteria bacterium HGW-Alphaproteobacteria-11 PKQ06330
Plesiomonas shigelloides WP_064977516
Enterobacterales WP_039078761
Pluralibacter gergoviae WP_043084729
Gemmatimonadetes bacterium MAE70378
Candidatus Cloacimonetes bacterium 4572_55 OQY27828
Candidatus Coatesbacteria bacterium RLC47345
Acidobacteria bacterium HAR36527
Bdellovibrionales bacterium GWB1_55_8 OFZ21260
Elusimicrobia bacterium HAH04980
Myxococcales bacterium HCF57603
Desulfofaba hansenii WP_100391223
Desulfovibrio sp. IOR2 WP_147820804
candidate division KSB1 bacterium RKY83844
Spironucleus salmonicida EST44983
Giardia lamblia ATCC 50803 XP_001709605
Giardia lamblia ATCC 50803 XP_001710279
Spironucleus salmonicida EST42817
Deltaproteobacteria bacterium CG11_big_fil_rev_8_21_14_0_20_49_13 PIR16596
PREAXO PaPyr4350
PREAXO BINa27
PREAXO Streblo st9285
Stygiella incarcerata ANM86835
Trichomonas vaginalis G3 XP_001311873
Tritrichomonas foetus OHS94754
0.1 substitution/site

J)

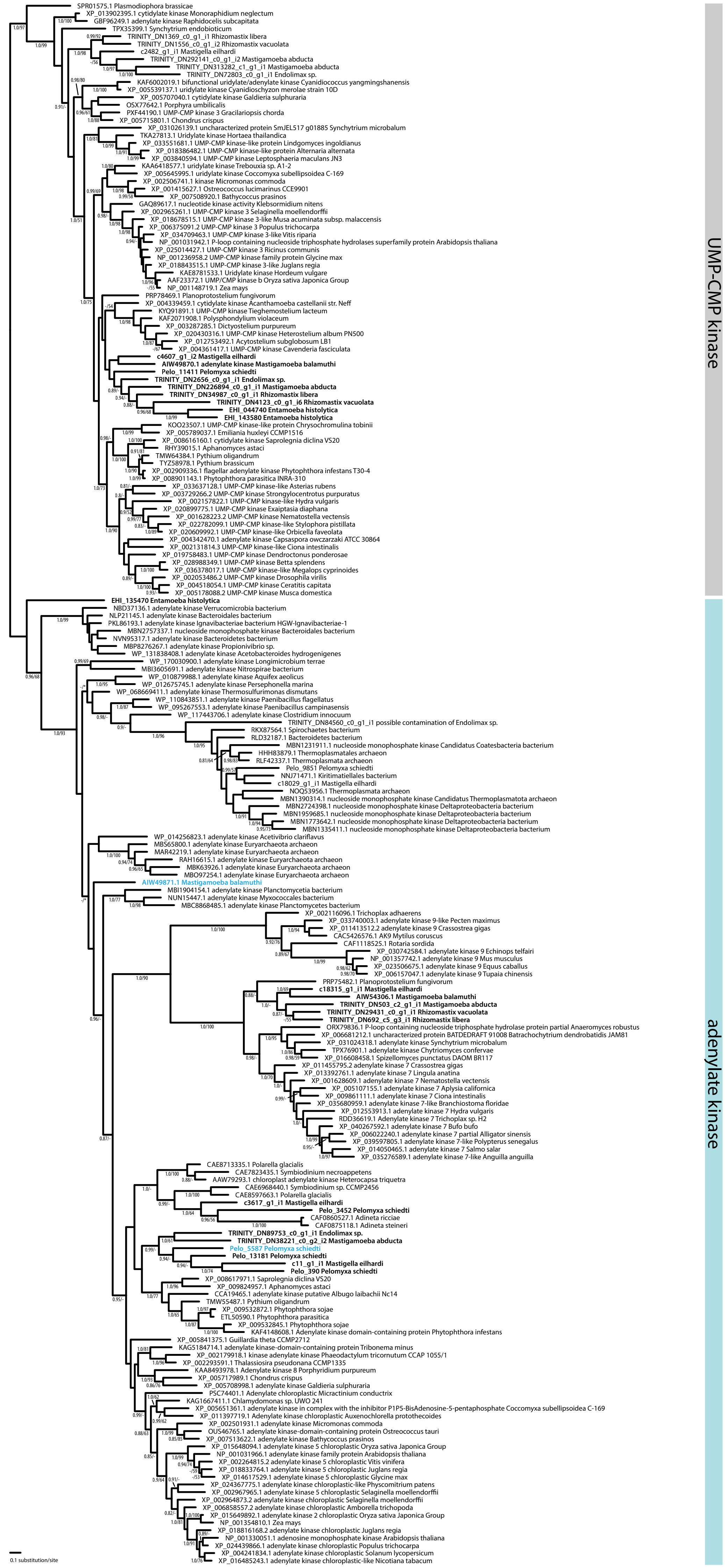

SPR01575.1 Plasmodiophora brassicae
XP_013902395.1 cytidylate kinase Monoraphidium neglectum
GBF96249.1 adenylate kinase Raphidocelis subcapitata
TPX35399.1 Synchytrium endobioticum
TRINITY_DN1369_c0_g1_i1 Rhizomastix libera
TRINITY_DN1556_c0_g1_i2 Rhizomastix vacuolata
c2482_g1_i1 Mastigella eilhardi
TRINITY_DN292141_c0_g1_i2 Mastigamoeba abducta
TRINITY_DN313282_c1_g1_i1 Mastigamoeba abducta
TRINITY_DN72803_c0_g1_i1 Endolimax sp.
KAF6002019.1 bifunctional uridylate/adenylate kinase Cyanidiococcus yangmingshanensis
XP_005539137.1 uridylate kinase Cyanidioschyzon merolae strain 10D
XP_005707040.1 cytidylate kinase Galdieria sulphuraria
OSX77642.1 Porphyra umbilicalis
PXF44190.1 UMP-CMP kinase 3 Gracilariopsis chorda
XP_005715801.1 Chondrus crispus
XP_031026139.1 uncharacterized protein SmJEL517 g01885 Synchytrium microbalum
TKA27813.1 Uridylate kinase Hortaea thailandica
XP_033551681.1 UMP-CMP kinase-like protein Lindgomyces ingoldianus
XP_018386482.1 UMP-CMP kinase-like protein Alternaria alternata
XP_003840594.1 UMP-CMP kinase Leptosphaeria maculans JN3
KAA6418577.1 uridylate kinase Trebouxia sp. A1-2
XP_005645995.1 uridylate kinase Coccomyxa subellipsoidea C-169
XP_002506741.1 kinase Micromonas commoda
XP_001415627.1 Ostreococcus lucimarinus CCE9901
XP_007508920.1 Bathycoccus prasinos
GAQ89617.1 nucleotide kinase activity Klebsormidium nitens
XP_002965261.1 UMP-CMP kinase 3 Selaginella moellendorffii
XP_018678515.1 UMP-CMP kinase 3-like Musa acuminata subsp. malaccensis
XP_006375091.2 UMP-CMP kinase 3 Populus trichocarpa
XP_034709463.1 UMP-CMP kinase 3-like Vitis riparia
NP_001031942.1 P-loop containing nucleoside triphosphate hydrolases superfamily protein Arabidopsis thaliana
XP_025014427.1 UMP-CMP kinase 3 Ricinus communis
NP_001236958.2 UMP-CMP kinase family protein Glycine max
XP_018843515.1 UMP-CMP kinase 3-like Juglans regia
KAE8781533.1 Uridylate kinase Hordeum vulgare
AAF23372.1 UMP/CMP kinase b Oryza sativa Japonica Group
NP_001148719.1 Zea mays
PRP78469.1 Planoprotostelium fungivorum
XP_004339459.1 cytidylate kinase Acanthamoeba castellanii str. Neff
KYQ91891.1 UMP-CMP kinase Tieghemostelium lacteum
KAF2071908.1 Polysphondylium violaceum
XP_003287285.1 Dictyostelium purpureum
XP_020430316.1 UMP-CMP kinase Heterostelium album PN500
XP_012753492.1 Acytostelium subglobosum LB1
XP_004361417.1 UMP-CMP kinase Cavenderia fasciculata
c4607_g1_i2 Mastigella eilhardi
AIW49870.1 adenylate kinase Mastigamoeba balamuthi
Pelo_11411 Pelomyxa schiedti
TRINITY_DN2656_c0_g1_i1 Endolimax sp.
TRINITY_DN226894_c0_g1_i1 Mastigamoeba abducta
TRINITY_DN34987_c0_g1_i1 Rhizomastix libera
TRINITY_DN4123_c0_g1_i6 Rhizomastix vacuolata
EHI_044740 Entamoeba histolytica
EHI_143580 Entamoeba histolytica
KOO23507.1 UMP-CMP kinase-like protein Chrysochromulina tobinii
XP_005789037.1 Emiliania huxleyi CCMP1516
XP_008616160.1 cytidylate kinase Saprolegnia diclina VS20
RHY39015.1 Aphanomyces astaci
TMW64384.1 Pythium oligandrum
TYZ58978.1 Pythium brassicum
XP_002909336.1 flagellar adenylate kinase Phytophthora infestans T30-4
XP_008901143.1 Phytophthora parasitica INRA-310
XP_033637128.1 UMP-CMP kinase-like Asterias rubens
XP_003729266.2 UMP-CMP kinase Strongylocentrotus purpuratus
XP_002157822.1 UMP-CMP kinase-like Hydra vulgaris
XP_020899775.1 UMP-CMP kinase Exaiptasia diaphana
XP_001628223.2 UMP-CMP kinase Nematostella vectensis
XP_022782099.1 UMP-CMP kinase-like Stylophora pistillata
XP_020609992.1 UMP-CMP kinase-like Orbicella faveolata
XP_004342470.1 adenylate kinase Capsaspora owczarzaki ATCC 30864
XP_002131814.3 UMP-CMP kinase-like Ciona intestinalis
XP_019758483.1 UMP-CMP kinase Dendroctonus ponderosae
XP_028988349.1 UMP-CMP kinase Betta splendens
XP_036378017.1 UMP-CMP kinase-like Megalops cyprinoides
XP_002053486.2 UMP-CMP kinase Drosophila virilis
XP_004518054.1 UMP-CMP kinase Ceratitis capitata
XP_005178088.2 UMP-CMP kinase Musca domestica
EHI_135470 Entamoeba histolytica
NBD37136.1 adenylate kinase Verrucomicrobia bacterium
NLP21145.1 adenylate kinase Bacteroidales bacterium
PKL86193.1 adenylate kinase Ignavibacteriae bacterium HGW-Ignavibacteriae-1
MBN2757337.1 nucleoside monophosphate kinase Bacteroidales bacterium
NVN95317.1 adenylate kinase Bacteroidetes bacterium
MBP8276267.1 adenylate kinase Propionivibrio sp.
WP_131838408.1 adenylate kinase Acetobacteroides hydrogenigenes
WP_170030900.1 adenylate kinase Longimicrobium terrae
MBI3605691.1 adenylate kinase Nitrospirae bacterium
WP_010879988.1 adenylate kinase Aquifex aeolicus
WP_012675745.1 adenylate kinase Persephonella marina
WP_068669411.1 adenylate kinase Thermosulfurimonas dismutans
WP_110843851.1 adenylate kinase Paenibacillus flagellatus
WP_095267553.1 adenylate kinase Paenibacillus campinasensis
WP_117443706.1 adenylate kinase Clostridium innocuum
TRINITY_DN84560_c0_g1_i1 possible contamination of Endolimax sp.
RKX87564.1 Spirochaetes bacterium
RLD32187.1 Bacteroidetes bacterium
MBN1231911.1 nucleoside monophosphate kinase Candidatus Coatesbacteria bacterium
HHH83879.1 Thermoplasmatales archaeon
RLF42337.1 Thermoplasmata archaeon
Pelo_9851 Pelomyxa schiedti
NNJ71471.1 Kiritimatiellales bacterium
c18029_g1_i1 Mastigella eilhardi
NOQ53956.1 Thermoplasmata archaeon
MBN1390314.1 nucleoside monophosphate kinase Candidatus Thermoplasmatota archaeon
MBN2724398.1 nucleoside monophosphate kinase Deltaproteobacteria bacterium
MBN1959685.1 nucleoside monophosphate kinase Deltaproteobacteria bacterium
MBN1773642.1 nucleoside monophosphate kinase Deltaproteobacteria bacterium
MBN1335411.1 nucleoside monophosphate kinase Deltaproteobacteria bacterium
WP_014256823.1 adenylate kinase Acetivibrio clariflavus
MBS65800.1 adenylate kinase Euryarchaeota archaeon
MAR42219.1 adenylate kinase Euryarchaeota archaeon
RAH16615.1 adenylate kinase Euryarchaeota archaeon
MBK63926.1 adenylate kinase Euryarchaeota archaeon
MBO97254.1 adenylate kinase Euryarchaeota archaeon
AIW49871.1 Mastigamoeba balamuthi
MBI1904154.1 adenylate kinase Planctomycetia bacterium
NUN15447.1 adenylate kinase Myxococcales bacterium
MBC8868485.1 adenylate kinase Planctomycetes bacterium
XP_002116096.1 Trichoplax adhaerens
XP_033740003.1 adenylate kinase 9-like Pecten maximus
XP_011413512.2 adenylate kinase 9 Crassostrea gigas
CAC5426576.1 AK9 Mytilus coruscus
CAF1118525.1 Rotaria sordida
XP_030742584.1 adenylate kinase 9 Echinops telfairi
NP_001357742.1 adenylate kinase 9 Mus musculus
XP_023506675.1 adenylate kinase 9 Equus caballus
XP_006157047.1 adenylate kinase 9 Tupaia chinensis
PRP75482.1 Planoprotostelium fungivorum
c18315_g1_i1 Mastigella eilhardi
AIW54306.1 Mastigamoeba balamuthi
TRINITY_DN503_c2_g1_i1 Mastigamoeba abducta
TRINITY_DN29431_c0_g1_i1 Rhizomastix vacuolata
TRINITY_DN692_c5_g3_i1 Rhizomastix libera
ORX79836.1 P-loop containing nucleoside triphosphate hydrolase protein partial Anaeromyces robustus
XP_006681212.1 uncharacterized protein BATDEDRAFT 91008 Batrachochytrium dendrobatidis JAM81
XP_031024318.1 adenylate kinase Synchytrium microbalum
TPX76901.1 adenylate kinase Chytriomyces confervae
XP_016608458.1 Spizellomyces punctatus DAOM BR117
XP_011455795.2 adenylate kinase 7 Crassostrea gigas
XP_013392761.1 adenylate kinase 7 Lingula anatina
XP_001628609.1 adenylate kinase 7 Nematostella vectensis
XP_005107155.1 adenylate kinase 7 Aplysia californica
XP_009861111.1 adenylate kinase 7 Ciona intestinalis
XP_035680959.1 adenylate kinase 7-like Branchiostoma floridae
XP_012553913.1 adenylate kinase 7 Hydra vulgaris
RDD36619.1 Adenylate kinase 7 Trichoplax sp. H2
XP_040267592.1 adenylate kinase 7 Bufo bufo
XP_006022240.1 adenylate kinase 7 partial Alligator sinensis
XP_039597805.1 adenylate kinase 7-like Polypterus senegalus
XP_014050465.1 adenylate kinase 7 Salmo salar
XP_035276589.1 adenylate kinase 7-like Anguilla anguilla
CAE8713335.1 Polarella glacialis
CAE7823435.1 Symbiodinium necroappetens
AAW79293.1 chloroplast adenylate kinase Heterocapsa triquetra
CAE6968440.1 Symbiodinium sp. CCMP2456
CAE8597663.1 Polarella glacialis
c3617_g1_i1 Mastigella eilhardi
Pelo_3452 Pelomyxa schiedti
CAF0860527.1 Adineta ricciae
CAF0875118.1 Adineta steineri
TRINITY_DN89753_c0_g1_i1 Endolimax sp.
TRINITY_DN38221_c0_g2_i2 Mastigamoeba abducta
Pelo_5587 Pelomyxa schiedti
Pelo_13181 Pelomyxa schiedti
c11_g1_i1 Mastigella eilhardi
Pelo_390 Pelomyxa schiedti
XP_008617971.1 Saprolegnia diclina VS20
XP_009824957.1 Aphanomyces astaci
CCA19465.1 adenylate kinase putative Albugo laibachii Nc14
TMW55487.1 Pythium oligandrum
XP_009532872.1 Phytophthora sojae
ETL50590.1 Phytophthora parasitica
XP_009532845.1 Phytophthora sojae
KAF4148608.1 Adenylate kinase domain-containing protein Phytophthora infestans
XP_005841375.1 Guillardia theta CCMP2712
KAG5184714.1 adenylate kinase-domain-containing protein Tribonema minus
XP_002179918.1 kinase adenylate kinase Phaeodactylum tricornutum CCAP 1055/1
XP_002293591.1 Thalassiosira pseudonana CCMP1335
KAA8493978.1 Adenylate kinase 8 Porphyridium purpureum
XP_005717989.1 Chondrus crispus
XP_005708998.1 adenylate kinase Galdieria sulphuraria
PSC74401.1 Adenylate chloroplastic Micractinium conductrix
KAG1667411.1 Chlamydomonas sp. UWO 241
XP_005651361.1 adenylate kinase in complex with the inhibitor P1P5-BisAdenosine-5-pentaphosphate Coccomyxa subellipsoidea C-169
XP_011397719.1 Adenylate kinase chloroplastic Auxenochlorella protothecoides
XP_002501931.1 adenylate kinase Micromonas commoda
OUS46765.1 adenylate kinase-domain-containing protein Ostreococcus tauri
XP_007513622.1 adenylate kinase Bathycoccus prasinos
XP_015648094.1 adenylate kinase 5 chloroplastic Oryza sativa Japonica Group
NP_001031966.1 adenylate kinase family protein Arabidopsis thaliana
XP_002264815.2 adenylate kinase 5 chloroplastic Vitis vinifera
XP_018833764.1 adenylate kinase 5 chloroplastic Juglans regia
XP_014617529.1 adenylate kinase 5 chloroplastic Glycine max
XP_024367775.1 adenylate kinase chloroplastic-like Physcomitrium patens
XP_002967965.1 adenylate kinase 5 chloroplastic Selaginella moellendorffii
XP_002964873.2 adenylate kinase chloroplastic Selaginella moellendorffii
XP_006858557.2 adenylate kinase chloroplastic Amborella trichopoda
XP_015649892.1 adenylate kinase 2 chloroplastic Oryza sativa Japonica Group
NP_001354810.1 Zea mays
XP_018816168.2 adenylate kinase chloroplastic Juglans regia
NP_001330051.1 adenosine monophosphate kinase Arabidopsis thaliana
XP_024439866.1 adenylate kinase chloroplastic Populus trichocarpa
XP_004241834.1 adenylate kinase chloroplastic Solanum lycopersicum
XP_016485243.1 adenylate kinase chloroplastic-like Nicotiana tabacum
UMP-CMP kinase
adenylate kinase
0.1 substitution/site

K)

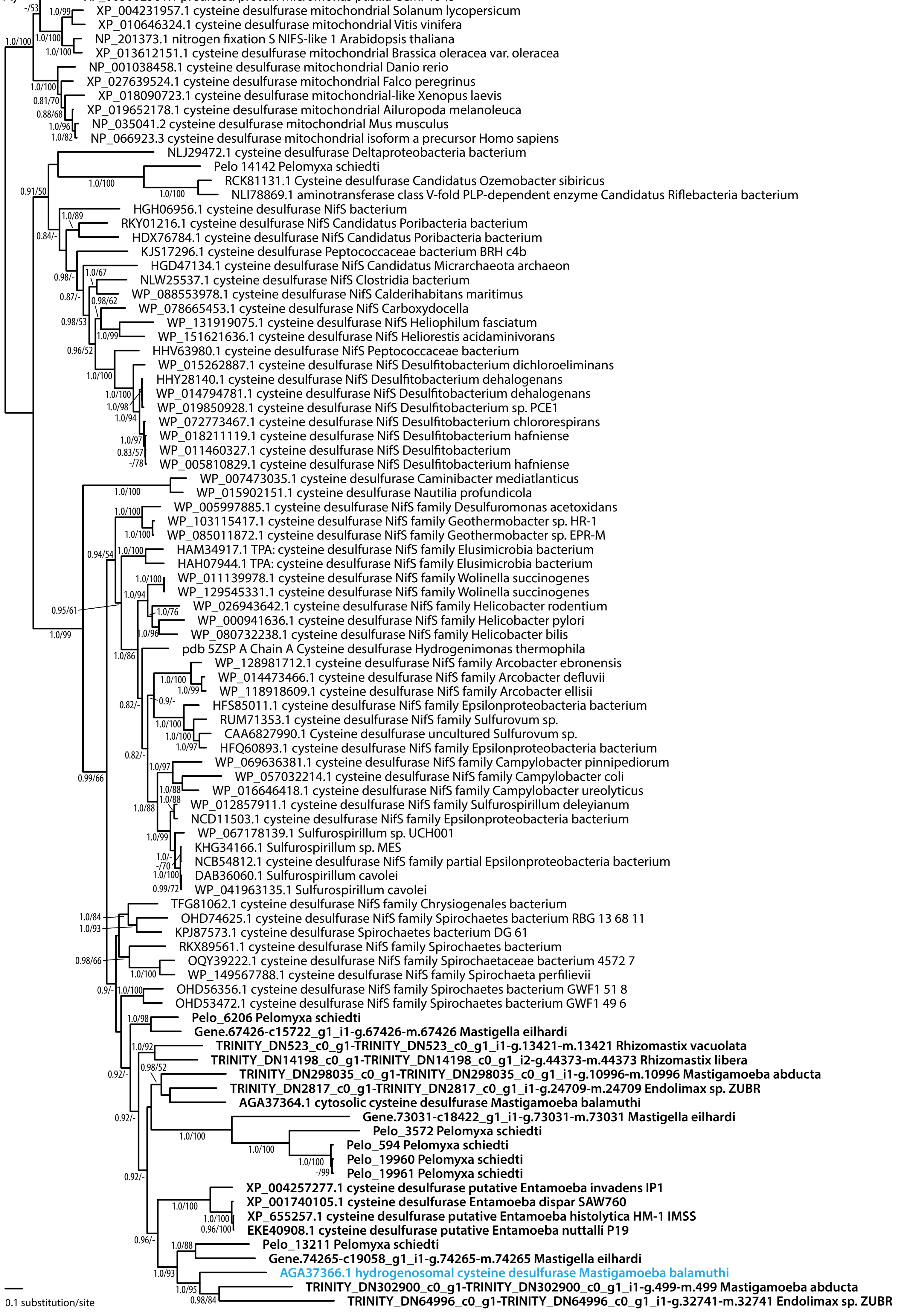

XP_003062581.1 predicted protein Micromonas pusilla CCMP1545
XP_004231957.1 cysteine desulfurase mitochondrial Solanum lycopersicum
XP_010646324.1 cysteine desulfurase mitochondrial Vitis vinifera
NP_201373.1 nitrogen fixation S NIFS-like 1 Arabidopsis thaliana
XP_013612151.1 cysteine desulfurase mitochondrial Brassica oleracea var. oleracea
NP_001038458.1 cysteine desulfurase mitochondrial Danio rerio
XP_027639524.1 cysteine desulfurase mitochondrial Falco peregrinus
XP_018090723.1 cysteine desulfurase mitochondrial-like Xenopus laevis
XP_019652178.1 cysteine desulfurase mitochondrial Ailuropoda melanoleuca
NP_035041.2 cysteine desulfurase mitochondrial Mus musculus
NP_066923.3 cysteine desulfurase mitochondrial isoform a precursor Homo sapiens
NLJ29472.1 cysteine desulfurase Deltaproteobacteria bacterium
Pelo 14142 Pelomyxa schiedti
RCK81131.1 Cysteine desulfurase Candidatus Ozemobacter sibiricus
NLI78869.1 aminotransferase class V-fold PLP-dependent enzyme Candidatus Riflebacteria bacterium
HGH06956.1 cysteine desulfurase NifS bacterium
RKY01216.1 cysteine desulfurase NifS Candidatus Poribacteria bacterium
HDX76784.1 cysteine desulfurase NifS Candidatus Poribacteria bacterium
KJS17296.1 cysteine desulfurase Peptococcaceae bacterium BRH c4b
HGD47134.1 cysteine desulfurase NifS Candidatus Micrarchaeota archaeon
NLW25537.1 cysteine desulfurase NifS Clostridia bacterium
WP_088553978.1 cysteine desulfurase NifS Calderihabitans maritimus
WP_078665453.1 cysteine desulfurase NifS Carboxydocella
WP_131919075.1 cysteine desulfurase NifS Heliophilum fasciatum
WP_151621636.1 cysteine desulfurase NifS Heliorestis acidaminivorans
HHV63980.1 cysteine desulfurase NifS Peptococcaceae bacterium
WP_015262887.1 cysteine desulfurase NifS Desulfitobacterium dichloroeliminans
HHY28140.1 cysteine desulfurase NifS Desulfitobacterium dehalogenans
WP_014794781.1 cysteine desulfurase NifS Desulfitobacterium dehalogenans
WP_019850928.1 cysteine desulfurase NifS Desulfitobacterium sp. PCE1
WP_072773467.1 cysteine desulfurase NifS Desulfitobacterium chlororespirans
WP_018211119.1 cysteine desulfurase NifS Desulfitobacterium hafniense
WP_011460327.1 cysteine desulfurase NifS Desulfitobacterium
WP_005810829.1 cysteine desulfurase NifS Desulfitobacterium hafniense
WP_007473035.1 cysteine desulfurase Caminibacter mediatlanticus
WP_015902151.1 cysteine desulfurase Nautilia profundicola
WP_005997885.1 cysteine desulfurase NifS family Desulfuromonas acetoxidans
WP_103115417.1 cysteine desulfurase NifS family Geothermobacter sp. HR-1
WP_085011872.1 cysteine desulfurase NifS family Geothermobacter sp. EPR-M
HAM34917.1 TPA: cysteine desulfurase NifS family Elusimicrobia bacterium
HAH07944.1 TPA: cysteine desulfurase NifS family Elusimicrobia bacterium
WP_011139978.1 cysteine desulfurase NifS family Wolinella succinogenes
WP_129545331.1 cysteine desulfurase NifS family Wolinella succinogenes
WP_026943642.1 cysteine desulfurase NifS family Helicobacter rodentium
WP_000941636.1 cysteine desulfurase NifS family Helicobacter pylori
WP_080732238.1 cysteine desulfurase NifS family Helicobacter bilis
pdb 5ZSP A Chain A Cysteine desulfurase Hydrogenimonas thermophila
WP_128981712.1 cysteine desulfurase NifS family Arcobacter ebronensis
WP_014473466.1 cysteine desulfurase NifS family Arcobacter defluvii
WP_118918609.1 cysteine desulfurase NifS family Arcobacter ellisii
HFS85011.1 cysteine desulfurase NifS family Epsilonproteobacteria bacterium
RUM71353.1 cysteine desulfurase NifS family Sulfurovum sp.
CAA6827990.1 Cysteine desulfurase uncultured Sulfurovum sp.
HFQ60893.1 cysteine desulfurase NifS family Epsilonproteobacteria bacterium
WP_069636381.1 cysteine desulfurase NifS family Campylobacter pinnipediorum
WP_057032214.1 cysteine desulfurase NifS family Campylobacter coli
WP_016646418.1 cysteine desulfurase NifS family Campylobacter ureolyticus
WP_012857911.1 cysteine desulfurase NifS family Sulfurospirillum deleyianum
NCD11503.1 cysteine desulfurase NifS family Epsilonproteobacteria bacterium
WP_067178139.1 Sulfurospirillum sp. UCH001
KHG34166.1 Sulfurospirillum sp. MES
NCB54812.1 cysteine desulfurase NifS family partial Epsilonproteobacteria bacterium
DAB36060.1 Sulfurospirillum cavolei
WP_041963135.1 Sulfurospirillum cavolei
TFG81062.1 cysteine desulfurase NifS family Chrysiogenales bacterium
OHD74625.1 cysteine desulfurase NifS family Spirochaetes bacterium RBG 13 68 11
KPJ87573.1 cysteine desulfurase Spirochaetes bacterium DG 61
RKX89561.1 cysteine desulfurase NifS family Spirochaetes bacterium
OQY39222.1 cysteine desulfurase NifS family Spirochaetaceae bacterium 4572 7
WP_149567788.1 cysteine desulfurase NifS family Spirochaeta perfilievii
OHD56356.1 cysteine desulfurase NifS family Spirochaetes bacterium GWF1 51 8
OHD53472.1 cysteine desulfurase NifS family Spirochaetes bacterium GWF1 49 6
Pelo_6206 Pelomyxa schiedti
Gene.67426-c15722_g1_i1-g.67426-m.67426 Mastigella eilhardi
TRINITY_DN523_c0_g1-TRINITY_DN523_c0_g1_i1-g.13421-m.13421 Rhizomastix vacuolata
TRINITY_DN14198_c0_g1-TRINITY_DN14198_c0_g1_i2-g.44373-m.44373 Rhizomastix libera
TRINITY_DN298035_c0_g1-TRINITY_DN298035_c0_g1_i1-g.10996-m.10996 Mastigamoeba abducta
TRINITY_DN2817_c0_g1-TRINITY_DN2817_c0_g1_i1-g.24709-m.24709 Endolimax sp. ZUBR
AGA37364.1 cytosolic cysteine desulfurase Mastigamoeba balamuthi
Gene.73031-c18422_g1_i1-g.73031-m.73031 Mastigella eilhardi
Pelo_3572 Pelomyxa schiedti
Pelo_594 Pelomyxa schiedti
Pelo_19960 Pelomyxa schiedti
Pelo_19961 Pelomyxa schiedti
XP_004257277.1 cysteine desulfurase putative Entamoeba invadens IP1
XP_001740105.1 cysteine desulfurase Entamoeba dispar SAW760
XP_655257.1 cysteine desulfurase putative Entamoeba histolytica HM-1 IMSS
EKE40908.1 cysteine desulfurase putative Entamoeba nuttalli P19
Pelo_13211 Pelomyxa schiedti
Gene.74265-c19058_g1_i1-g.74265-m.74265 Mastigella eilhardi
AGA37366.1 hydrogenosomal cysteine desulfurase Mastigamoeba balamuthi
TRINITY_DN302900_c0_g1-TRINITY_DN302900_c0_g1_i1-g.499-m.499 Mastigamoeba abducta
TRINITY_DN64996_c0_g1-TRINITY_DN64996_c0_g1_i1-g.32741-m.32741 Endolimax sp. ZUBR
0.1 substitution/site

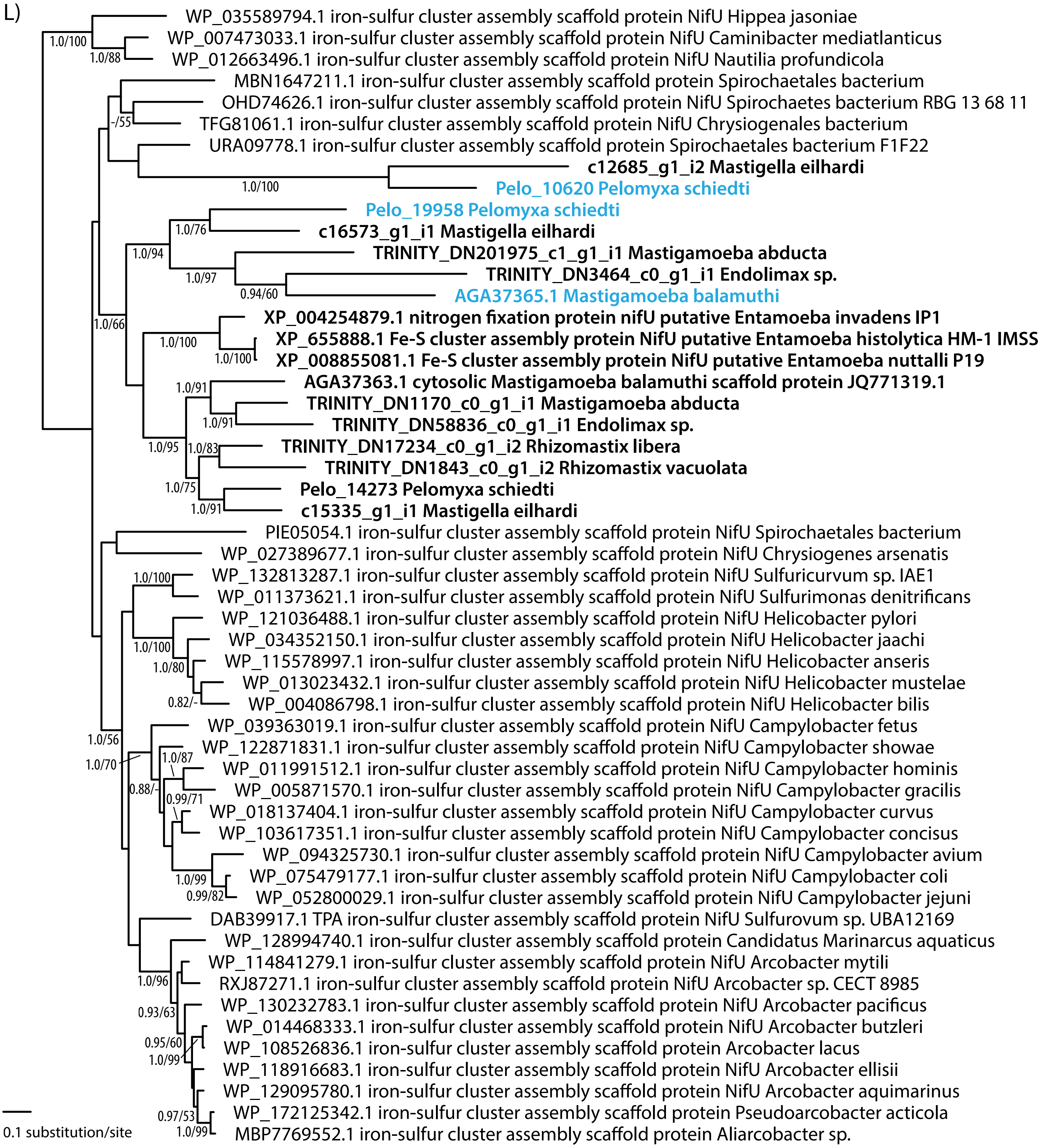

L)
WP_035589794.1 iron-sulfur cluster assembly scaffold protein NifU Hippea jasoniae
WP_007473033.1 iron-sulfur cluster assembly scaffold protein NifU Caminibacter mediatlanticus
WP_012663496.1 iron-sulfur cluster assembly scaffold protein NifU Nautilia profundicola
MBN1647211.1 iron-sulfur cluster assembly scaffold protein Spirochaetales bacterium
OHD74626.1 iron-sulfur cluster assembly scaffold protein NifU Spirochaetes bacterium RBG 13 68 11
TFG81061.1 iron-sulfur cluster assembly scaffold protein NifU Chrysiogenales bacterium
URA09778.1 iron-sulfur cluster assembly scaffold protein Spirochaetales bacterium F1F22
c12685_g1_i2 Mastigella eilhardi
Pelo_10620 Pelomyxa schiedti
Pelo_19958 Pelomyxa schiedti
c16573_g1_i1 Mastigella eilhardi
TRINITY_DN201975_c1_g1_i1 Mastigamoeba abducta
TRINITY_DN3464_c0_g1_i1 Endolimax sp.
AGA37365.1 Mastigamoeba balamuthi
XP_004254879.1 nitrogen fixation protein nifU putative Entamoeba invadens IP1
XP_655888.1 Fe-S cluster assembly protein NifU putative Entamoeba histolytica HM-1 IMSS
XP_008855081.1 Fe-S cluster assembly protein NifU putative Entamoeba nuttalli P19
AGA37363.1 cytosolic Mastigamoeba balamuthi scaffold protein JQ771319.1
TRINITY_DN1170_c0_g1_i1 Mastigamoeba abducta
TRINITY_DN58836_c0_g1_i1 Endolimax sp.
TRINITY_DN17234_c0_g1_i2 Rhizomastix libera
TRINITY_DN1843_c0_g1_i2 Rhizomastix vacuolata
Pelo_14273 Pelomyxa schiedti
c15335_g1_i1 Mastigella eilhardi
PIE05054.1 iron-sulfur cluster assembly scaffold protein NifU Spirochaetales bacterium
WP_027389677.1 iron-sulfur cluster assembly scaffold protein NifU Chrysiogenes arsenatis
WP_132813287.1 iron-sulfur cluster assembly scaffold protein NifU Sulfuricurvum sp. IAE1
WP_011373621.1 iron-sulfur cluster assembly scaffold protein NifU Sulfurimonas denitrificans
WP_121036488.1 iron-sulfur cluster assembly scaffold protein NifU Helicobacter pylori
WP_034352150.1 iron-sulfur cluster assembly scaffold protein NifU Helicobacter jaachi
WP_115578997.1 iron-sulfur cluster assembly scaffold protein NifU Helicobacter anseris
WP_013023432.1 iron-sulfur cluster assembly scaffold protein NifU Helicobacter mustelae
WP_004086798.1 iron-sulfur cluster assembly scaffold protein NifU Helicobacter bilis
WP_039363019.1 iron-sulfur cluster assembly scaffold protein NifU Campylobacter fetus
WP_122871831.1 iron-sulfur cluster assembly scaffold protein NifU Campylobacter showae
WP_011991512.1 iron-sulfur cluster assembly scaffold protein NifU Campylobacter hominis
WP_005871570.1 iron-sulfur cluster assembly scaffold protein NifU Campylobacter gracilis
WP_018137404.1 iron-sulfur cluster assembly scaffold protein NifU Campylobacter curvus
WP_103617351.1 iron-sulfur cluster assembly scaffold protein NifU Campylobacter concisus
WP_094325730.1 iron-sulfur cluster assembly scaffold protein NifU Campylobacter avium
WP_075479177.1 iron-sulfur cluster assembly scaffold protein NifU Campylobacter coli
WP_052800029.1 iron-sulfur cluster assembly scaffold protein NifU Campylobacter jejuni
DAB39917.1 TPA iron-sulfur cluster assembly scaffold protein NifU Sulfurovum sp. UBA12169
WP_128994740.1 iron-sulfur cluster assembly scaffold protein Candidatus Marinarcus aquaticus
WP_114841279.1 iron-sulfur cluster assembly scaffold protein NifU Arcobacter mytili
RXJ87271.1 iron-sulfur cluster assembly scaffold protein NifU Arcobacter sp. CECT 8985
WP_130232783.1 iron-sulfur cluster assembly scaffold protein NifU Arcobacter pacificus
WP_014468333.1 iron-sulfur cluster assembly scaffold protein NifU Arcobacter butzleri
WP_108526836.1 iron-sulfur cluster assembly scaffold protein Arcobacter lacus
WP_118916683.1 iron-sulfur cluster assembly scaffold protein NifU Arcobacter ellisii
WP_129095780.1 iron-sulfur cluster assembly scaffold protein NifU Arcobacter aquimarinus
WP_172125342.1 iron-sulfur cluster assembly scaffold protein Pseudoarcobacter acticola
MBP7769552.1 iron-sulfur cluster assembly scaffold protein Aliarcobacter sp.
0.1 substitution/site

M)

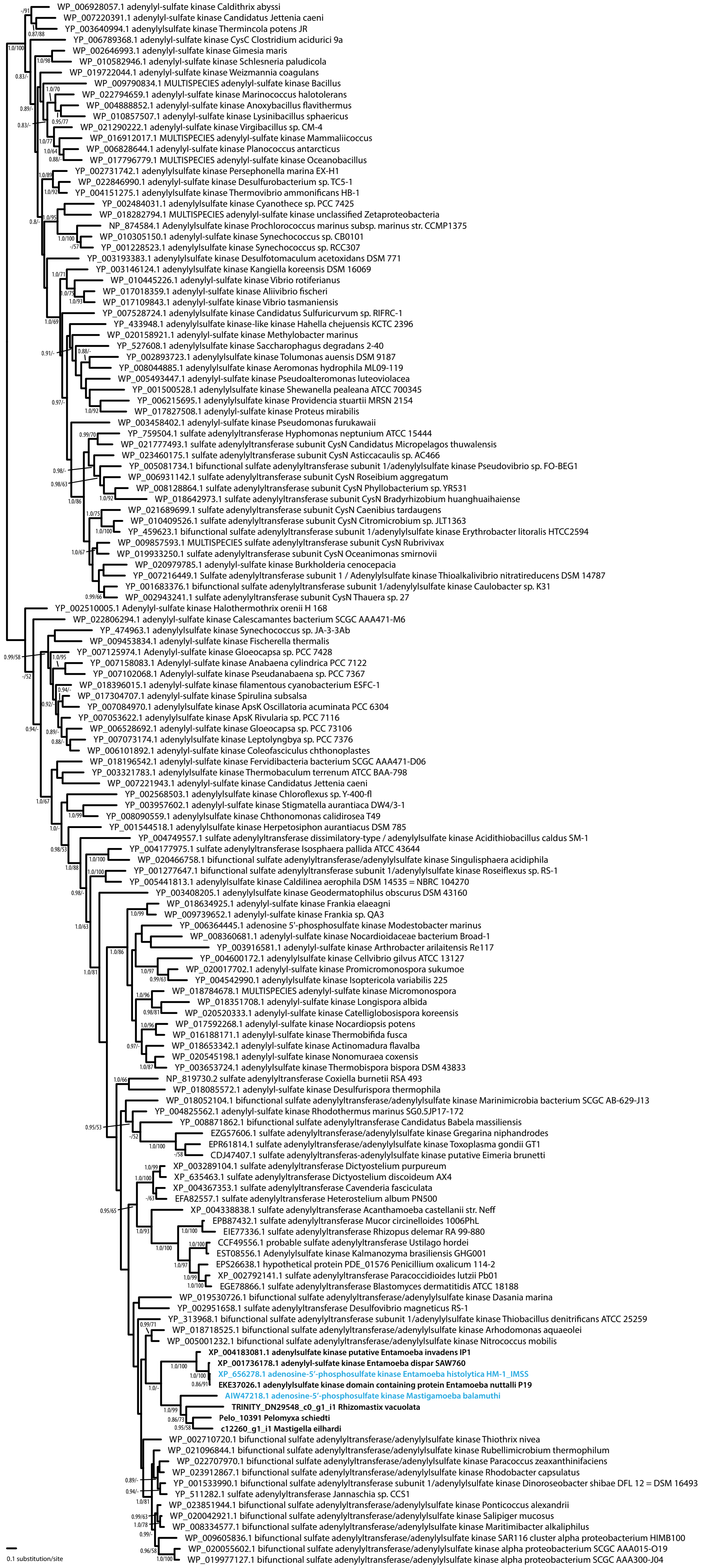
WP_006928057.1 adenylyl-sulfate kinase Caldithrix abyssi
WP_007220391.1 adenylyl-sulfate kinase Candidatus Jettenia caeni
YP_003640994.1 adenylylsulfate kinase Thermincola potens JR
YP_006789368.1 adenylyl-sulfate kinase CysC Clostridium acidurici 9a
WP_002646993.1 adenylyl-sulfate kinase Gimesia maris
WP_010582946.1 adenylyl-sulfate kinase Schlesneria paludicola
WP_019722044.1 adenylyl-sulfate kinase Weizmannia coagulans
WP_009790834.1 MULTISPECIES adenylyl-sulfate kinase Bacillus
WP_022794659.1 adenylyl-sulfate kinase Marinococcus halotolerans
WP_004888852.1 adenylyl-sulfate kinase Anoxybacillus flavithermus
WP_010857507.1 adenylyl-sulfate kinase Lysinibacillus sphaericus
WP_021290222.1 adenylyl-sulfate kinase Virgibacillus sp. CM-4
WP_016912017.1 MULTISPECIES adenylyl-sulfate kinase Mammaliicoccus
WP_006828644.1 adenylyl-sulfate kinase Planococcus antarcticus
WP_017796779.1 MULTISPECIES adenylyl-sulfate kinase Oceanobacillus
YP_002731742.1 adenylylsulfate kinase Persephonella marina EX-H1
WP_022846990.1 adenylyl-sulfate kinase Desulfurobacterium sp. TC5-1
YP_004151275.1 adenylylsulfate kinase Thermovibrio ammonificans HB-1
YP_002484031.1 adenylylsulfate kinase Cyanothece sp. PCC 7425
WP_018282794.1 MULTISPECIES adenylyl-sulfate kinase unclassified Zetaproteobacteria
NP_874584.1 Adenylylsulfate kinase Prochlorococcus marinus subsp. marinus str. CCMP1375
WP_010305150.1 adenylyl-sulfate kinase Synechococcus sp. CB0101
YP_001228523.1 adenylylsulfate kinase Synechococcus sp. RCC307
YP_003193383.1 adenylylsulfate kinase Desulfotomaculum acetoxidans DSM 771
YP_003146124.1 adenylylsulfate kinase Kangiella koreensis DSM 16069
WP_010445226.1 adenylyl-sulfate kinase Vibrio rotiferianus
WP_017018359.1 adenylyl-sulfate kinase Aliivibrio fischeri
WP_017109843.1 adenylyl-sulfate kinase Vibrio tasmaniensis
YP_007528724.1 adenylylsulfate kinase Candidatus Sulfuricurvum sp. RIFRC-1
YP_433948.1 adenylylsulfate kinase-like kinase Hahella chejuensis KCTC 2396
WP_020158921.1 adenylyl-sulfate kinase Methylobacter marinus
YP_527608.1 adenylylsulfate kinase Saccharophagus degradans 2-40
YP_002893723.1 adenylylsulfate kinase Tolumonas auensis DSM 9187
YP_008044885.1 adenylylsulfate kinase Aeromonas hydrophila ML09-119
WP_005493447.1 adenylyl-sulfate kinase Pseudoalteromonas luteoviolacea
YP_001500528.1 adenylylsulfate kinase Shewanella pealeana ATCC 700345
YP_006215695.1 adenylylsulfate kinase Providencia stuartii MRSN 2154
WP_017827508.1 adenylyl-sulfate kinase Proteus mirabilis
WP_003458402.1 adenylyl-sulfate kinase Pseudomonas furukawaii
YP_759504.1 sulfate adenylyltransferase Hyphomonas neptunium ATCC 15444
WP_021777493.1 sulfate adenylyltransferase subunit CysN Candidatus Micropelagos thuwalensis
WP_023460175.1 sulfate adenylyltransferase subunit CysN Asticcacaulis sp. AC466
YP_005081734.1 bifunctional sulfate adenylyltransferase subunit 1/adenylylsulfate kinase Pseudovibrio sp. FO-BEG1
WP_006931142.1 sulfate adenylyltransferase subunit CysN Roseibium aggregatum
WP_008128864.1 sulfate adenylyltransferase subunit CysN Phyllobacterium sp. YR531
WP_018642973.1 sulfate adenylyltransferase subunit CysN Bradyrhizobium huanghuaihaiense
WP_021689699.1 sulfate adenylyltransferase subunit CysN Caenibius tardaugens
WP_010409526.1 sulfate adenylyltransferase subunit CysN Citromicrobium sp. JLT1363
YP_459623.1 bifunctional sulfate adenylyltransferase subunit 1/adenylylsulfate kinase Erythrobacter litoralis HTCC2594
WP_009857593.1 MULTISPECIES sulfate adenylyltransferase subunit CysN Rubrivivax
WP_019933250.1 sulfate adenylyltransferase subunit CysN Oceanimonas smirnovii
WP_020979785.1 adenylyl-sulfate kinase Burkholderia cenocepacia
YP_007216449.1 Sulfate adenylyltransferase subunit 1 / Adenylylsulfate kinase Thioalkalivibrio nitratireducens DSM 14787
YP_001683376.1 bifunctional sulfate adenylyltransferase subunit 1/adenylylsulfate kinase Caulobacter sp. K31
WP_002943241.1 sulfate adenylyltransferase subunit CysN Thauera sp. 27
YP_002510005.1 Adenylyl-sulfate kinase Halothermothrix orenii H 168
WP_022806294.1 adenylyl-sulfate kinase Calescamantes bacterium SCGC AAA471-M6
YP_474963.1 adenylylsulfate kinase Synechococcus sp. JA-3-3Ab
WP_009453834.1 adenylyl-sulfate kinase Fischerella thermalis
YP_007125974.1 Adenylyl-sulfate kinase Gloeocapsa sp. PCC 7428
YP_007158083.1 Adenylyl-sulfate kinase Anabaena cylindrica PCC 7122
YP_007102068.1 Adenylyl-sulfate kinase Pseudanabaena sp. PCC 7367
WP_018396015.1 adenylyl-sulfate kinase filamentous cyanobacterium ESFC-1
WP_017304707.1 adenylyl-sulfate kinase Spirulina subsalsa
YP_007084970.1 adenylylsulfate kinase ApsK Oscillatoria acuminata PCC 6304
YP_007053622.1 adenylylsulfate kinase ApsK Rivularia sp. PCC 7116
WP_006528692.1 adenylyl-sulfate kinase Gloeocapsa sp. PCC 73106
YP_007073174.1 adenylylsulfate kinase Leptolyngbya sp. PCC 7376
WP_006101892.1 adenylyl-sulfate kinase Coleofasciculus chthonoplastes
WP_018196542.1 adenylyl-sulfate kinase Fervidibacteria bacterium SCGC AAA471-D06
YP_003321783.1 adenylylsulfate kinase Thermobaculum terrenum ATCC BAA-798
WP_007221943.1 adenylyl-sulfate kinase Candidatus Jettenia caeni
YP_002568503.1 adenylylsulfate kinase Chloroflexus sp. Y-400-fl
YP_003957602.1 adenylyl-sulfate kinase Stigmatella aurantiaca DW4/3-1
YP_008090559.1 adenylylsulfate kinase Chthonomonas calidirosea T49
YP_001544518.1 adenylylsulfate kinase Herpetosiphon aurantiacus DSM 785
YP_004749557.1 sulfate adenylyltransferase dissimilatory-type / adenylylsulfate kinase Acidithiobacillus caldus SM-1
YP_004177975.1 sulfate adenylyltransferase Isosphaera pallida ATCC 43644
WP_020466758.1 bifunctional sulfate adenylyltransferase/adenylylsulfate kinase Singulisphaera acidiphila
YP_001277647.1 bifunctional sulfate adenylyltransferase subunit 1/adenylylsulfate kinase Roseiflexus sp. RS-1
YP_005441813.1 adenylylsulfate kinase Caldilinea aerophila DSM 14535 = NBRC 104270
YP_003408205.1 adenylylsulfate kinase Geodermatophilus obscurus DSM 43160
WP_018634925.1 adenylyl-sulfate kinase Frankia elaeagni
WP_009739652.1 adenylyl-sulfate kinase Frankia sp. QA3
YP_006364445.1 adenosine 5'-phosphosulfate kinase Modestobacter marinus
WP_008360681.1 adenylyl-sulfate kinase Nocardioidaceae bacterium Broad-1
YP_003916581.1 adenylyl-sulfate kinase Arthrobacter arilaitensis Re117
YP_004600172.1 adenylylsulfate kinase Cellvibrio gilvus ATCC 13127
WP_020017702.1 adenylyl-sulfate kinase Promicromonospora sukumoe
YP_004542990.1 adenylylsulfate kinase Isoptericola variabilis 225
WP_018784678.1 MULTISPECIES adenylyl-sulfate kinase Micromonospora
WP_018351708.1 adenylyl-sulfate kinase Longispora albida
WP_020520333.1 adenylyl-sulfate kinase Catelliglobosispora koreensis
WP_017592268.1 adenylyl-sulfate kinase Nocardiopsis potens
WP_016188171.1 adenylyl-sulfate kinase Thermobifida fusca
WP_018653342.1 adenylyl-sulfate kinase Actinomadura flavalba
WP_020545198.1 adenylyl-sulfate kinase Nonomuraea coxensis
YP_003653724.1 adenylylsulfate kinase Thermobispora bispora DSM 43833
NP_819730.2 sulfate adenylyltransferase Coxiella burnetii RSA 493
WP_018085572.1 adenylyl-sulfate kinase Desulfurispora thermophila
WP_018052104.1 bifunctional sulfate adenylyltransferase/adenylylsulfate kinase Marinimicrobia bacterium SCGC AB-629-J13
YP_004825562.1 adenylyl-sulfate kinase Rhodothermus marinus SG0.5JP17-172
YP_008871862.1 bifunctional sulfate adenylyltransferase Candidatus Babela massiliensis
EZG57606.1 sulfate adenylyltransferase/adenylylsulfate kinase Gregarina niphandrodes
EPR61814.1 sulfate adenylyltransferase/adenylylsulfate kinase Toxoplasma gondii GT1
CDJ47407.1 sulfate adenylyltransferas-adenylylsulfate kinase putative Eimeria brunetti
XP_003289104.1 sulfate adenylyltransferase Dictyostelium purpureum
XP_635463.1 sulfate adenylyltransferase Dictyostelium discoideum AX4
XP_004367353.1 sulfate adenylyltransferase Cavenderia fasciculata
EFA82557.1 sulfate adenylyltransferase Heterostelium album PN500
XP_004338838.1 sulfate adenylyltransferase Acanthamoeba castellanii str. Neff
EPB87432.1 sulfate adenylyltransferase Mucor circinelloides 1006PhL
EIE77336.1 sulfate adenylyltransferase Rhizopus delemar RA 99-880
CCF49556.1 probable sulfate adenylyltransferase Ustilago hordei
EST08556.1 Adenylylsulfate kinase Kalmanozyma brasiliensis GHG001
EPS26638.1 hypothetical protein PDE_01576 Penicillium oxalicum 114-2
XP_002792141.1 sulfate adenylyltransferase Paracoccidioides lutzii Pb01
EGE78866.1 sulfate adenylyltransferase Blastomyces dermatitidis ATCC 18188
WP_019530726.1 bifunctional sulfate adenylyltransferase/adenylylsulfate kinase Dasania marina
YP_002951658.1 sulfate adenylyltransferase Desulfovibrio magneticus RS-1
YP_313968.1 bifunctional sulfate adenylyltransferase subunit 1/adenylylsulfate kinase Thiobacillus denitrificans ATCC 25259
WP_018718525.1 bifunctional sulfate adenylyltransferase/adenylylsulfate kinase Arhodomonas aquaeolei
WP_005001232.1 bifunctional sulfate adenylyltransferase/adenylylsulfate kinase Nitrococcus mobilis
XP_004183081.1 adenylsulfate kinase putative Entamoeba invadens IP1
XP_001736178.1 adenylyl-sulfate kinase Entamoeba dispar SAW760
XP_656278.1 adenosine-5'-phosphosulfate kinase Entamoeba histolytica HM-1_IMSS
EKE37026.1 adenylylsulfate kinase domain containing protein Entamoeba nuttalli P19
AIW47218.1 adenosine-5'-phosphosulfate kinase Mastigamoeba balamuthi
TRINITY_DN29548_c0_g1_i1 Rhizomastix vacuolata
Pelo_10391 Pelomyxa schiedti
c12260_g1_i1 Mastigella eilhardi
WP_002710720.1 bifunctional sulfate adenylyltransferase/adenylylsulfate kinase Thiothrix nivea
WP_021096844.1 bifunctional sulfate adenylyltransferase/adenylylsulfate kinase Rubellimicrobium thermophilum
WP_022707970.1 bifunctional sulfate adenylyltransferase/adenylylsulfate kinase Paracoccus zeaxanthinifaciens
WP_023912867.1 bifunctional sulfate adenylyltransferase/adenylylsulfate kinase Rhodobacter capsulatus
YP_001533990.1 bifunctional sulfate adenylyltransferase subunit 1/adenylylsulfate kinase Dinoroseobacter shibae DFL 12 = DSM 16493
YP_511282.1 sulfate adenylyltransferase Jannaschia sp. CCS1
WP_023851944.1 bifunctional sulfate adenylyltransferase/adenylylsulfate kinase Ponticoccus alexandrii
WP_020042921.1 bifunctional sulfate adenylyltransferase/adenylylsulfate kinase Salipiger mucosus
WP_008334577.1 bifunctional sulfate adenylyltransferase/adenylylsulfate kinase Maritimibacter alkaliphilus
WP_009605836.1 bifunctional sulfate adenylyltransferase/adenylylsulfate kinase SAR116 cluster alpha proteobacterium HIMB100
WP_020055602.1 bifunctional sulfate adenylyltransferase/adenylylsulfate kinase alpha proteobacterium SCGC AAA015-O19
WP_019977127.1 bifunctional sulfate adenylyltransferase/adenylylsulfate kinase alpha proteobacterium SCGC AAA300-J04
0.1 substitution/site

N)

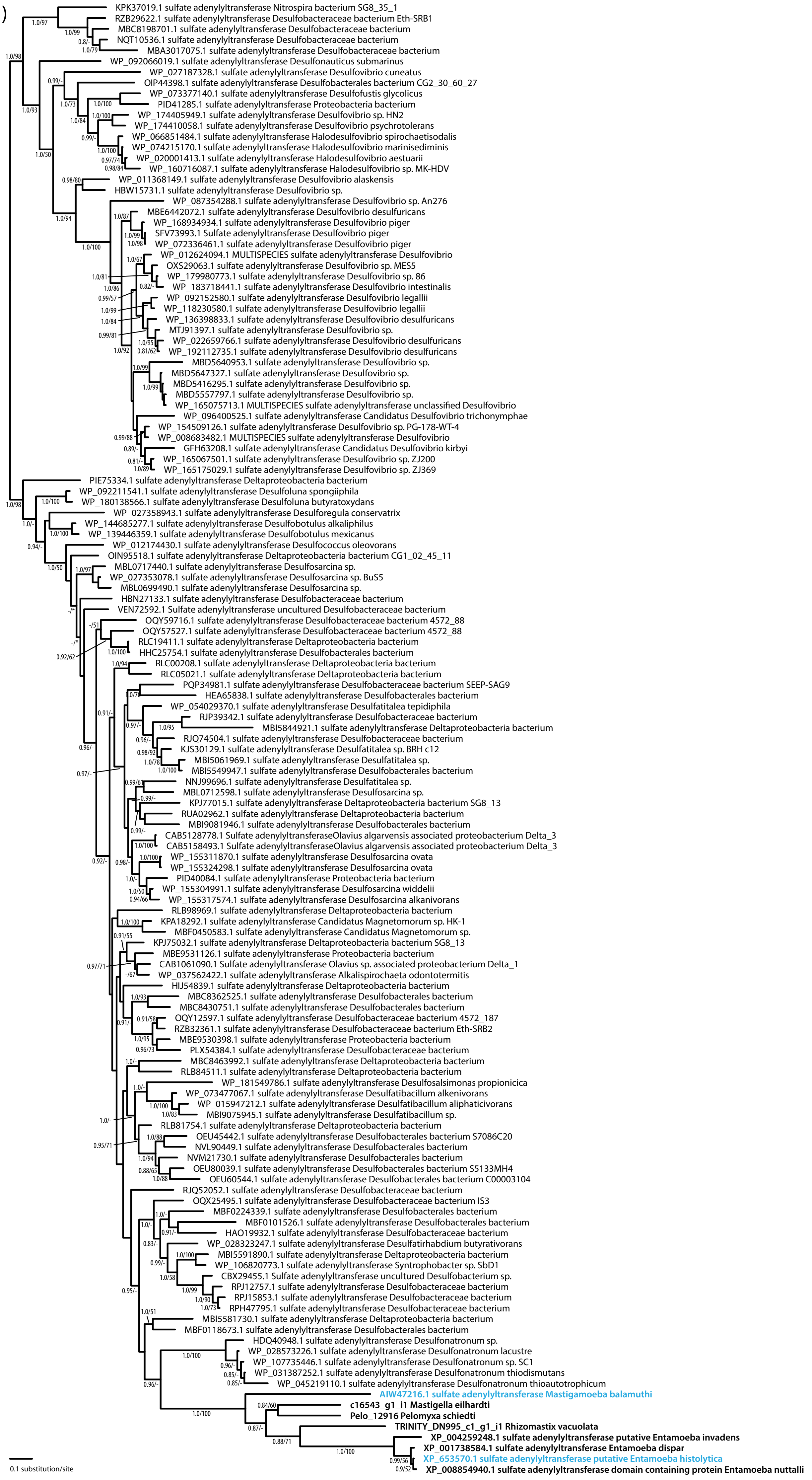
KPK37019.1 sulfate adenylyltransferase Nitrospira bacterium SG8_35_1
RZB29622.1 sulfate adenylyltransferase Desulfobacteraceae bacterium Eth-SRB1
MBC8198701.1 sulfate adenylyltransferase Desulfobacteraceae bacterium
NQT10536.1 sulfate adenylyltransferase Desulfobacteraceae bacterium
MBA3017075.1 sulfate adenylyltransferase Desulfobacteraceae bacterium
WP_092066019.1 sulfate adenylyltransferase Desulfonauticus submarinus
WP_027187328.1 sulfate adenylyltransferase Desulfovibrio cuneatus
OIP44398.1 sulfate adenylyltransferase Desulfobacterales bacterium CG2_30_60_27
WP_073377140.1 sulfate adenylyltransferase Desulfofustis glycolicus
PID41285.1 sulfate adenylyltransferase Proteobacteria bacterium
WP_174405949.1 sulfate adenylyltransferase Desulfovibrio sp. HN2
WP_174410058.1 sulfate adenylyltransferase Desulfovibrio psychrotolerans
WP_066851484.1 sulfate adenylyltransferase Halodesulfovibrio spirochaetisodalis
WP_074215170.1 sulfate adenylyltransferase Halodesulfovibrio marinisediminis
WP_020001413.1 sulfate adenylyltransferase Halodesulfovibrio aestuarii
WP_160716087.1 sulfate adenylyltransferase Halodesulfovibrio sp. MK-HDV
WP_011368149.1 sulfate adenylyltransferase Desulfovibrio alaskensis
HBW15731.1 sulfate adenylyltransferase Desulfovibrio sp.
WP_087354288.1 sulfate adenylyltransferase Desulfovibrio sp. An276
MBE6442072.1 sulfate adenylyltransferase Desulfovibrio desulfuricans
WP_168934934.1 sulfate adenylyltransferase Desulfovibrio piger
SFV73993.1 Sulfate adenylyltransferase Desulfovibrio piger
WP_072336461.1 sulfate adenylyltransferase Desulfovibrio piger
WP_012624094.1 MULTISPECIES sulfate adenylyltransferase Desulfovibrio
OXS29063.1 sulfate adenylyltransferase Desulfovibrio sp. MES5
WP_179980773.1 sulfate adenylyltransferase Desulfovibrio sp. 86
WP_183718441.1 sulfate adenylyltransferase Desulfovibrio intestinalis
WP_092152580.1 sulfate adenylyltransferase Desulfovibrio legallii
WP_118230580.1 sulfate adenylyltransferase Desulfovibrio legallii
WP_136398833.1 sulfate adenylyltransferase Desulfovibrio desulfuricans
MTJ91397.1 sulfate adenylyltransferase Desulfovibrio sp.
WP_022659766.1 sulfate adenylyltransferase Desulfovibrio desulfuricans
WP_192112735.1 sulfate adenylyltransferase Desulfovibrio desulfuricans
MBD5640953.1 sulfate adenylyltransferase Desulfovibrio sp.
MBD5647327.1 sulfate adenylyltransferase Desulfovibrio sp.
MBD5416295.1 sulfate adenylyltransferase Desulfovibrio sp.
MBD5557797.1 sulfate adenylyltransferase Desulfovibrio sp.
WP_165075713.1 MULTISPECIES sulfate adenylyltransferase unclassified Desulfovibrio
WP_096400525.1 sulfate adenylyltransferase Candidatus Desulfovibrio trichonymphae
WP_154509126.1 sulfate adenylyltransferase Desulfovibrio sp. PG-178-WT-4
WP_008683482.1 MULTISPECIES sulfate adenylyltransferase Desulfovibrio
GFH63208.1 sulfate adenylyltransferase Candidatus Desulfovibrio kirbyi
WP_165067501.1 sulfate adenylyltransferase Desulfovibrio sp. ZJ200
WP_165175029.1 sulfate adenylyltransferase Desulfovibrio sp. ZJ369
PIE75334.1 sulfate adenylyltransferase Deltaproteobacteria bacterium
WP_092211541.1 sulfate adenylyltransferase Desulfoluna spongiiphila
WP_180138566.1 sulfate adenylyltransferase Desulfoluna butyratoxydans
WP_027358943.1 sulfate adenylyltransferase Desulforegula conservatrix
WP_144685277.1 sulfate adenylyltransferase Desulfobotulus alkaliphilus
WP_139446359.1 sulfate adenylyltransferase Desulfobotulus mexicanus
WP_012174430.1 sulfate adenylyltransferase Desulfococcus oleovorans
OIN95518.1 sulfate adenylyltransferase Deltaproteobacteria bacterium CG1_02_45_11
MBL0717440.1 sulfate adenylyltransferase Desulfosarcina sp.
WP_027353078.1 sulfate adenylyltransferase Desulfosarcina sp. BuS5
MBL0699490.1 sulfate adenylyltransferase Desulfosarcina sp.
HBN27133.1 sulfate adenylyltransferase Desulfobacteraceae bacterium
VEN72592.1 Sulfate adenylyltransferase uncultured Desulfobacteraceae bacterium
OQY59716.1 sulfate adenylyltransferase Desulfobacteraceae bacterium 4572_88
OQY57527.1 sulfate adenylyltransferase Desulfobacteraceae bacterium 4572_88
RLC19411.1 sulfate adenylyltransferase Deltaproteobacteria bacterium
HHC25754.1 sulfate adenylyltransferase Desulfobacterales bacterium
RLC00208.1 sulfate adenylyltransferase Deltaproteobacteria bacterium
RLC05021.1 sulfate adenylyltransferase Deltaproteobacteria bacterium
PQP34981.1 sulfate adenylyltransferase Desulfobacteraceae bacterium SEEP-SAG9
HEA65838.1 sulfate adenylyltransferase Desulfobacterales bacterium
WP_054029370.1 sulfate adenylyltransferase Desulfatitalea tepidiphila
RJP39342.1 sulfate adenylyltransferase Desulfobacteraceae bacterium
MBI5844921.1 sulfate adenylyltransferase Deltaproteobacteria bacterium
RJQ74504.1 sulfate adenylyltransferase Desulfobacteraceae bacterium
KJS30129.1 sulfate adenylyltransferase Desulfatitalea sp. BRH c12
MBI5061969.1 sulfate adenylyltransferase Desulfatitalea sp.
MBI5549947.1 sulfate adenylyltransferase Desulfobacterales bacterium
NNJ99696.1 sulfate adenylyltransferase Desulfatitalea sp.
MBL0712598.1 sulfate adenylyltransferase Desulfosarcina sp.
KPJ77015.1 sulfate adenylyltransferase Deltaproteobacteria bacterium SG8_13
RUA02962.1 sulfate adenylyltransferase Deltaproteobacteria bacterium
MBI9081946.1 sulfate adenylyltransferase Desulfobacterales bacterium
CAB5128778.1 Sulfate adenylyltransferaseOlavius algarvensis associated proteobacterium Delta_3
CAB5158493.1 Sulfate adenylyltransferaseOlavius algarvensis associated proteobacterium Delta_3
WP_155311870.1 sulfate adenylyltransferase Desulfosarcina ovata
WP_155324298.1 sulfate adenylyltransferase Desulfosarcina ovata
PID40084.1 sulfate adenylyltransferase Proteobacteria bacterium
WP_155304991.1 sulfate adenylyltransferase Desulfosarcina widdelii
WP_155317574.1 sulfate adenylyltransferase Desulfosarcina alkanivorans
RLB98969.1 sulfate adenylyltransferase Deltaproteobacteria bacterium
KPA18292.1 sulfate adenylyltransferase Candidatus Magnetomorum sp. HK-1
MBF0450583.1 sulfate adenylyltransferase Candidatus Magnetomorum sp.
KPJ75032.1 sulfate adenylyltransferase Deltaproteobacteria bacterium SG8_13
MBE9531126.1 sulfate adenylyltransferase Proteobacteria bacterium
CAB1061090.1 Sulfate adenylyltransferase Olavius sp. associated proteobacterium Delta_1
WP_037562422.1 sulfate adenylyltransferase Alkalispirochaeta odontotermitis
HIJ54839.1 sulfate adenylyltransferase Deltaproteobacteria bacterium
MBC8362525.1 sulfate adenylyltransferase Desulfobacterales bacterium
MBC8430751.1 sulfate adenylyltransferase Desulfobacterales bacterium
OQY12597.1 sulfate adenylyltransferase Desulfobacteraceae bacterium 4572_187
RZB32361.1 sulfate adenylyltransferase Desulfobacteraceae bacterium Eth-SRB2
MBE9530398.1 sulfate adenylyltransferase Proteobacteria bacterium
PLX54384.1 sulfate adenylyltransferase Desulfobacteraceae bacterium
MBC8463992.1 sulfate adenylyltransferase Deltaproteobacteria bacterium
RLB84511.1 sulfate adenylyltransferase Deltaproteobacteria bacterium
WP_181549786.1 sulfate adenylyltransferase Desulfosalsimonas propionicica
WP_073477067.1 sulfate adenylyltransferase Desulfatibacillum alkenivorans
WP_015947212.1 sulfate adenylyltransferase Desulfatibacillum aliphaticivorans
MBI9075945.1 sulfate adenylyltransferase Desulfatibacillum sp.
RLB81754.1 sulfate adenylyltransferase Deltaproteobacteria bacterium
OEU45442.1 sulfate adenylyltransferase Desulfobacterales bacterium S7086C20
NVL90449.1 sulfate adenylyltransferase Desulfobacterales bacterium
NVM21730.1 sulfate adenylyltransferase Desulfobacterales bacterium
OEU80039.1 sulfate adenylyltransferase Desulfobacterales bacterium S5133MH4
OEU60544.1 sulfate adenylyltransferase Desulfobacterales bacterium C00003104
RJQ52052.1 sulfate adenylyltransferase Desulfobacteraceae bacterium
OQX25495.1 sulfate adenylyltransferase Desulfobacteraceae bacterium IS3
MBF0224339.1 sulfate adenylyltransferase Desulfobacterales bacterium
MBF0101526.1 sulfate adenylyltransferase Desulfobacterales bacterium
HAO19932.1 sulfate adenylyltransferase Desulfobacteraceae bacterium
WP_028323247.1 sulfate adenylyltransferase Desulfatirhabdium butyrativorans
MBI5591890.1 sulfate adenylyltransferase Deltaproteobacteria bacterium
WP_106820773.1 sulfate adenylyltransferase Syntrophobacter sp. SbD1
CBX29455.1 Sulfate adenylyltransferase uncultured Desulfobacterium sp.
RPJ12757.1 sulfate adenylyltransferase Desulfobacteraceae bacterium
RPJ15853.1 sulfate adenylyltransferase Desulfobacteraceae bacterium
RPH47795.1 sulfate adenylyltransferase Desulfobacteraceae bacterium
MBI5581730.1 sulfate adenylyltransferase Deltaproteobacteria bacterium
MBF0118673.1 sulfate adenylyltransferase Desulfobacterales bacterium
HDQ40948.1 sulfate adenylyltransferase Desulfonatronum sp.
WP_028573226.1 sulfate adenylyltransferase Desulfonatronum lacustre
WP_107735446.1 sulfate adenylyltransferase Desulfonatronum sp. SC1
WP_031387252.1 sulfate adenylyltransferase Desulfonatronum thiodismutans
WP_045219110.1 sulfate adenylyltransferase Desulfonatronum thioautotrophicum
AIW47216.1 sulfate adenylyltransferase Mastigamoeba balamuthi
c16543_g1_i1 Mastigella eilhardti
Pelo_12916 Pelomyxa schiedti
TRINITY_DN995_c1_g1_i1 Rhizomastix vacuolata
XP_004259248.1 sulfate adenylyltransferase putative Entamoeba invadens
XP_001738584.1 sulfate adenylyltransferase Entamoeba dispar
XP_653570.1 sulfate adenylyltransferase putative Entamoeba histolytica
XP_008854940.1 sulfate adenylyltransferase domain containing protein Entamoeba nuttalli
0.1 substitution/site

O)

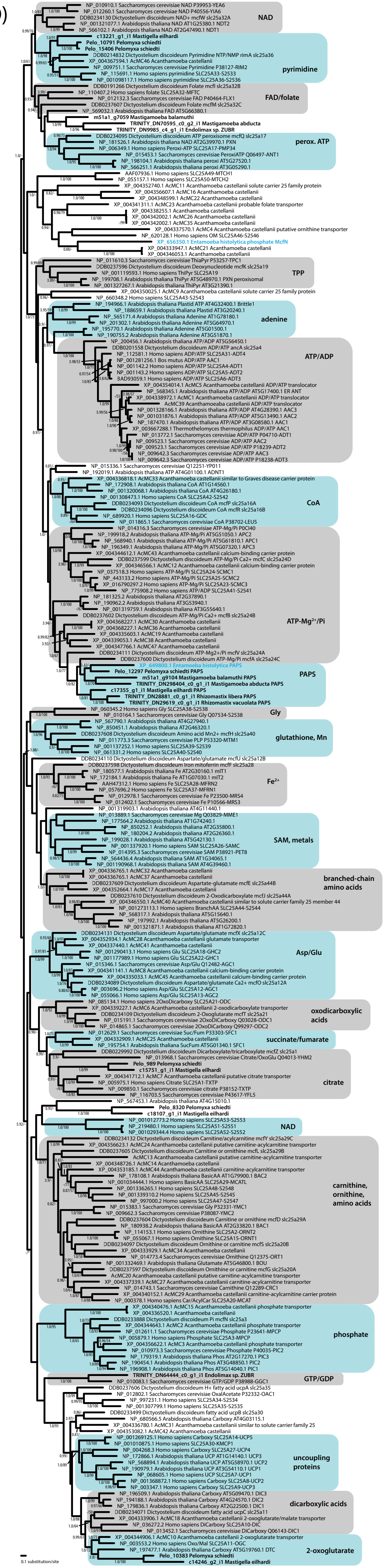

Fig. S4. Phylogenetic analyses of selected proteins. A) AAT, B) GCS-P, C) SHMT, D) LplA, E) GCS-L, F) PFO, G) LDH, H) HydA, I) ACS, J) AK, K) NifS, L) NifU, M) APSK, N) AS, O) MCF transporters. The topology of phylogenetic tree is shown from Bayesian analysis. Support values are shown as posterior probabilities (PP) from MrBayes / bootstrap supports (BS) from RAxML. Support values for PP < 0.8 and BS < 50% are denoted by a dash (-), whereas an asterisk (*) marks a topology that was not retrieved in a particular analysis. Archamoebal sequences are in bold. Proteins previously shown as MRO-targeted are highlighted by blue. For HydA (**H**), domain composition is shown as explained in the graphical legend. Note that MrBayes runs did not converge for GCS-L (**E**) even after 50,000,000 generations (standard deviation of split frequencies was >0.10).

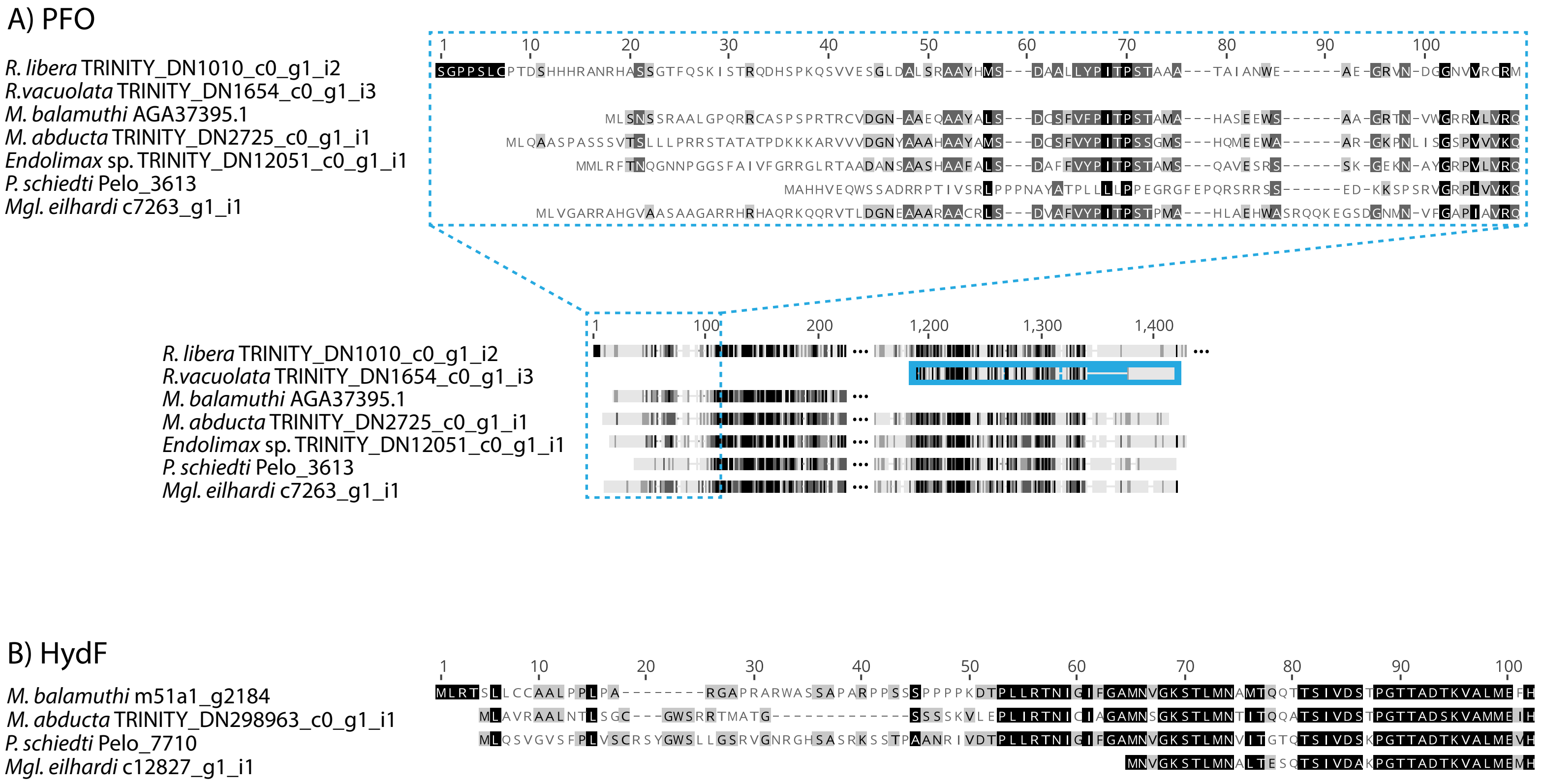

Fig. S5. Alignments of N-terminal parts of (A) PFO and (B) HydF. In (A), full alignment is shown below the zoomed part to illustrate the missing N-terminal portion of PFO sequence identified in *R. vacuolata* (shown by blue background). Gray and black boxes show sequence similarities and identities, respectively.

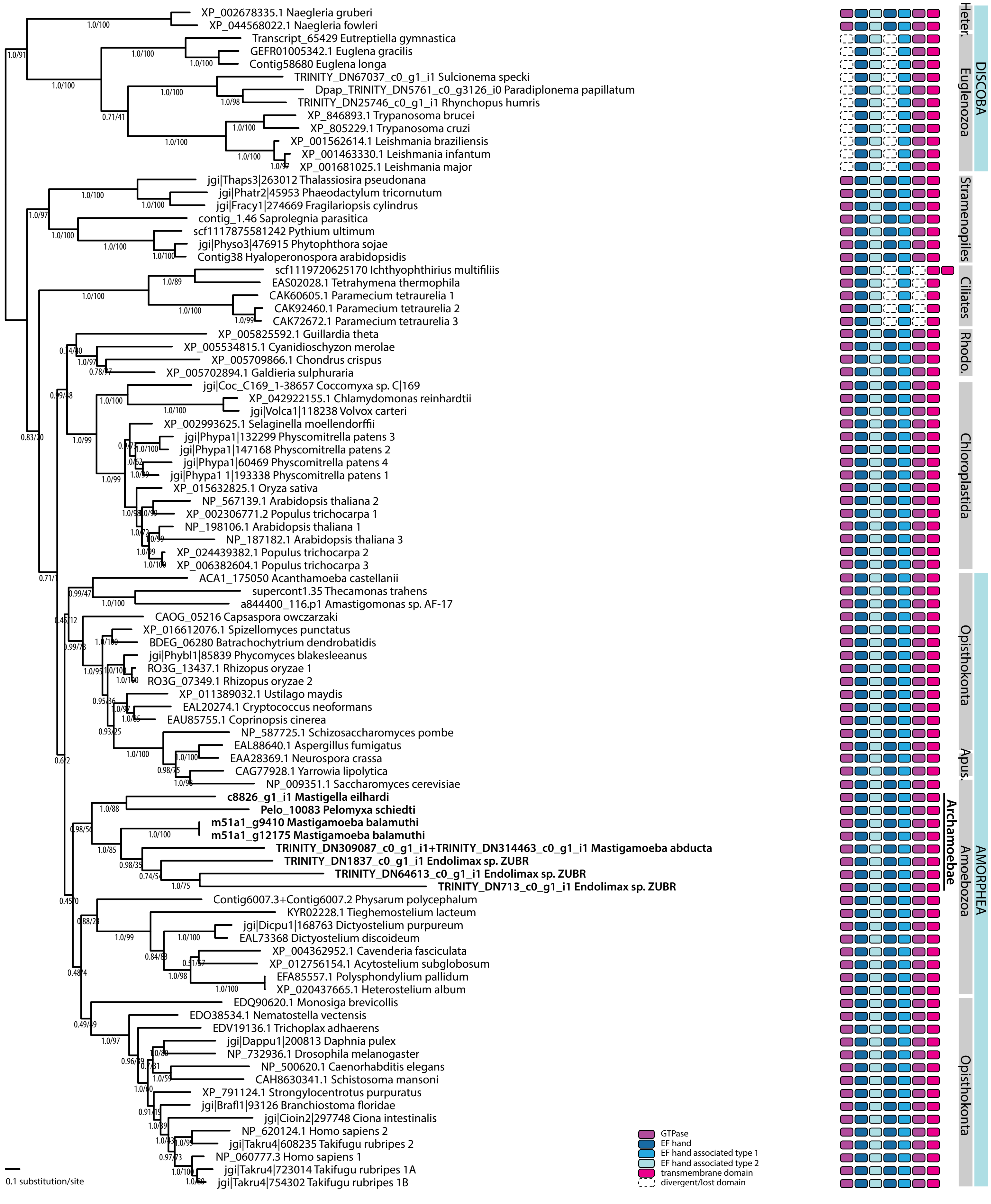

Fig. S6. Phylogenetic and domain analyses of Miro GTPase. The topology of phylogenetic tree is shown from Bayesian analysis. Support values are shown as posterior probabilities (PP) from MrBayes / bootstrap supports (BS) from RAxML. Support values for PP < 0.8 and BS < 50% are denoted by a dash (-), whereas an asterisk (*) marks a topology that was not retrieved in a particular analysis. Archamoebal sequences are in bold. Domain composition is shown as explained in the graphical legend. Miro is typically composed of an N-terminal GTPase domain, two pairs of EF hand and EF hand-associated domains, a C-proximal GTPase domain, and a C-terminal transmembrane domain. All amoebozoans, including Archamoebae, indeed exhibit this conserved architecture. In comparison, our extended dataset of euglenozoans revealed that the loss or diversification of the N-terminal GTPase domain and the second EF hand domain occurred already in the euglenozoan common ancestor, not only in trypanosomatids as noted previously (97).
